# Efficient Electrosynthesis of Hydrogen Peroxide Enabled by a Hierarchical Hollow RE–P–O (RE = Sm, La, Gd) Architecture with Open Channels

**DOI:** 10.1002/adma.202311997

**Published:** 2025-01-02

**Authors:** Zhiwei Liu, Zhaowu Wang, Diandian Lv, Hongyuan Yang, Zhenhui Kang, Suptish Ghosh, Prashanth W. Menezes, Ziliang Chen

**Affiliations:** ^1^ Institute of Functional Nano & Soft Materials (FUNSOM) Jiangsu Key Laboratory for Carbon‐Based Functional Materials & Devices Soochow University 199 Ren'ai Road Suzhou Jiangsu 215123 China; ^2^ School of Science Hebei University of Technology Tianjin 300401 China; ^3^ School of Physics and Engineering, Longmen Laboratory Henan University of Science and Technology Luoyang 471023 China; ^4^ Department of Chemistry: Metalorganics and Inorganic Materials Technische Universität Berlin Straße des 17 Juni 135, Sekr. C2 10623 Berlin Germany; ^5^ Material Chemistry Group for Thin Film Catalysis – CatLab Helmholtz‐Zentrum Berlin für Materialien und Energie Albert‐Einstein‐Str. 15 12489 Berlin Germany

**Keywords:** oxygen reduction reaction, phosphate unit, rare earth metal, reaction kinetics, selectivity and activity

## Abstract

The electrochemical two‐electron oxygen reduction reaction (2e^−^ ORR) offers a sustainable pathway for the production of H_2_O_2_; however, the development of electrocatalysts with exceptional activity, selectivity, and long‐term stability remains a challenging task. Herein, a novel approach is presented to addressing this challenge by synthesizing hierarchical hollow SmPO_4_ nanospheres with open channels via a two‐step hydrothermal treatment. The produced compound demonstrates remarkable 2e^−^ selectivity, exceeding 93% across a wide potential range of 0.0–0.6 V in 0.1 m KOH, with a peak of 96% at 0.45 V. When employed as the cathode in a flow cell, the synthesized SmPO_4_ exhibits impressive stability at 100 mA cm^−2^ for 12 h, consistently achieving a Faradaic efficiency above 90%. Using X‐ray absorption, in situ Raman and Fourier‐transform infrared spectroscopies, theoretical calculations, and post‐ORR assessments, it is found that this hollow compound possesses intrinsic open channels and is characterized by the optimal metal atomic spacing, and exceptional structural and compositional stabilities. These factors significantly enhance the thermodynamics, kinetics, and stability of the 2e^−^ ORR process. Notably, the produced compound also exhibits outstanding 2e^−^ ORR performance in neutral environments. Furthermore, this strategy can be extended to other hollow rare‐earth–P–O compounds, demonstrating excellent 2e^−^ ORR performance under both neutral and alkaline conditions.

## Graphical abstract

Rare earth phosphates with hexagonal crystal structure have been demonstrated to exhibit impressive 2e^−^ ORR performance over a wide potential and pH range, which can be attributed to their enriched open channels, large specific surface feature, optimal metal spatial distance, unwavering compositional stability as well as favorable electronic structure.

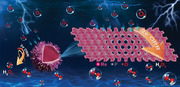

## 1 Introduction

Hydrogen peroxide (H_2_O_2_) is a fundamental chemical compound that plays a prominent role in various fields including industrial bleaching, medical disinfection, and environmental biodegradation.^[^ ^1^, ^2^ ^]^ Anthraquinones are the primary source for H_2_O_2_ production; however, this method has various drawbacks including intricate procedures, significant energy inefficiency, and adverse environmental effects that lead to a substantial carbon footprint.^[^ ^3^ ^]^ In contrast, the electrochemical synthesis of H_2_O_2_ via the two‐electron oxygen reduction reaction (2e^−^ ORR) represents a greener alternative for sustainable H_2_O_2_ production.^[^ ^4^ ^]^ Currently, electrochemically synthesized H_2_O_2_ under alkaline conditions is applied in paper bleaching and the hydrolysis of benzonitrile for benzamide production,^[^ ^5^, ^6^ ^]^ while H_2_O_2_ generated under neutral conditions can also be conveniently coupled with selective processes such as the oxidation of alkenes to epoxides and hydroxylation reactions.^[^ ^7^, ^8^ ^]^ Despite these advancements, the competition of the 2e^−^ ORR with four‐electron (4e^−^ ORR) pathways that produce water molecules significantly decreases the H_2_O_2_ generation efficiency. Therefore, the development of an electrocatalyst with exceptional selectivity toward the 2e^−^ ORR for enhancing the H_2_O_2_ production efficiency remains a pressing issue.^[^ ^9^ ^]^ Furthermore, to consistently generate significant quantities of H_2_O_2_, a 2e^−^ ORR electrocatalyst must possess outstanding activity and stability. Previously, precious metals and their alloys (*e.g*., Pt–Hg, Pd–Hg, and Au–Hg) have been employed as 2e^−^ ORR electrocatalysts owing to their remarkable activity and selectivity; however, the limited availability and high cost of these compounds have significantly impeded their widespread utilization.^[^ ^10^ ^]^ In this regard, the search for non‐precious metal‐based alternatives with high performance, selectivity, and stability has emerged as a pivotal factor in advancing the field of electrocatalytic 2e^−^ ORR for H_2_O_2_ production.

Over the past few decades, extensive research has been conducted on non‐noble transition metal‐based (such as Co, Ni, and Mn) oxides, chalcogenides, and phosphides. Accordingly, several effective strategies such as vacancy creation, polymorphic transformation, amorphization modulation, physical field regulation, and morphology tuning have been proposed for improving the 2e^−^ ORR performance.^[^ ^11^, ^12^, ^13^, ^14^, ^15^, ^16^, ^17^, ^18^, ^19^, ^20^, ^21^, ^22^ ^]^ For example, Yu. et al. reported that in the potential range of 0.2–0.5 V versus reversible hydrogen electrode (RHE), the black phosphorous‐tuned CoSe_2_ exhibited a 2e^−^ selectivity of more than 90%.^[^ ^19^ ^]^ Zhao et al. found that the 2e^−^ selectivity for Ni vacancy‐enriched Ni_2−_ *_x_* P reached 92%.^[^ ^21^ ^]^ Recently, Menezes and coworkers have synthesized amorphous nickel borides, among which the optimized variant demonstrated a 2e^−^ selectivity higher than 95%.^[^ ^22^ ^]^ Nevertheless, most reported electrocatalysts face challenges in achieving a balanced combination of activity, selectivity, and stability across a broad spectrum of pH levels and potential ranges. Moreover, because the development of 2e^−^ ORR electrocatalysts is still in its infancy, the exploration of alternative metal‐based electrocatalysts is of significant interest to researchers.

In general, the larger the spatial distance between the adjacent metal atoms in the crystal structure, the more conducive it is to the desorption of OOH species, consequently enhancing the production of H_2_O_2_ during ORR.^[^ ^23^, ^24^, ^25^ ^]^ In contrast to non‐precious transition metals, rare‐earth (RE) metals usually exhibit larger atomic radii, which result in larger metal atomic spacings in the corresponding compounds. Several recent studies have shown that RE elements can serve as highly catalytically active sites (*e.g.*, single Er atom catalyst for the CO_2_ reduction reaction and Sm_2_O_3_ for the nitrogen reduction reaction), although RE‐based compounds are commonly used as cocatalysts,^[^ ^26^, ^27^, ^28^ ^]^ suggesting the viability of employing RE species in catalysis. Meanwhile, most reported 2e^−^ ORR electrocatalysts, including oxides, sulfides, and phosphides, have unstable compositions and are prone to dissolution, thus negatively affecting the catalytic performance.^[^ ^29^, ^30^, ^31^ ^]^ Compared with these compounds, metal phosphates exhibit extremely high compositional stability over a wide pH range.^[^ ^31^, ^32^ ^]^ However, an ideal 2e^−^ ORR electrocatalyst must also contain proton or ion transfer channels, which can accelerate the protonation and mass transfer during ORR, increasing the catalytic activity.^[^ ^33^ ^]^ Interestingly, some metal phosphates are excellent proton conductors with abundant proton or ion transfer channels.^[^ ^34^, ^35^ ^]^ Thus, combining RE metal species with phosphate units to construct RE phosphate compounds with abundant open channels is a viable strategy for integrating the high activity, selectivity, and stability of 2e^−^ ORR catalysts. Unfortunately, there is a lack of research studies in this area. Furthermore, elucidating the inherent relationships between the composition, structure, and performance of these materials is crucial for the development of advanced RE‐based electrocatalysts. Inspired by the aforementioned insights, we employed a sequential phase conversion strategy in this study to synthesize hierarchical SmPO_4_ that would validate the proposed concept for the 2e^−^ ORR. The optimized SmPO_4_ with a hollow architecture exhibited a remarkable H_2_O_2_ selectivity exceeding 93% over a wide potential range from 0.0 to 0.6 V versus RHE combined with the peak selectivity of 96% at 0.45 V versus RHE in a 0.1 m KOH solution. Furthermore, this electrocatalyst maintained exceptional 2e^−^ ORR performance even after 5000 testing cycles, making it one of the most advanced transition metal‐based electrocatalysts reported up to date. In‐depth component and (micro)structural analyses, including Rietveld refinement, X‐ray absorption spectroscopy (XAS), theoretical calculations, in situ Raman spectroscopy, attenuated total reflection Fourier‐transform infrared (ATR–IR) spectroscopy, post‐ORR XAS, and transmission electron microscopy (TEM) revealed that hollow SmPO_4_ possessed a crystalline hexagonal structure with abundant open channels, ensuring suitable metal atomic spacing, fast proton/ion transfer, and ultrahigh structural and compositional stabilities, substantially improving the reaction thermodynamics, kinetics, and stability toward the 2e^−^ ORR. Moreover, such exceptional 2e^−^ ORR performance was also achieved for SmPO_4_ nanospheres in neutral media. Notably, the developed approach was applicable to other hollow RE–P–O (*e.g*., La and Gd) compounds, which demonstrated excellent 2e^−^ ORR activity, selectivity, and stability under both neutral and alkaline conditions. The findings of this study open new avenues for a better understanding and practical utilization of RE‐based electrocatalysts.

## 2 Results and Discussion

**Figure 1:**
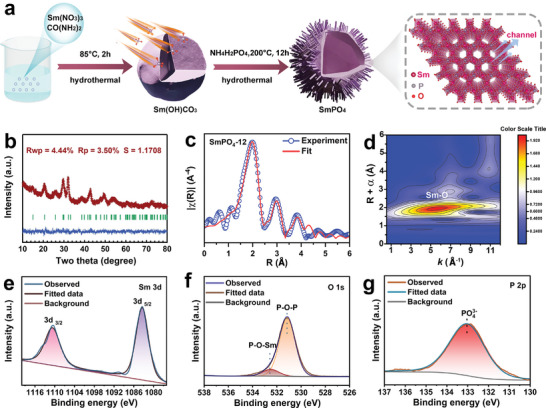
a) The schematic synthesis procedure for SmPO_4_ hollow nanosphere. b) The Rietveld refinement of XRD pattern for SmPO_4_‐12. The red line, red “+” symbol, blue line, and green vertical bars shown in the XRD pattern represent the fitted data, observed data, differentiation, and the positions of the diffraction peaks, respectively. c) Sm *L*_3_‐edge Fourier transformed (FT)‐EXAFS spectra of SmPO_4_‐12 fitted in R space, the blue and red lines represent the experimental and fitting values, respectively. d) Wavelet transform (WT) of Sm *L*_3_‐edge EXAFS for SmPO_4_‐12. High‐resolution XPS spectra of e) Sm 3d, f) O 1s, and g) P 2p in SmPO_4_‐12.

The synthesis of the hollow SmPO_4_ architecture is schematically illustrated in **Figure** **1**a. First, solid Sm(OH)CO_3_ nanospheres with smooth surfaces were prepared through a one‐pot hydrothermal treatment. Subsequently, the Sm(OH)CO_3_ precursors were phosphorized via a second hydrothermal process in the presence of NH_4_H_2_PO_4_. During this stage, an interfacial ion‐exchange reaction occurred, resulting in the formation of a hierarchical structure consisting of SmPO_4_ nanorods firmly anchored onto hollow SmPO_4_ nanospheres. The phase conversion during synthesis was examined via X‐ray diffraction (XRD). As shown in Figure S1 (Supporting Information), some new diffraction peaks at ≈30° and 40° emerged after 1 h of phosphorization and became more distinct after phosphorization for both 12 and 24 h. These newly formed diffraction peaks can be assigned to the theoretically calculated SmPO_4_ phase model with a hexagonal structure.^[^ ^36^ ^]^ To validate this conclusion, a Rietveld refinement of the XRD pattern of the phosphorized product (12 h) was performed, and the obtained results presented in Figure 1b and Tables S1 and S2 (Supporting Information) indicate the formation of a high‐purity SmPO_4_ phase with a hexagonal crystal structure (space group: *P*3_2_21). Interestingly, such a structure is assembled from [SmO_8_] dodecahedral and [PO_4_] tetrahedral units, in which four corners and two edges of one [PO_4_] tetrahedron are shared by four [SmO_8_] dodecahedra and two [SmO_8_] dodecahedra (Figure 1a), respectively. This geometric configuration not only assures large spatial distances between the adjacent Sm atoms (>4 Å) but also promotes the generation of abundant intrinsic channels with a diameter of ≈5 Å, facilitating both the desorption of the entire OOH ligand and transfer of protons during ORR.^[^ ^37^, ^38^, ^39^ ^]^ Thus, for convenience, the products obtained after 1, 12, and 24 h of phosphorization are denoted as SmPO_4_‐1, SmPO_4_‐12, and SmPO_4_‐24, respectively. Meanwhile, to confirm the deep phase transformation of Sm(OH)CO_3_ to SmPO_4_, the Sm(OH)CO_3_ precursor and its corresponding phosphorized product were examined via Fourier transform infrared (FTIR) spectroscopy. As shown in Figure S2 (Supporting Information), the bands at 3443, 1434, 1528, 1081, 844, 742, and 696 cm^−1^ can be attributed to OH (υ), CO (υas), CO (υs), CO (δ), OH (δ), and CO (δ) signals of Sm(OH)CO_3_, respectively. In contrast, the corresponding FTIR absorption bands of the phosphorized product changed significantly. Specifically, the peaks centered at 1075.0 and 967.3 cm^−1^ correspond to the stretching vibrations of PO_4_^3−^,^[^ ^40^ ^]^ while the absorption vibrations of P−O appear at 546.2 and 573.4 cm^−1^ accompanied by the disappearance of the absorption vibration peaks of Sm(OH)CO_3_. Similar results were obtained by comparing the Raman spectra of Sm(OH)CO_3_ and its phosphorized product (Figure S3, Supporting Information).^[^ ^41^, ^42^ ^]^

To gain a more comprehensive understanding of the local structural environment, X‐ray absorption spectra were obtained at the Sm *L*_3_‐edge of SmPO_4_‐12 and compared with the Sm_2_O_3_ reference spectrum (Figure S4, Supporting Information). According to Figure S4a (Supporting Information), the characteristics of the Sm *L*_3_‐edge spectra are reflected by the pre‐edge and main‐edge peaks induced by the dipole, allowing electron transition to different orbitals.^[^ ^43^ ^]^ The pre‐edge peak can be associated with the presence of distorted [SmO*_n_*] unit (*n* = 6–8) polyhedra in Sm_2_O_3_ and SmPO_4_‐12.^[^ ^44^, ^45^ ^]^ Notably, the pre‐edge peak of Sm_2_O_3_ is slightly stronger than that of SmPO_4_‐12, indicating a higher degree of distortion in the [SmO*_n_*] unit. In addition, the main‐edge peak of SmPO_4_‐12 is stronger than that of Sm_2_O_3_, which can be ascribed to the disturbance of the highly disordered [PO_4_] units by Sm species.^[^ ^46^ ^]^ Meanwhile, the refined structural model from the XRD pattern was adopted to fit the extended X‐ray absorption fine structure (EXAFS) spectra obtained for the Sm *L*_3_‐edge in SmPO_4_‐12. The refined results showed consistent structural parameters (Figure 1c; Figures S4b and S5 and Table S3, Supporting Information), which were typical for hexagonal SmPO_4_. This was also confirmed by the wavelet analysis of the EXAFS pattern (Figure 1d), in which the Sm─O and Sm─Sm signals were observed at the matched bond distances. Moreover, by comparing the EXAFS spectra obtained for the Sm *L*_3_‐edges in SmPO_4_‐12 and Sm_2_O_3_, it was found that the Sm─O bond in SmPO_4_‐12 was slightly longer than that in Sm_2_O_3_ (Figure S4b, Supporting Information). In addition to XAS, X‐ray photoelectron spectroscopy (XPS) was conducted to investigate the chemical states of the constituent elements and the surface composition of the as‐synthesized SmPO_4_‐12. The high‐resolution Sm 3d, O 1s and P 2p XPS profiles are shown in Figure 1e–g, respectively. The two distinct peaks (separated by 20.6 eV) observed at the binding energies of 1084.2 and 1111.8 eV can be assigned to Sm 3d_5/2_ and Sm 3d_3/2_, respectively (Figure 1e), which indicate the presence of Sm^3+^ species.^[^ ^47^ ^]^ The two O 1s peaks with binding energies of 531.07 and 532.17 eV suggest the presence of P─O─P and P─O─Sm bonds, respectively (Figure 1f).^[^ ^48^ ^]^ Correspondingly, the peak at 133.4 eV in the high‐resolution P 2p XPS spectrum was attributed to the phosphate group (PO_4_^3−^) with a pentavalent oxidation state (Figure 1g).^[^ ^47^, ^48^ ^]^

**Figure 2:**
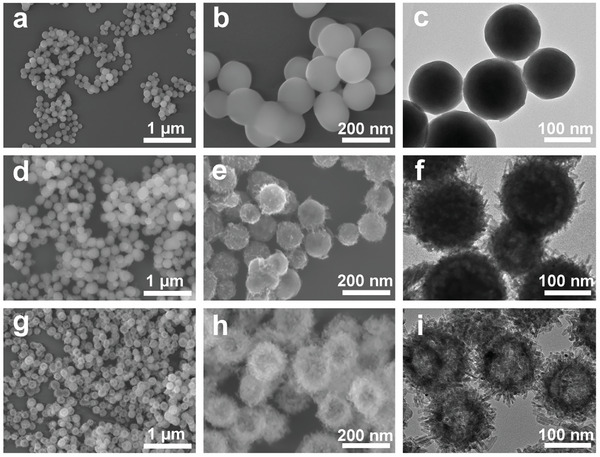
(a,b) FESEM and (c) TEM images of Sm(OH)CO_3_; (d,e) FESEM and (f) TEM images of SmPO_4_‐1 and (g,h) FESEM and (i) TEM images of SmPO_4_‐12.

To observe the morphology evolution with the phosphorization time, time‐dependent experiments were conducted during the transformation process. The field‐emission scanning electron microscopy (FESEM) and low‐magnification TEM images of the products recorded at various phosphorization times are presented in **Figure** **2**. The obtained Sm(OH)CO_3_ contains uniform monodisperse spheres with smooth surfaces and an average diameter of ≈120 nm (Figure 2a–c; Figure S6, Supporting Information). After 1 h of phosphorization, some nanorods were coated onto the nanospheres accompanied by the formation of hollow structures (Figure 2d–f; Figure S7a–c, Supporting Information), indicating the dissolution of the inner core. When the reaction time was prolonged to 12 h, the inner core underwent further dissolution, and the nanorods became more visible (Figure 2g–i). However, after 24 h of reaction, the porous shell thickened, resulting in an agglomerated morphology (Figure S7d–f, Supporting Information). This morphological evolution might follow the interface ion‐exchange reaction mechanism.^[^ ^49^ ^]^ At the initial stages, a gradual dissolution of Sm(OH)CO_3_ spheres occurs in the presence of a weakly acidic NH_4_H_2_PO_4_ solution. Sm^3+^ ions dissociate from the precursor template and directly react with PO_4_^3−^ ions to form SmPO_4_ nuclei. Because of the higher outward migration rate of Sm^3+^ metal cations with smaller radii than the inward migration rate of [PO_4_^3−^] units with larger sizes, a hollow structure is formed inside the sphere. The surfactant cetyltrimethylammonium bromide guides the anisotropic growth of SmPO_4_, leading to the formation of nanorods. Notably, in addition to the nanorod morphology and hollow features, N_2_ adsorption–desorption isotherms coupled with pore size distribution analyses revealed that all phosphate products exhibited high specific surface areas and predominantly mesoporous characteristics (Figure S8, Supporting Information). These properties not only facilitated the mass/ion transport and product escape process but also enhanced the surface wettability and active site accessibility.^[^ ^50^ ^]^

**Figure 3:**
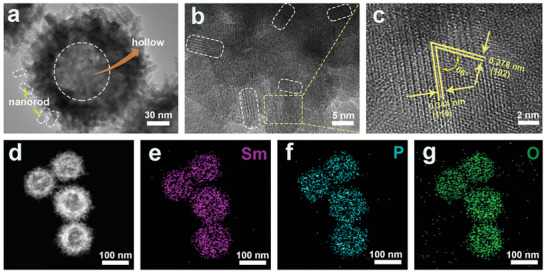
(a) TEM image of SmPO_4_‐12, (b) high‐magnification, and (c) high‐resolution TEM images of SmPO_4_‐12. (d) HAADF image of representative SmPO_4_‐12 and the corresponding elemental mappings of (e) Sm, (f) P, and (g) O species.

To elucidate microstructural details, high‐magnification and high‐resolution TEM (HRTEM) images of SmPO_4_‐12 were obtained. The magnified TEM image of SmPO_4_‐12 shows a distinct contrast along the edge to the center, confirming the hollow architecture (**Figure** **3**a). The estimated hollow cavity size is 80–100 nm, whereas the surfaces of the spheres are composed of numerous nanorods with diameters of ≈10 nm and lengths ranging from 10 to 80 nm (Figure 3b). The HRTEM image reveals the presence of lattice fringes with spacings of 0.278 and 0.344 nm, which can be directly attributed to the (110) and (102) facets of the SmPO_4_ structure, respectively (Figure 3c). Furthermore, these two crystal planes formed an inclusion angle of 66°. The selected area electron diffraction (SAED) pattern of the composite region exhibits clear bright diffraction rings corresponding to the (100), (101), (110), (200), (102), (112), (210), (103), and (113) facets of the hexagonal SmPO_4_ phase (Figure S9, Supporting Information). This confirms the hexagonal structure of SmPO_4_, which is in good agreement with the XRD and EXAFS results. Additionally, the high‐angle annular dark field–scanning TEM (HAADF–STEM) images and corresponding elemental maps suggest the homogeneous distributions of Sm, P, and O atoms throughout the entire SmPO_4_‐12 sample (Figure 3d–g). Energy‐dispersive X‐ray (EDX) spectroscopy was conducted to determine the composition of the synthesized product. Figure S10 (Supporting Information) confirms the presence of Sm, P, and O elements in the product, and the obtained Sm: P atomic ratio is close to 1:1, being in accordance with the theoretical value obtained for SmPO_4_. Similar to SmPO_4_‐12, the formation of the SmPO_4_ phase as well as the corresponding compositions of SmPO_4_‐1 and SmPO_4_‐24 were verified using their corresponding high‐magnification TEM, HRTEM, HADDF, and elemental mapping images (Figures S11 and S12, Supporting Information).

**Figure 4:**
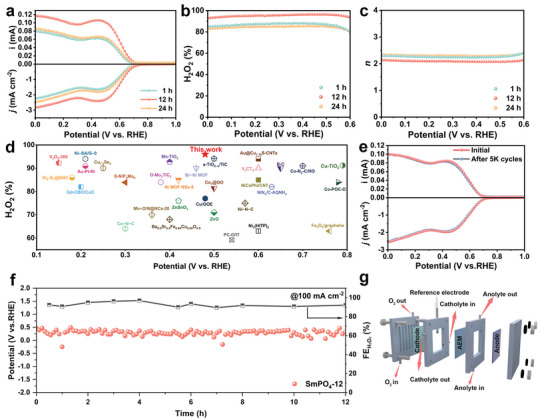
(a) LSV curves of SmPO_4_ recorded at 1600 rpm with a scan rate of 10 mVs^−1^ (bottom part), together with the corresponding H_2_O_2_ current on the ring electrode (upper part) in O_2_‐saturated 0.1 m KOH. (b) Selectivity of H_2_O_2_ and (c) calculated electron transfer number (*n*) within the potential sweep. (d) Comparison of 2e^−^ ORR activity of SmPO_4_‐12 with the previously reported metal‐based electrocatalysts in the alkaline media (for qualitative reference only). (e) LSV curves of SmPO_4_‐12 before and after 5000 ADT cycles. (f) Time‐voltage curve and implement Faraday efficiency curve under the O_2_ condition in 0.1 m KOH of SmPO_4_‐12 in gas diffusion electrode (100 mA cm^−2^). (g) Digital image for the gas diffusion electrode configuration by using SmPO_4_‐12 casted on carbon paper as an electrode.

The electrocatalytic performance of SmPO_4_ toward the 2e^−^ ORR was first assessed using a rotating ring disk electrode (RRDE) in an oxygen gas‐saturated 0.1 m KOH electrolyte at a rotation rate of 1600 rpm. Subsequently, linear sweep voltammetry curves were recorded at a scan rate of 10 mV s^−1^ to obtain the disk current density and ring current of SmPO_4_ (**Figure** **4**a). As expected, SmPO_4_‐12 exhibited a disk current density of 1 mA cm^−2^ at 0.63 V versus RHE, significantly outperforming those of SmPO_4_‐1 and SmPO_4_‐24. Moreover, SmPO_4_‐12 demonstrated the ability to reach a disk current density of 2.80 mA cm^−2^, approaching the theoretical diffusion‐limiting value of 3 mA cm^−2^ at a potential of 0.0 V versus RHE. According to the results of ring and disk current measurements, SmPO_4_‐12 exhibited a high selectivity toward H_2_O_2_, which exceeded 93% within a wide potential range from 0.0 to 0.6 V versus RHE (Figure 4b). Moreover, the 2e^−^ selectivity reached 96% at 0.45 V versus RHE, which is much higher than those of SmPO_4_‐1 (88%) and SmPO_4_‐24 (85%) within the same potential window. Additionally, SmPO_4_‐12 exhibits a lower electron transfer number (*n*) than those of SmPO_4_‐1 and SmPO_4_‐24 in the potential range from 0.0 to 0.6 V versus RHE (Figure 4c). These results indicate that the morphology of SmPO_4_ directly influences both the activity and selectivity of the 2e⁻ ORR because the optimized structure of SmPO_4_‐12 is characterized by the enhanced active surface area and efficient mass transport, leading to higher H_2_O_2_ selectivity and yield as compared with those of SmPO_4_‐1 and SmPO_4_‐24. These findings clearly demonstrate the critical role of morphology in tuning the catalytic behavior of SmPO_4_ for the 2e⁻ ORR. Notably, in the potential range from 0.3 to 0.5 V versus RHE, where the ring current more strongly reflected the production of H_2_O_2_, SmPO_4_‐12 produced an *n* value of ≈2.07, suggesting a nearly ideal two‐electron pathway. To the best of our knowledge, this exceptional 2e^–^ ORR selectivity of SmPO_4_‐12 surpasses those of most previously studied precious metal‐based and transition metal‐based 2e^–^ electrocatalysts in alkaline media (Figure 4d; Table S4, Supporting Information). It is noteworthy that although the current density was not high, the highest 2e^−^ ORR selectivity for pristine SmPO_4_‐12 exceeded 98% in alkaline media, demonstrating its extraordinary intrinsic 2e^−^ ORR selectivity (Figure S13a–c, Supporting Information). Upon the addition of a small amount of Ketjen Black (10 wt.%), the current density of the electrode remarkably increased. Note that the modified Ketjen Black exhibited lower intrinsic 2e^−^ ORR selectivity as compared to that of SmPO_4_‐12 in alkaline media (Figure S13d–f, Supporting Information). These results suggest that the modified Ketjen Black mainly promoted the dispersion and electrical connectivity of SmPO_4_‐12 particles, thus leading to the full utilization of the active sites and enhancement of the 2e^−^ ORR activity.

Because stability is a crucial parameter for assessing catalytic performance, SmPO_4_‐12 was subjected to 5000 cycles of accelerated degradation testing (ADT) to demonstrate its remarkable stability. According to Figure 4e, SmPO_4_‐12 exhibits negligible activity decay and selectivity degradation even after 5000 ADT cycles. Meanwhile, SmPO_4_‐12 was also studied via chronoamperometry (CA) for 12 h at 0.48 V versus RHE (corresponding to a current density of ≈25 mA cm^−2^) in an H‐cell apparatus (Figures S14 and S15, Supporting Information), demonstrating stable activity throughout the experiment. To examine the composition, phase structure, morphology, and microstructural stability of SmPO_4_‐12, high‐resolution Sm 3d, O 1s, and P 2p XPS profiles were recorded for post‐ORR SmPO_4_‐12 (Figure S16, Supporting Information), which were consistent with those of fresh SmPO_4_‐12, indicating exceptional stability. Additionally, FESEM images (Figure S17, Supporting Information) reveal the preservation of the hollow structure and nanorod morphology of SmPO_4_‐12 after the ORR, confirming its remarkable stability. Inspired by the impressive 2e^−^ ORR performance of SmPO_4_‐12, we explored its potential applicability for the large‐scale production of H_2_O_2._ For this purpose, SmPO_4_‐12 was deposited on carbon paper, which was subsequently assembled in a gas diffusion electrolyzer of a flow cell and underwent bulk electrolysis during 12 h of chronopotentiometry (CP) conducted at a current density of 100 mA cm^−2^ (the corresponding potential was below 0.5 V versus RHE) (Figure 4f,g; Figure S18, Supporting Information). As expected, SmPO_4_‐12 maintained stable activity after durability testing, confirming its exceptional stability. The obtained H_2_O_2_ yield was determined by a potassium permanganate titration method with periodic measurements of Faradaic efficiency (FE) and H_2_O_2_ yield at a fixed current density of 100 mA cm^−2^ during CP. As shown in Figure 4f, the FE remained above 90% throughout the 12‐h test, and the H_2_O_2_ yield of SmPO_4_‐12 reached a maximum of 3.01 mol g_cat_^−^^1^ h^−^^1^ (Figure S19, Supporting Information). Note that the bare carbon paper contribution to H_2_O_2_ production was low (Figure S20, Supporting Information). Meanwhile, to verify the accuracy of the determined H_2_O_2_ concentration, the eFOX solution‐based standard colorimetric method was employed half an hour after the reaction (Figure S21, Supporting Information), and the obtained results were close to each other.^[^ ^51^ ^]^ These observations indicate the potential applicability of SmPO_4_‐12 as a promising candidate for the alkaline electrosynthesis of H_2_O_2_.

**Figure 5:**
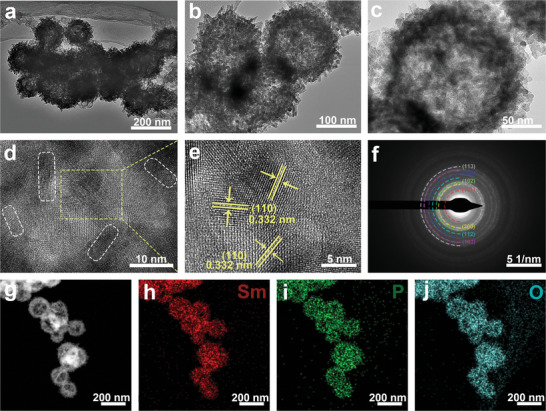
(a−c) TEM images, (d) high‐magnified and (e) high‐resolution (HRTEM) image, as well as (f) the corresponding SAED pattern of representative post‐ORR SmPO_4_‐12. (g) HAADF image of representative SmPO_4_‐12 and the corresponding elemental mappings of (h) Sm, (i) P, and (j) O species.

To examine the morphology, phase stability, and microstructural integrity of SmPO_4_‐12 after the long‐term ORR in a flow cell, a series of post‐ORR characterizations, including XRD, TEM, and XAS, were conducted. The obtained XRD patterns confirm that the phase structure remains unchanged after the stability tests (Figure S22, Supporting Information). In addition, the TEM images (**Figure** **5**a–c) demonstrate the preservation of the hollow structure coupled with the nanorod morphology of SmPO_4_‐12 after the ORR. The HRTEM image of a representative SmPO_4_‐12 sample after the ORR shows the presence of SmPO_4_ crystalline (110) facets (Figure 5d,e) with lattice spacings of 0.332 nm. In addition, the associated SAED pattern confirms the presence of the (101), (200), (102), (112), (210), (103), and (113) crystalline planes within the SmPO_4_ phase, providing strong evidence for the high stability of the crystalline phase during the electrocatalytic process (Figure 5f). Furthermore, the HAADF–STEM pattern of post‐ORR SmPO_4_‐12 along with its corresponding EDX mapping images verified the homogeneous distribution of Sm, P, and O elements within the composite (Figure 5g–j; Figure S23, Supporting Information). The XAS results obtained after the ORR stability tests in the flow cell are shown in Figure S24 and Table S5 (Supporting Information). The corresponding post‐ORR *k*‐space plots of the EXAFS spectra are presented in Figure S25 (Supporting Information). These results indicate that the local sample structure remained unchanged, demonstrating its excellent structural stability during prolonged electrolysis. Meanwhile, inductively coupled plasma data indicated that negligible amounts of Sm and P species dissolved in the electrolyte after the ORR CP studies (Table S6, Supporting Information), corroborating the high compositional stability of the catalyst. Based on these findings, we can reasonably conclude that SmPO_4_‐12 exhibits robustness in terms of its composition, phase structure, morphology, and microstructure during ORR testing. To further demonstrate the advantages of RE phosphates as catalysts for the 2e⁻ ORR, we evaluated the 2e⁻ ORR performance of Sm_2_O_3_ and compared it with that of SmPO_4_‐12. The obtained results showed that SmPO_4_‐12 exhibited significantly higher activity, selectivity, and stability for the selective O_2_ reduction to H_2_O_2_ than those of Sm_2_O_3_ (Figures S26 and S27, Supporting Information). These results indicate that PO_4_^3−^ plays a crucial role in promoting the reaction selectivity and improving the overall catalytic performance.

**Figure 6:**
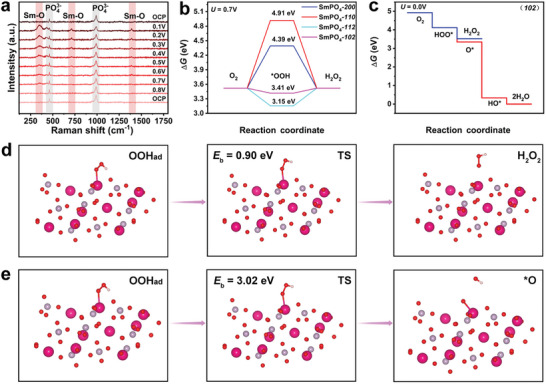
(a) In situ Raman spectra for SmPO_4_ ‐12 electrocatalyst in O_2_‐saturated 0.1 m KOH within a voltage window 0.1–0.8 V versus RHE, together with the Raman spectra backing to 0.0 V versus RHE after forward direction test from 0.1 to 0.8 V versus RHE. Free energy diagram of 2e^−^ ORR on different crystal faces of SmPO_4_ at (b) U = 0.70 V. (c) Free energy diagrams for 2e^−^ ORR and 4e^−^ ORR on (102) facet of SmPO_4_. (d) The structural models of the initial state (left), transition state (middle), and final state (right) during the pathway from ^*^OOH to H_2_O_2_. (e) The structural models of the initial state (left), transition state (middle), and final state (right) during the pathway from ^*^OOH to ^*^O. The grey, pink, red, and rose red sphere represents the P, H, O, and Sm atom, respectively.

To elucidate the catalytic mechanism of SmPO_4_ during ORR, in situ Raman measurements were performed in the potential range from 0.8 to 0.1 V versus RHE at an interval of 0.1 V. As shown in **Figure** **6**a, at the open circuit potential (OCP), two bands at 462.1 and 989.3 cm^−1^ can be assigned to the bending and stretching vibrations of the [PO_4_] tetrahedron, respectively. Upon the ORR initiation, the peaks corresponding to Sm─O bonds were observed at 351.1, 712, and 1388.8 cm^−1^, which could be attributed to the adsorption of ^*^OOH on the SmPO_4_ surface.^[^ ^40^, ^52^ ^]^ With a further increase in the ORR potential, these two peaks gradually intensified. These results suggest that Sm sites within SmPO_4_ serve as the real active sites for the adsorption and desorption of the key intermediate (OOH) during the 2e^−^ ORR. Furthermore, when the applied potential was restored to OCP, these two signals disappeared. This phenomenon suggests the high reversibility of the active Sm atoms during the ORR, thus contributing to the outstanding ORR activity, selectivity, and stability. The signal related to the Sm─Sm bond remained consistent throughout the entire ORR process, further confirming the phase stability of the metal phosphate catalyst. Moreover, in situ ATR–IR spectroscopy was also conducted to gain insights into the adsorption and desorption of the active intermediates during ORR (Figure S28, Supporting Information). As the voltage decreased from 0.8  to 0.1 V versus RHE, an adsorption peak corresponding to OOH was clearly observed at 1240 cm^−1^, indicating the formation of this intermediate. Simultaneously, a peak at 1380 cm^−1^ was detected for HOOH,^[^ ^53^, ^54^ ^]^ which gradually intensified as the voltage decreased, further confirming the accumulation of HOOH. After allowing the system to rest for 30 min, ATR–IR measurements under OCP were conducted again, revealing a noticeable weakening of both characteristic peaks, clearly demonstrating the desorption of the OOH intermediates. Additionally, an adsorption peak for H_2_O was detected at 3230 cm^−1^,^[^ ^53^, ^55^ ^]^ suggesting that the adsorbed H_2_O served as the proton source during H_2_O_2_ formation.^[^ ^53^ ^]^ These results verify the dynamic adsorption and desorption of the intermediates during the reaction and are consistent with the proposed reaction mechanism.

Based on the in situ Raman and ATR–IR data, density functional theory (DFT) calculations were performed to examine the catalytic mechanism of SmPO_4_ toward the 2e^−^ ORR. First, different layers derived from different crystal facets of SmPO_4_ were built as a catalyst model (Figure S29, Supporting Information). Various SmPO_4_ surfaces with different terminal atoms were constructed, and energy calculations were conducted to find stable structures that could be more appropriately used in the subsequent calculations (Figure S30, Supporting Information). An ideal 2e^−^ ORR electrocatalyst should possess the optimum free adsorption energy of ^*^OOH (∆G_*OOH_, ^*^ representing the catalytic site).^[^ ^56^ ^]^ The adsorption energy of ^*^OOH on Sm atoms was calculated as metal atoms usually serve as catalytic sites. Free energy diagrams during the 2e^−^ ORR were constructed for different facets of SmPO_4_ under the standard conditions (U = 0.7 V, Figure 6b) and at the equilibrium potential of the 2e^−^ ORR (U = 0.0 V, Figure S31, Supporting Information). Notably, the ∆G_*OOH_ values computed at 0.70 V for the (200), (110), (112), and (102) crystal facets were 4.39, 4.91, 3.15, and 3.41 eV, respectively (Figure 6b, the corresponding ∆G_*OOH_ values at 0.0 V are presented in Figure S32, Supporting Information). Notably, the ∆G_*OOH_ value determined for the (102) crystal facet was close to the ideal value (3.52 eV). Furthermore, an energy diagram of SmPO_4_ (102) was obtained for the 4e^–^ ORR pathway from O_2_ to H_2_O (Figure 6c). According to this diagram, SmPO_4_ exhibits only a slightly favorable tendency to proceed via the 4e^–^ ORR pathway. The highly preferable 2e^–^ ORR pathway can be selected owing to the more favorable reaction kinetics of SmPO_4_ toward the 2e^–^ ORR.^[^ ^57^ ^]^ To clarify this issue, two reaction pathways were considered: from ^*^OOH to H_2_O_2_ and from ^*^OOH to ^*^O, and their kinetic reaction barriers (E_b_) were calculated. According to Figure 6d,e, the E_b_ value obtained for the pathway from ^*^OOH to H_2_O_2_ is only 0.90 eV, which is significantly lower than that for the pathway from ^*^OOH to ^*^O (3.02 eV), indicating a high selectivity for the 2e^−^ ORR. This result strongly suggests that the unique SmPO_4_ structure not only ensured the exceptional free adsorption energy of ^*^OOH during the 2e^−^ ORR, but also considerably decreased the kinetic energy barrier for the formation of H_2_O_2_. Note that O and P atoms have also been considered as active sites in our calculations; however, the results obtained for the relaxed structural configuration revealed that both atoms could not serve as active sites in our case (details are provided in Figure S33, Supporting Information).

**Figure 7:**
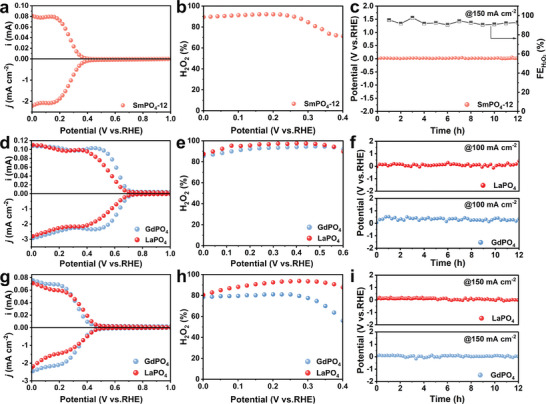
(a) LSV curves of SmPO_4_‐12 recorded at 1600 rpm with a scan rate of 10 mV s^−1^ (bottom part), together with the corresponding H_2_O_2_ current on the ring electrode (upper part) in O_2_‐saturated 0.1 K_2_SO_4_. (b) Selectivity of H_2_O_2_ and (c) Time‐voltage and Faraday efficiency curve under O_2_ conditions in 1 m Na_2_SO_4_ for SmPO_4_‐12 in a gas diffusion electrode (150 mA cm^−2^). (d) LSV curves of GdPO_4_ and LaPO_4_ were recorded at 1600 rpm with a scan rate of 10 mVs^−1^ (bottom part), together with the corresponding H_2_O_2_ current on the ring electrode (upper part) in O_2_‐saturated 0.1 m KOH. (e) Selectivity of H_2_O_2_ and (f) Time‐voltage curve under O_2_ conditions in 0.1 m KOH for LaPO_4_ and GdPO_4_ in a gas diffusion electrode (100 mA cm^−2^). (g) LSV curves of GdPO_4_ and LaPO_4_ recorded at 1600 rpm with a scan rate of 10 mV s^−1^ (bottom part), together with the corresponding H_2_O_2_ current on the ring electrode (upper part) in O_2_‐saturated 0.1 m K_2_SO_4_. (h) Selectivity of H_2_O_2_ and (i) Time‐voltage curve under the O_2_ conditions in 1 m Na_2_SO_4_ for LaPO_4_ and GdPO_4_ in a gas diffusion electrode (150 mA cm^−2^).

Because the electrosynthesis of H_2_O_2_ in neutral media is crucial for future on‐site applications, we also evaluated the ORR performance of SmPO_4_ in a 0.1 m K_2_SO_4_ solution (**Figure** **7**a,b; Figures S34–S37, Supporting Information). The obtained results indicated that in a neutral medium, SmPO_4_‐12 exhibited the highest selectivity of ≈90% and superior activity close to 2.5 mA cm^−2^ outperforming SmPO_4_‐1 and SmPO_4_‐24. To confirm its remarkable stability, SmPO_4_‐12 was subjected to a 12‐h durability test (CP) in a neutral media at a high current of 150 mA cm^−2^ in the flow cell (the corresponding potential was close to 0 V vs RHE). According to Figure 7c, SmPO_4_‐12 demonstrated outstanding stability throughout the entire 12‐h test with an FE consistently above 90%, and the H_2_O_2_ yield of SmPO_4_‐12 reached a maximum of 4.56 mol g_cat_^−^^1^ h^−^^1^ (Figure S38, Supporting Information). These results unequivocally illustrated the advantages of using the as‐prepared SmPO_4_ catalyst for the 2e^−^ ORR, which included the high specific surface area combined with abundant pores, hollow structure enriched with intrinsic proton/ion channels, appropriate spatial distance between the adjacent metal atoms, and high structural and compositional stabilities. These properties synergistically facilitated the exposure of active sites, enhanced ability to against ORR cycling, optimized the free adsorption energy of ^*^OOH, and reduced the reaction kinetic barrier for the desorption of OOH from the active sites, thus concurrently achieving high activity, selectivity, and durability toward the 2e^−^ ORR. The performance of SmPO_4_‐12 under acidic conditions was also evaluated (Figure S39, Supporting Information). The H_2_O_2_ selectivity under acidic conditions reached 80%; however, its value was lower than those obtained in the alkaline and neutral media. The inferior performance of SmPO_4_‐12 is mainly attributed to the poor chemical stability of SmPO_4_ in low‐pH environments,^[^ ^36^ ^]^ which may affect its structural stability and proton transport efficiency (Figure S40, Supporting Information).

To further validate the high application potential of RE phosphates, we utilized the same sequential phase conversion strategy to obtain LaPO_4_ and GdPO_4_ with similar crystal structures and morphologies (Figures S41–S45, Supporting Information). Subsequently, the as‐synthesized LaPO_4_ and GdPO_4_ were subjected to 2e^−^ ORR tests in 0.1 m KOH and 0.1 m K_2_SO_4_ solutions, respectively. The obtained results revealed that the RE phosphates synthesized via this strategy exhibited outstanding 2e^−^ ORR performance. Under the alkaline conditions, the selectivities of both LaPO_4_ and GdPO_4_ exceeded 90% in the potential range of 0.2–0.6 V versus RHE with a peak activity reaching 3 mA cm^−2^ (Figure 7d,e; Figure S46, Supporting Information). Remarkably, their activity remained essentially unchanged in the flow cell even after 12 h of durability testing at a high current (Figure 7f), highlighting their exceptional stability. Furthermore, under the neutral conditions, LaPO_4_ and GdPO_4_ also exhibited outstanding selectivity, activity, and stability (Figure 7g–i; Figure S47, Supporting Information).

Inspired by the success of our synthesis experiments, we expanded our investigation to include three additional RE phosphates: CePO_4_, EuPO_4_, and TbPO_4_. XRD patterns confirmed their crystalline structures, ensuring high phase purity (Figure S48, Supporting Information). Next, we evaluated their performance toward the 2e⁻ ORR under both alkaline and neutral conditions (Figure S49, Supporting Information). Interestingly, all three phosphates exhibited excellent 2e⁻ ORR activity and selectivity. These results demonstrate the robustness and broad application potential of the RE phosphate‐based electrocatalysts developed using our strategy for the 2e⁻ ORR. Furthermore, we explored the relationship between the selectivity and activity of RE metal species at the maximum activity potential (0 V versus RHE) and the ionic radius (Figure S50, Supporting Information). Specifically, we investigated the influence of the ionic radius of different RE elements on their electrocatalytic performance. The obtained results indicated that as the ionic radius increased, the catalytic activity gradually decreased. However, the selectivity trend remained relatively stable without clear regular patterns. Furthermore, these results did not show any significant correlations or trends. Although the size effects appear to influence the catalytic activity to some extent, the underlying mechanisms are more complex. In addition to the size, other factors, such as the chemical properties of the constituent elements (*e.g.*, electronic structure and oxygen affinity) may also play important roles in determining both the catalyst activity and selectivity. Furthermore, external factors, such as the properties of the electrolyte and reaction conditions, can be critical in selectivity modulation. Therefore, future studies should modulate these variables to achieve a more comprehensive understanding of the performance differences between these RE phosphate compounds in various reaction environments.

## 3 Conclusion

In this study, we have successfully used a sequential phase‐conversion strategy involving a two‐step hydrothermal reaction to prepare hollow SmPO_4_ nanospheres for efficient electrocatalytic H_2_O_2_ synthesis. Advanced XAS and in situ Raman characterizations combined with DFT calculations revealed that the hexagonal crystal structure enriched with proton/ions channels, hollow hierarchical features, appropriate spatial distances between the adjacent Sm atoms, high compositional stability, and Sm electronic configuration formed by the surrounding PO_4_^3−^ units synergistically generated abundant active sites on the SmPO_4_ surface, optimized the adsorption free energy of the ^*^OOH intermediate, lowered the reaction kinetic barrier for the desorption of OOH, and increased the structural stability of the catalyst, resulting in the superior activity, selectivity, and durability toward the 2e^−^ ORR under both alkaline and neutral conditions in a wide potential range. Most importantly, our versatile synthetic strategy can be easily applied to the production of other RE phosphate electrocatalysts, which demonstrate remarkable 2e^−^ ORR performance in both neutral and alkaline environments across a wide potential range. The discovery of RE phosphates with such exceptional catalytic performance opens new avenues for advancing the development of high‐performance 2e^−^ ORR electrocatalysts and may help achieve a deeper scientific understanding of RE‐based catalysis.

## Conflict of Interest

The authors declare no conflict of interest.

## Supporting information



Supporting Information

## Data Availability

The data that support the findings of this study are available from the corresponding author upon reasonable request.
