# Supplementary material for: Efficient Electrosynthesis of Hydrogen Peroxide Enabled by a Hierarchical Hollow RE–P–O (RE = Sm, La, Gd) Architecture with Open Channels
Source: Adv Mater. 2025 Jan 2;37(7):2311997. doi: 10.1002/adma.202311997 (PMC11837885; doi:10.1002/adma.202311997)
Supplement: Supplementary file 1 — Supporting Information [file ADMA-37-2311997-s001.docx]

Supporting Information

**Efficient Electrosynthesis of Hydrogen Peroxide Enabled by a Hierarchical Hollow RE–P–O (RE = Sm, La, Gd) Architecture with Open Channels**

*Zhiwei Liu, Zhaowu Wang, Diandian Lv, Hongyuan Yang, Zhenhui Kang,* Suptish Ghosh, Prashanth W. Menezes,* Ziliang Chen**

Z. Liu, D. Lv, Prof. Z. Kang, Dr. Z. Chen

Institute of Functional Nano & Soft Materials (FUNSOM), Jiangsu Key Laboratory for Carbon-Based Functional Materials & Devices, Soochow University, 199 Ren'ai Road, Suzhou, 215123, Jiangsu, China.

E-mail: zhkang@suda.edu.cn, zlchen@suda.edu.cn

H. Yang, S. Ghosh, Dr. P. W. Menezes

Department of Chemistry: Metalorganics and Inorganic Materials, Technische Universität Berlin, Straße des 17 Juni 135, Sekr. C2, 10623 Berlin, Germany

E-mail: prashanth.menezes@mailbox.tu-berlin.de

Dr. P. W. Menezes, Dr. Z. Chen

Material Chemistry Group for Thin Film Catalysis – CatLab, Helmholtz-Zentrum Berlin für Materialien und Energie, Albert-Einstein-Str. 15, 12489 Berlin, Germany

E-mail: prashanth.menezes@helmholtz-berlin.de

Dr. Z. Wang

School of Science, Hebei University of Technology, Tianjin 300401, China.

School of Physics and Engineering, Longmen Laboratory, Henan University of Science and Technology, Luoyang, 471023, China

**Experimental section**

**Chemicals**

Samarium nitrate hexahydrate (Sm(NO_3_)_3_·6H_2_O), Samarium oxide (Sm_2_O_3_), Lanthanum nitrate hexahydrate (La(NO_3_)_3_·6H_2_O), Cerium nitrate hexahydrate (Ce(NO_3_)_3_·6H_2_O) and Gadolinium nitrate hexahydrate (Gd(NO_3_)_3_·6H_2_O) were purchased from Aladdin Ltd (Shanghai, China). Terbium nitrate hexahydrate (TbNO_3_)_3_·6H_2_O) and Europium Nitrate (EuNO_3_)_3_) were purchased from J&K Scientific (Shanghai, China) and Macklin (Shanghai, China), respectively. Cetyl trimethyl ammonium bromide (CTAB) was purchased from J&K Scientific (Shanghai, China). Urea (CO(NH_2_)_2_) was purchased from China National Medicines Corporation Ltd. Ammonium dihydrogen phosphate (NH_4_H_2_PO_4)_ was purchased from China National Medicines Corporation Ltd. Nafion PFSA Polymer Dispersions D520 (5%) was purchased from Sinero. The water utilized in all experiments was purified through a Millipore system. All the chemicals were employed in their received form and without the need for further purification.

**Preparation of** **Sm(OH)CO_3_ nanosphere**

Sm(OH)CO_3_ colloidal spheres were synthesized using the urea-based homogeneous precipitation approach. 1 ml of Sm(NO_3_)_3_ (1 M) and 3 g of urea [CO(NH_2_)_2_] were dissolved in 50 ml of deionized water. The resulting solution was subjected to a 2 h reaction at 85 °C. After natural cooling, the collected precipitate underwent multiple washes, alternately using distilled water and ethanol. Subsequently, it was dried at 60 °C for 12 h to yield the Sm(OH)CO_3_ colloidal spheres.

**Synthesis of SmPO_4_ hollow architecture**

In a typical synthesis procedure, 1 mmol of Sm(OH)CO_3_ precipitate underwent ultrasonic treatment for 30 minutes and was then dispersed in deionized water. 0.115 g of NH_4_H_2_PO_4_, dissolved in an appropriate volume of deionized water, was then introduced into the dispersion. Then, 0.1 g of cetyltrimethylammonium bromide (CTAB) was added to this suspension. The resulting liquid was homogeneously mixed by vigorous magnetic stirring for a duration of 10 minutes. Subsequently, the reaction solution was hydrothermally treated at 200 °C for 12 h, resulting in the formation of SmPO_4_ hollow architecture. The hydrothermal treatment duration at 200 °C was varied, resulting in two distinct products denoted as SmPO_4_-1 (1-hour treatment) and SmPO_4_-24 (24-hour treatment). Similar processes were employed to create LaPO_4_, GdPO_4_ CePO_4_, EuPO_4_ and TbPO_4_ hollow architecture. In the initial stage, La(NO_3_)_3_, Gd(NO_3_)_3_, Ce(NO_3_)_3_, Eu(NO_3_)_3_ and Tb(NO_3_)_3_ aqueous solutions were employed as precursors instead of Sm(NO_3_)_3_ solution in stoichiometric quantities. Following the synthesis, the white products of SmPO_4_, LaPO_4_, GdPO_4_ CePO_4_, EuPO_4_ and TbPO_4_ hollow architecture were carefully collected by washing with deionized water and ethanol, and then air-dried at 60 °C for 24 hours. As for CePO_4_, the basic carbonates templates cannot be fully converted to phosphates by hydrothermal reaction in NH_4_H_2_PO_4_, thus, the products need to be treated with 0.1 M HNO_3_ is needed to remove the templates.

**Characterization**

The phase structure and composition of the prepared samples were determined using powder X-ray diffraction (XRD) analysis, which was performed on an XPert Powder X-ray diffractometer with Cu *K*_α_ radiation (*λ* = 1.5406 Å). The obtained XRD profiles were subsequently subjected to detailed analysis using the Rietveld refinement program RIETAN-2000.^[1]^ To characterize the elemental chemical states of the samples, X-ray photoelectron spectrometry (XPS) was carried out, utilizing an ESCALAB 250Xi spectrometer (Thermo Scientific, USA) equipped with a pass energy of 30 eV and powered at 100 W (10 kV and 10 mA). A monochromatized Al*K*_α_ X-ray source (*hν* = 1486.65 eV) was employed. All sample analyses were conducted under a vacuum pressure of less than 1.0×10^−9^ Pa. Spectra were acquired using the advantage software (Version 5.979) with an energy step of 0.05 еV. The morphology and microstructure of the samples were investigated using field-emission scanning electron microscopy (FESEM) with a Zeiss G500 instrument and transmission electron microscopy (TEM) with an FEI Talos F200X instrument. *In-situ* Raman spectra were recorded during the oxygen reduction reaction (ORR) process using a Raman spectrometer with an excitation wavelength of 532 nm (LabRAM HR Evolution). Fourier transform infrared (FTIR) spectra were recorded using a Thermo Scientific Nicolet iS50 in attenuated total reflectance (ATR-IR) mode with a resolution of 4 cm^−2^ at room temperature. The in-situ experiments were conducted using an ECIR-II cell equipped with a Pike Veemax III ATR accessory, part of a three-electrode system obtained from Shanghai Linglu Instrument & Equipment Co. To gain a deep understanding of the local atomic structure of our catalyst, we performed X-ray absorption spectroscopy (XAS) measurements for SmPO_4_ powders at the Sm *L*_3_-edge using the XAFCA beamline at the Synchrotron Light Source (SSLS). We used an electron beam with a current below 200 mA and an energy of 700 MeV. Data was collected in transmission mode with an ion chamber detector, and a Si 111 monochromator was used for beam energy tuning. The local atomic structure of the SmPO_4_ electrode after 12 hours of ORR cycling in a flow cell was investigated using synchrotron radiation fluorescence modeling. The *L*-edge XAS spectra were acquired at the Shanghai Synchrotron Radiation Facility (SSRF) with a Si(111) double-crystal monochromator at the BL14W1 beamline. All *L*-edge XAS data were analyzed using the program Demeter. X-ray fluorescence spectrometer based on Rowland circle geometry on the BL14W1 beamline. The elemental contents of the samples were determined using Inductively coupled plasma-optical emission spectra (ICP-OES) tests (Varian 720-ES).

**Electrochemical measurements**

Initially, 3.6 mg of the prepared electrocatalyst and 0.4 mg of Ketjen Black were combined and dispersed in a solution mixture consisting of 600 μL of H_2_O, 300 μL of ethanol, and 100 μL of 0.5 wt.% Nafion solution. To achieve a homogeneous ink, the mixed solution was sonicated for 30 minutes in an ice water bath. Subsequently, 6.20 μL of the sonicated solution was carefully deposited onto the glassy carbon electrode of the rotating ring-disk electrode (RRDE), which had an electrode area of 0.1256 cm^2^. The RRDE, specifically the RRDE-3A model with a fixed-disk (disk OD = 4.0 mm) and a Pt ring (ring OD = 7.0 mm), was employed. To evaluate the electrochemical performance of the catalysts, an electrochemical workstation (760E, CHI) was employed. A standard three-electrode system was established for this purpose. In this setup, the RRDE loaded with catalysts was the working electrode, while Hg/HgO or Hg/HgCl_2_ and a graphite rod were employed as the reference electrode and counter electrode, respectively. The electron transfer number (*n*) and H_2_O_2_ selectivity (H_2_O_2_%) were calculated using the following equations:

$n = 4\frac{I_{D}}{I_{D}+I_{R}/N}$ (1)

$H_{2}O_{2}(\%) = 200\frac{I_{R}/N}{I_{D}+I_{R}/N}$ (2)

where *I_R_* represents the ring current, *I_D_* represents the absolute value of disk current, and the collection efficiency (*N*) of the RRDE has a theoretical value of 0.37. Note that all Ketjen black used in this work has been annealed in a tube furnace at 250 °C for two hours under a mixture of argon and hydrogen and then naturally cooled.

**Electrochemical measurements in gas diffusion electrode**

The electroreduction process was further carried out utilizing a gas diffusion electrode, which consisted of a coated SmPO_4_-12 catalyst serving as the working electrode, an anion exchange membrane, and commercial nickel foam functioning as the anode. The working electrode was prepared by spraying the catalyst onto a gas diffusion layer (CETECH, NIS1007). The catalyst ink was prepared by mixing 4.5 mg catalyst with 0.5 mg Ketjen black, followed by dispersion in a solution of 100 μL 5 wt.% Nafion, 600 μL H_2_O and 300 μL ethanol. Prior to spraying, the ink underwent ultrasonic treatment for 30 minutes and was subsequently sprayed onto a gas diffusion layer (1 × 3 cm^2^) with a controlled loading capacity of 0.5 mg cm^−2^. The working electrode, an anion exchange membrane, and a nickel foam anode were then assembled together with a Polytetrafluoroethylene gasket. The liquid electrolyte was introduced into chambers between the anode and the membrane and between the membrane and the cathode. Gaseous O_2_ diffused from behind the gas diffusion layer to the liquid electrolyte in the catalytic region (0.5 × 2.0 cm^2^). All electrochemical experiments were performed using a CHI760 (Chenhua, Shanghai) electrochemical workstation. The electrolyte (Alkaline: 200 mL of 0.1 M KOH solution for both cathode and anode; Neutral: 200 mL of 1 M Na_2_SO_4_ and 1 M KOH solution for cathode and anode, respectively) was circulated in the electrochemical cell using a peristaltic pump at a flow rate of 25 mL min⁻^1^. The O_2_ flow rate was maintained at 30 mL min⁻^1^ by a mass flow controller. The reactions were tested by chronopotentiometry at 100 or 150 mA cm⁻^2^ without ohmic *IR* drop correction. H_2_O_2_ productivity was quantified using potassium permanganate (KMnO_4_) titration method. Specifically, 2 mL of the catholyte was acidified with 2 mL of H_2_SO_4_, followed by titration with a 0.02 mol L⁻^1^ KMnO_4_ solution. It is crucial to remind that the color change in the acidified catholyte started when the last drop of KMnO_4_ standard solution was added and persisted consistently for half a minute. This method was used to calculate the total amount of H_2_O_2_ based on KMnO_4_ consumption. The Faraday efficiency (FE) and yield rate (YR) for H_2_O_2_ generation in the gas diffusion cell were calculated as follows:

$FE(\%) = \frac{2\times96500\times M_{H_{2}O_{2}}}{C}\times100\%$ (3)

$YR = \frac{M_{H_{2}O_{2}}}{A\times t\times g_{cat}}$ (4)

Where *M_H2O2_* is the mole of generated H_2_O_2_, *C* is the totally consumed charges, *A* is the area of the cathode electrode, t is the electrolysis duration and g_cat_ is the mass of the catalyst.

Furthermore, to ensure the accuracy of the determination on the concentration of H_2_O_2_, the concentration of H_2_O_2_ was measured using a standard colorimetric method with the eFOX solution. To prepare the eFOX solution, Fe(NH_4_)_2_(SO_4_)_2_·6H_2_O (98.05 mg), D-sorbitol (18.20 mg), and xylenol orange (71.65 mg) were dissolved in a mixture of 1000 mL of ultrapure H_2_O, 10 mL of ethanol, and 1.36 mL of 98% H_2_SO_4_. Following illumination, 50 μL of the resulting solution was combined with 10 mL of the prepared eFOX solution and allowed to sit for 1 hour. Subsequently, the concentration of H_2_O_2_ was determined by measuring the characteristic absorption of the mixture at 554 nm using UV-vis spectroscopy, in accordance with a calibration curve, thereby enabling the quantitative determination the concentration of H_2_O_2_. The calibration curve is obtained in the previous work: $a=0.000414603\times C_{1}+0.00327$, *a* represents absorbance for solution, and *C_1_* means H_2_O_2_ concentration in the solution.

**Electrochemical stability measurement of H-cell electrolyzer**

Carbon paper, serving as the support for the catalyst, functioned as the working electrode with a loading of 0.5 mg cm^−2^, while platinum foil acted as the counter electrode. The stability assessment of the catalyst was conducted in a 1 M KOH solution. The catalyst ink was prepared by mixing 4.5 mg catalyst with 0.5 mg Ketjen black, followed by dispersion in a solution of 100 μL 5 wt.% Nafion, 600 μL H_2_O and 300 μL ethanol. All electrochemical experiments were performed on a CHI760 electrochemical workstation (Chenhua, Shanghai). The reactions were performed using chronoamperometry technique, which measured the stability of the catalyst at 25 mA cm^−2^ without ohmic drop correction.

**Theoretic calculations**

Density functional theory (DFT) calculations were conducted to investigate the catalytic properties of SmPO_4_ using the Vienna Ab-Initio Simulation Package.^[2]^ The Core electrons were described by pseudopotentials generated from the projector augmented wave method and the exchange and correlation terms were described using general gradient approximation (GGA) in the scheme of Perdew-Burke-Ernzerhof (PBE).^[3, 4]^ A cut-off energy of 450 eV was employed for the plane-wave basis. Guided by the XRD and TEM findings, (200), (110) and (112) and (102) surfaces were selected to investigate the ORR properties of SmPO_4_. All slab models of the surface were built with a vacuum layer of 18 Å along the Z-axis direction to avoid interaction caused by periodic boundary conditions. During structure optimization, all energy change criterion was set to 10^−4^ eV, the atoms were relaxed until the force acting on each atom was less than 0.02 eV Å^−1^. Climbing Image Nudged Elastic Band (CI-NEB) method^[5]^ was used to find the minimum energy paths and the transition states. Surfaces of SmPO_4_ with different possible terminal atoms were built, and their energies were calculated to identify stable configurations for subsequent calculations (Figure S30). The reaction free energy Δ*G* for each step of ORR was defined as the difference between free energies of the initial and final states and was calculated by the following equation:

$\text{Δ}G = \text{Δ}E+\text{Δ}ZPE-T\text{Δ}S+\text{Δ}G_{U}+\text{Δ}G_{\text{pH}}$ (5)

Where *ΔE* represents the reaction energy of reactant molecules adsorbed on the catalyst surface; *ZPE* and *S* mean the zero-point energy and entropy, respectively; Δ*G_U_* = *-neU*; Δ*G_PH_* is the correction of the H^+^ free energy.

**Figures**

**
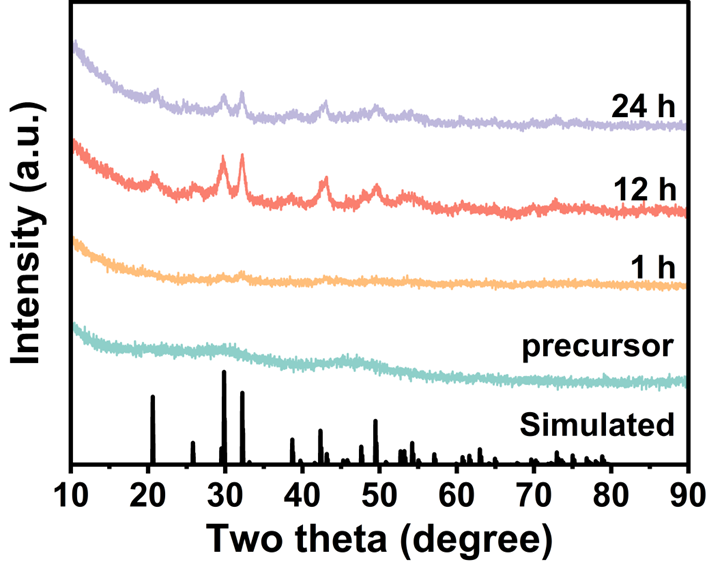
**

**Figure S1**. XRD patterns of the precursor (Sm(OH)CO_3_) as well as its phosphate products after 1, 12, and 24 h of phosphorization, respectively.


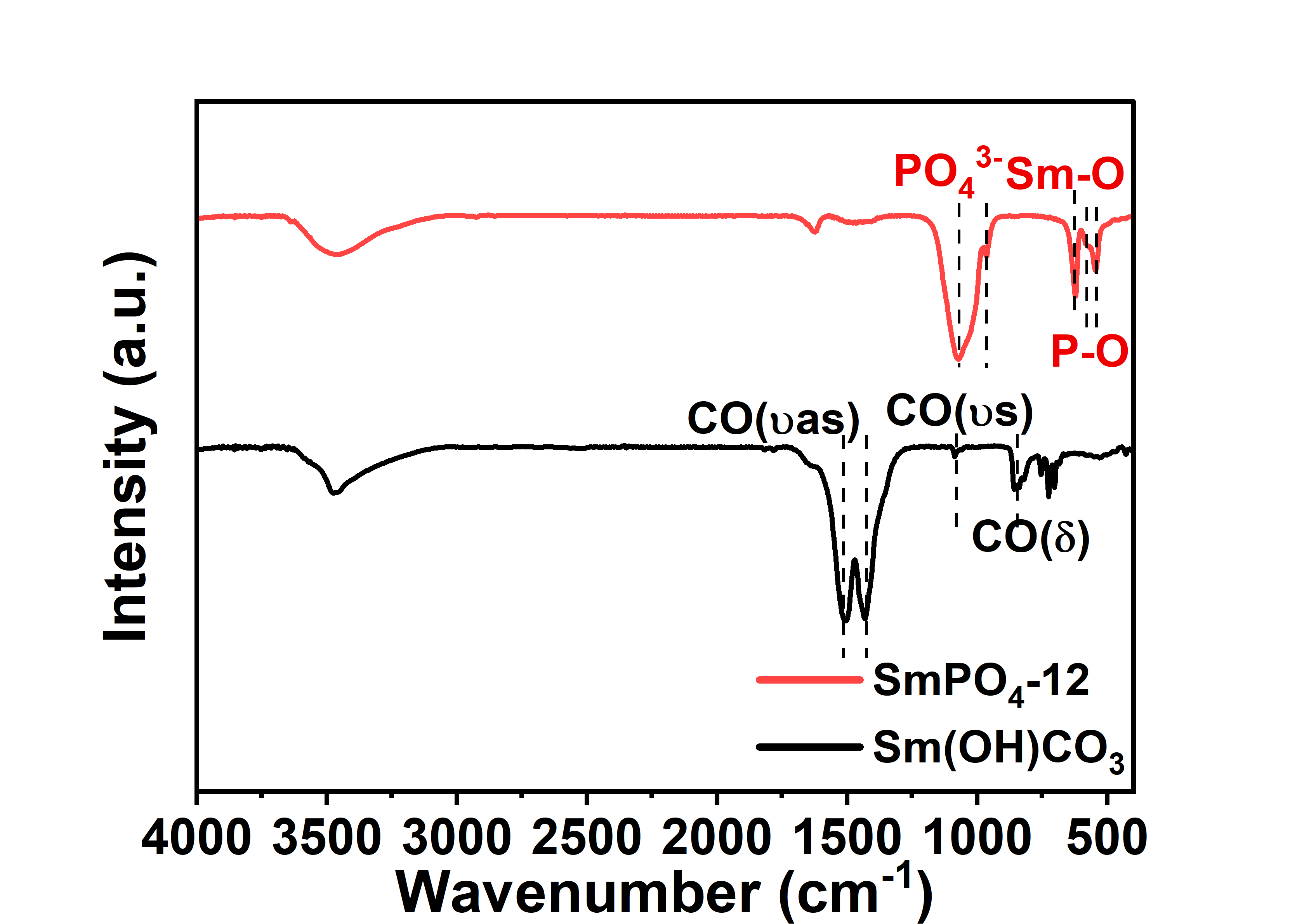


**Figure S2**. FTIR spectra of Sm(OH)CO_3_ and SmPO_4_-12 powder.


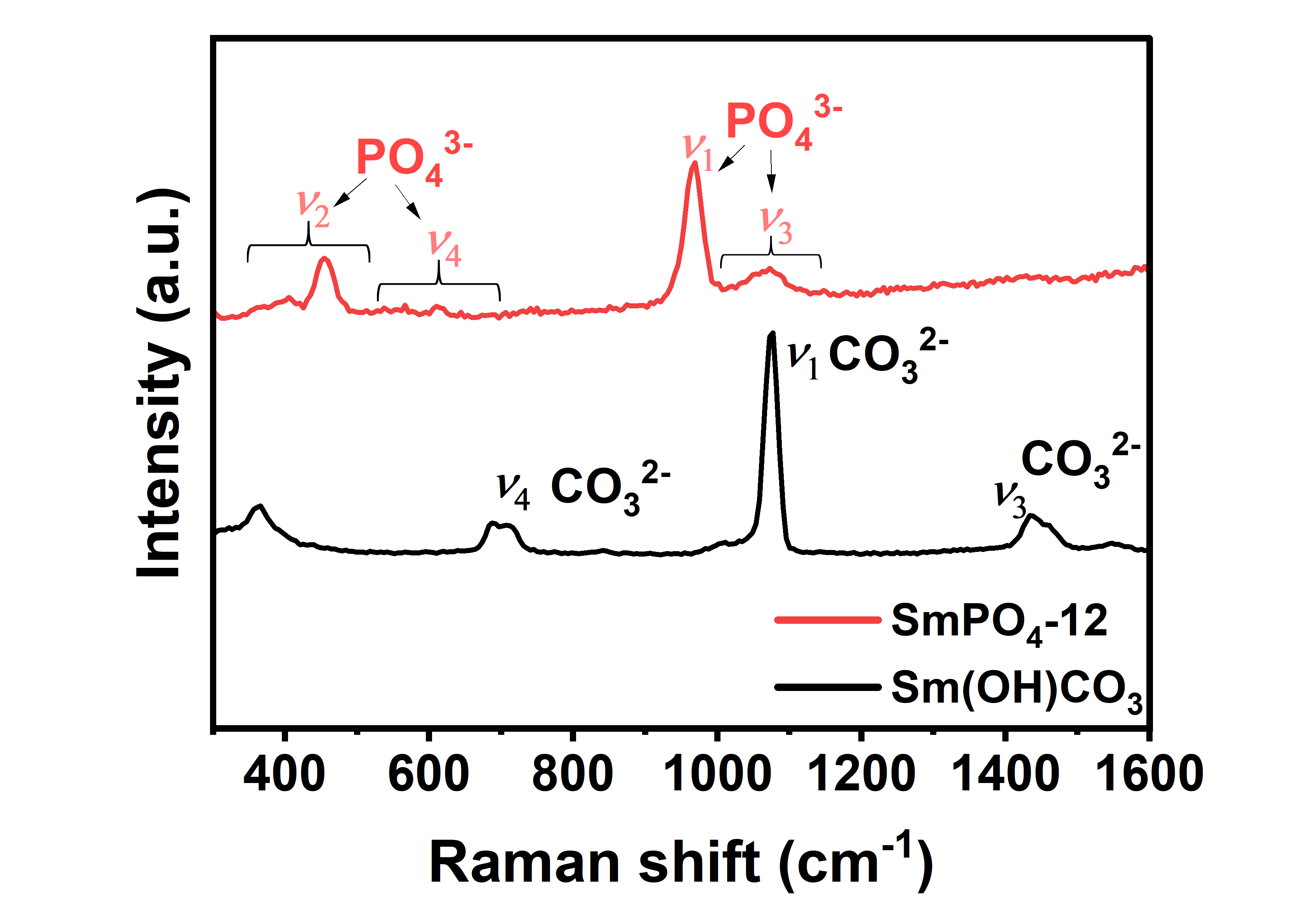


**Figure S3**. Raman spectra of Sm(OH)CO_3_ and SmPO_4_-12 powder.

**
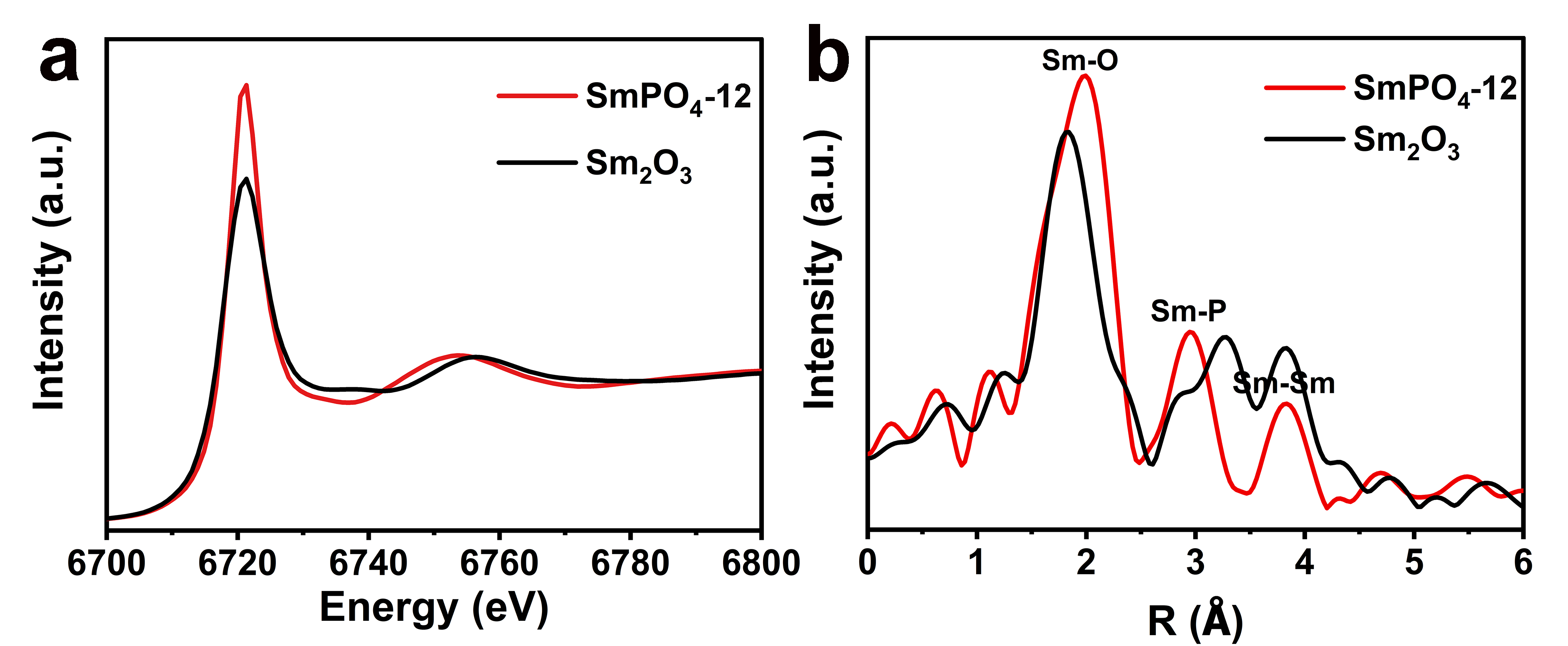
**

**Figure S4**. (a) Sm *L*_3_-edge XANES spectra for SmPO_4_-12 and Sm_2_O_3_. (b) Fourier-transformed EXAFS spectra of Sm for SmPO_4_-12 and Sm_2_O_3_.


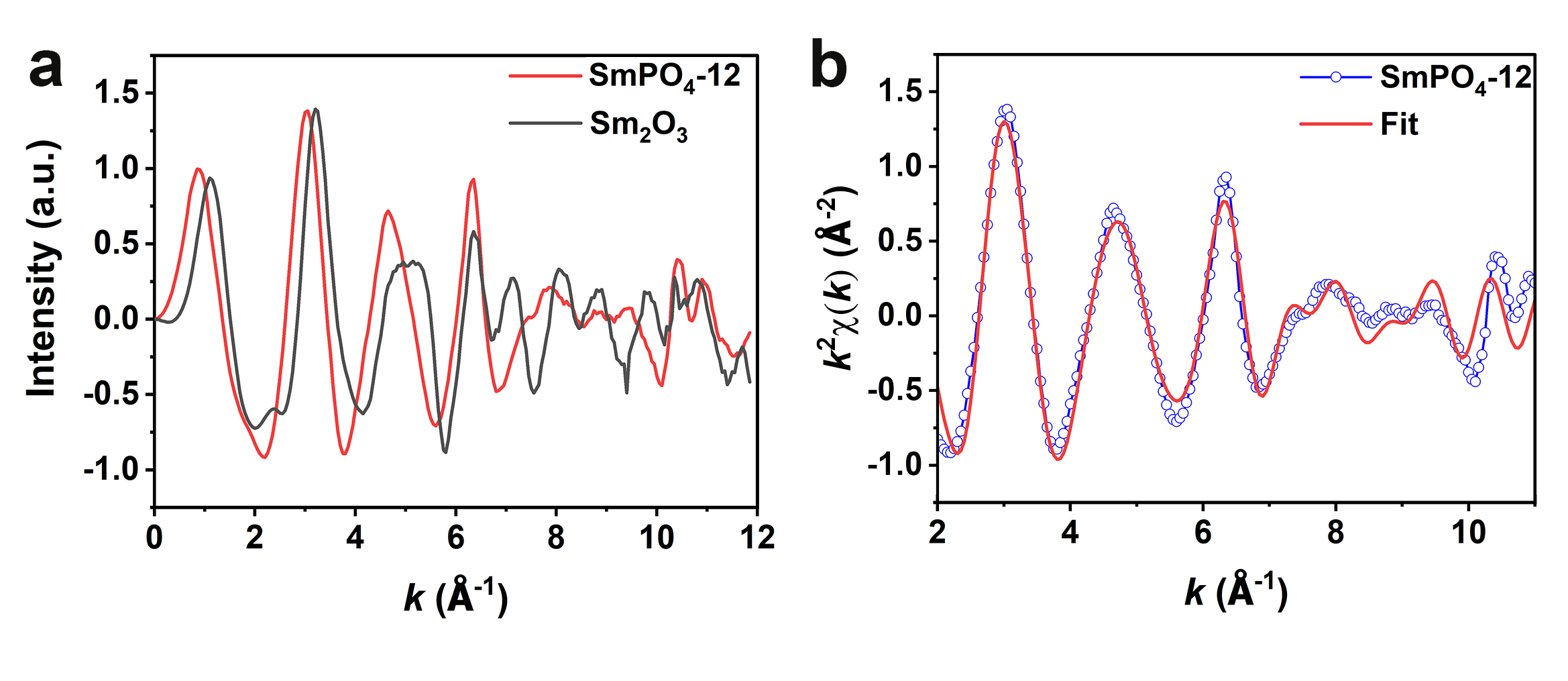


**Figure S5**. (a) The *k*-space plots SmPO_4_-12 and Sm_2_O_3_. (b) Fourier-transformed EXAFS spectra of Sm for SmPO_4_ in *k*-space.

**
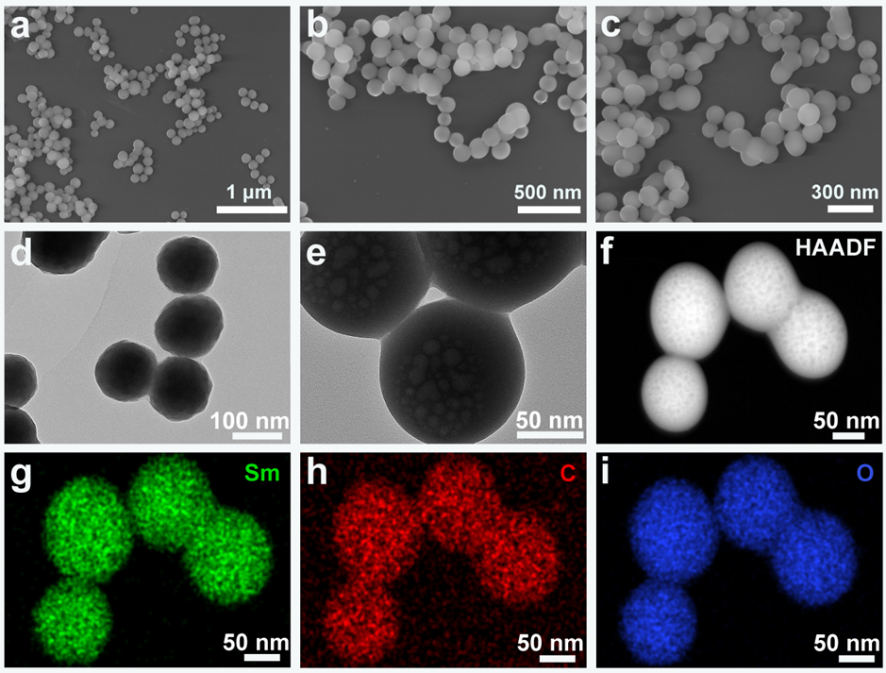
**

**Figure S6**. (a−c) The FESEM images and (d, e) TEM images of Sm(OH)CO_3_. (f) HAADF image of representative Sm(OH)CO_3_ and the corresponding EDX elemental mappings of (g) Sm, (h) C and (i) O.
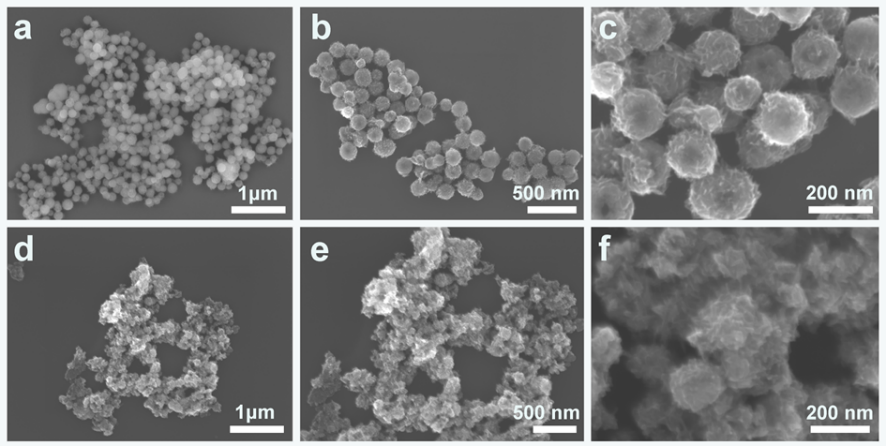


**Figure S7**. FESEM images of (a−c) SmPO_4_-1 and (d−f) SmPO_4_-24.


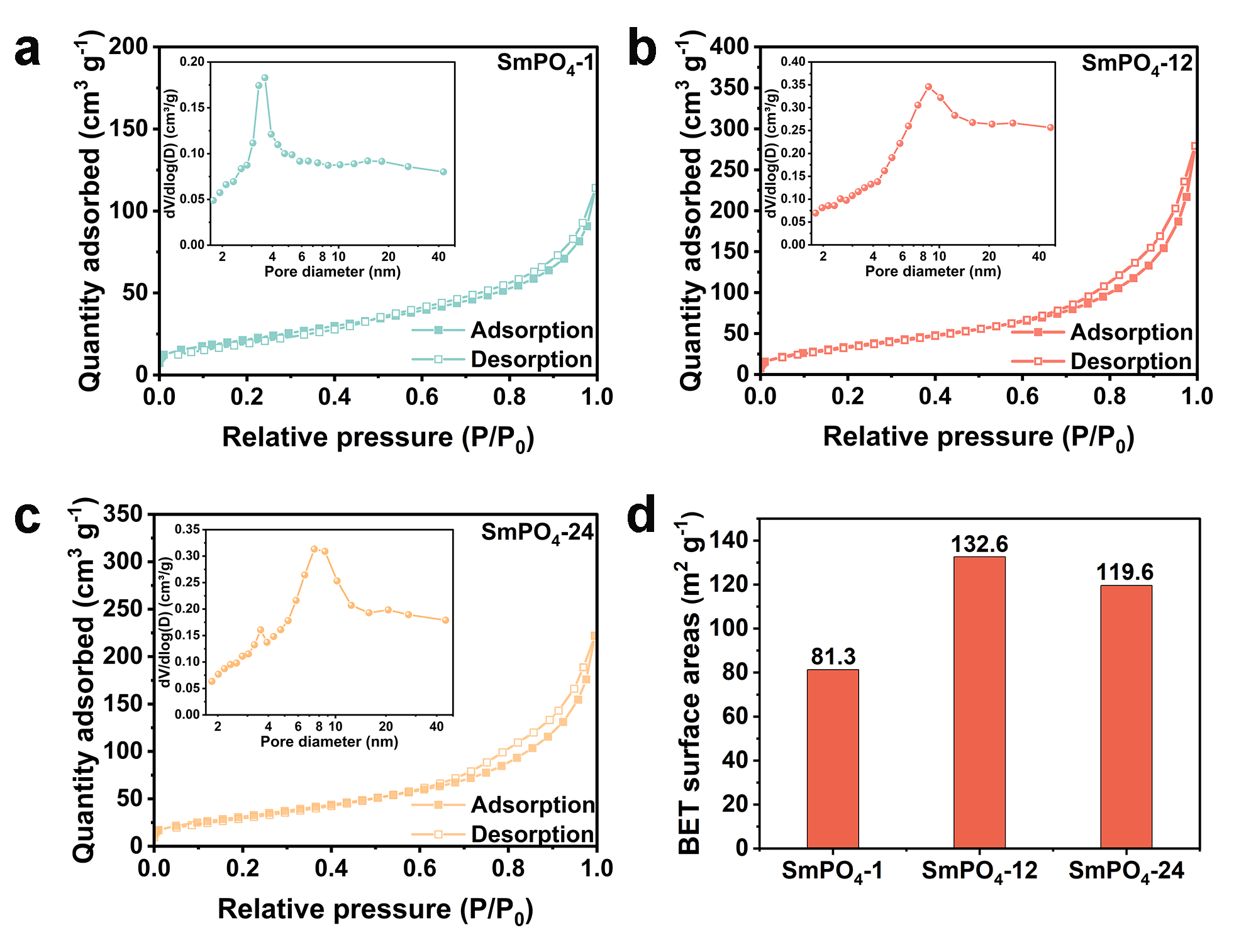


**Figure S8**. The N_2_ adsorption-desorption isotherms for (a) SmPO_4_-1, (b) SmPO_4_-12, and (c) SmPO_4_-24 are presented in Figures a, b, and c, respectively. The corresponding pore size distributions for SmPO_4_-1, SmPO_4_-12, and SmPO_4_-24 are given as insets in these figures. (d) The corresponding BET surface areas were measured as 81.3 m^2^ g^−1^, 132.6 m^2^ g^−1^, and 119.6 m^2^ g^−1^, respectively.

**
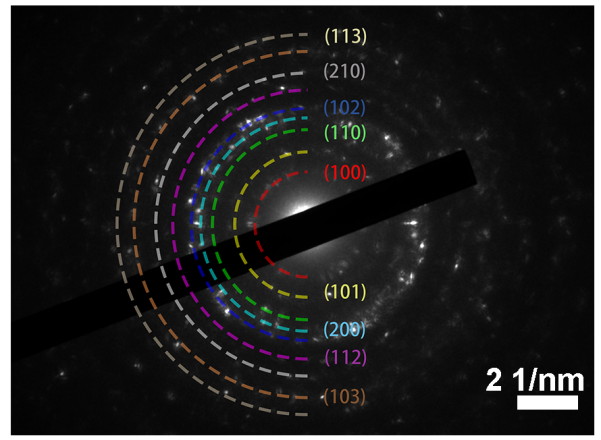
**

**Figure S9**. The corresponding SAED pattern of representative SmPO_4_-12 nanosphere.


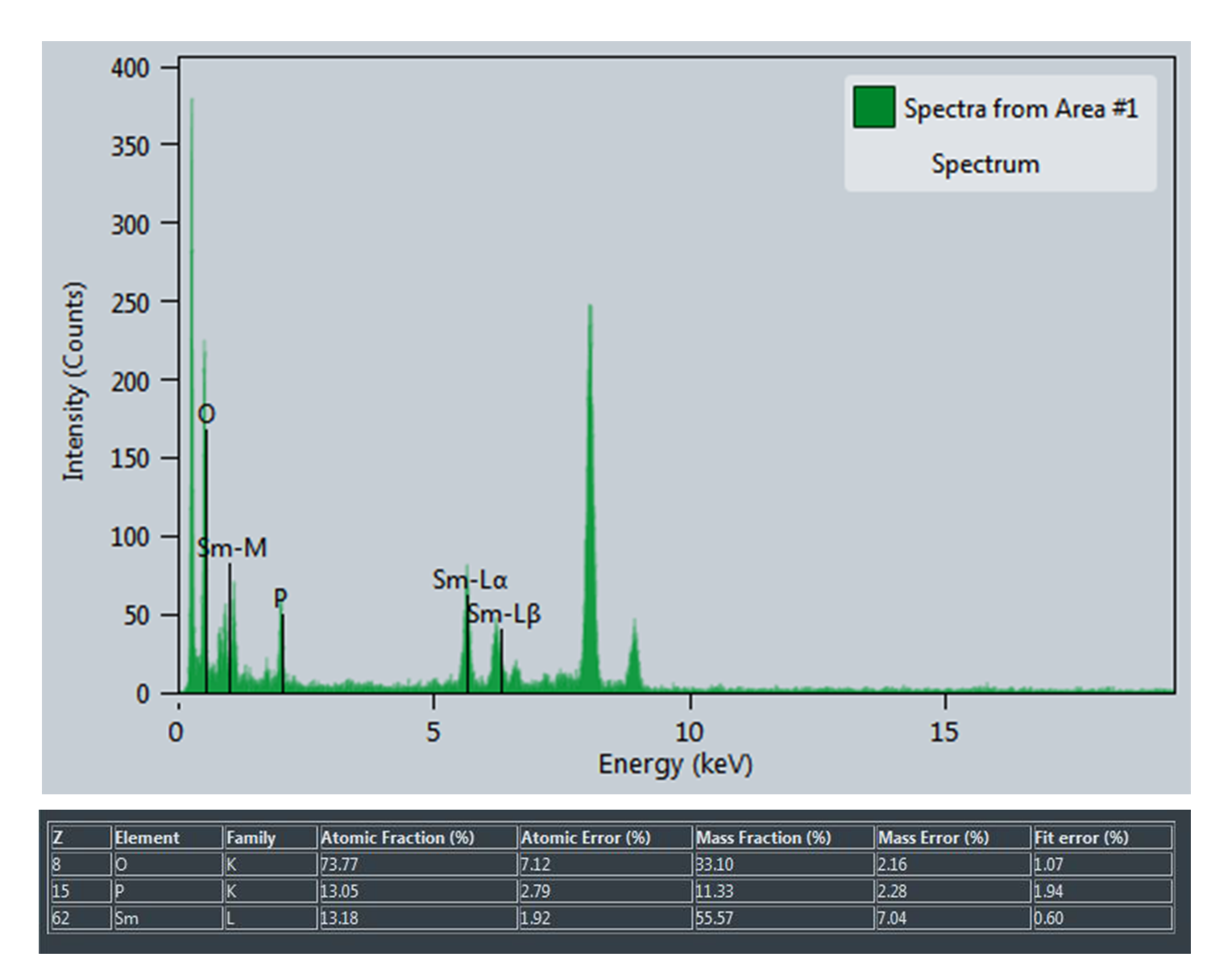


**Figure S10**. TEM-EDX mapping spectra of pristine SmPO_4_-12.


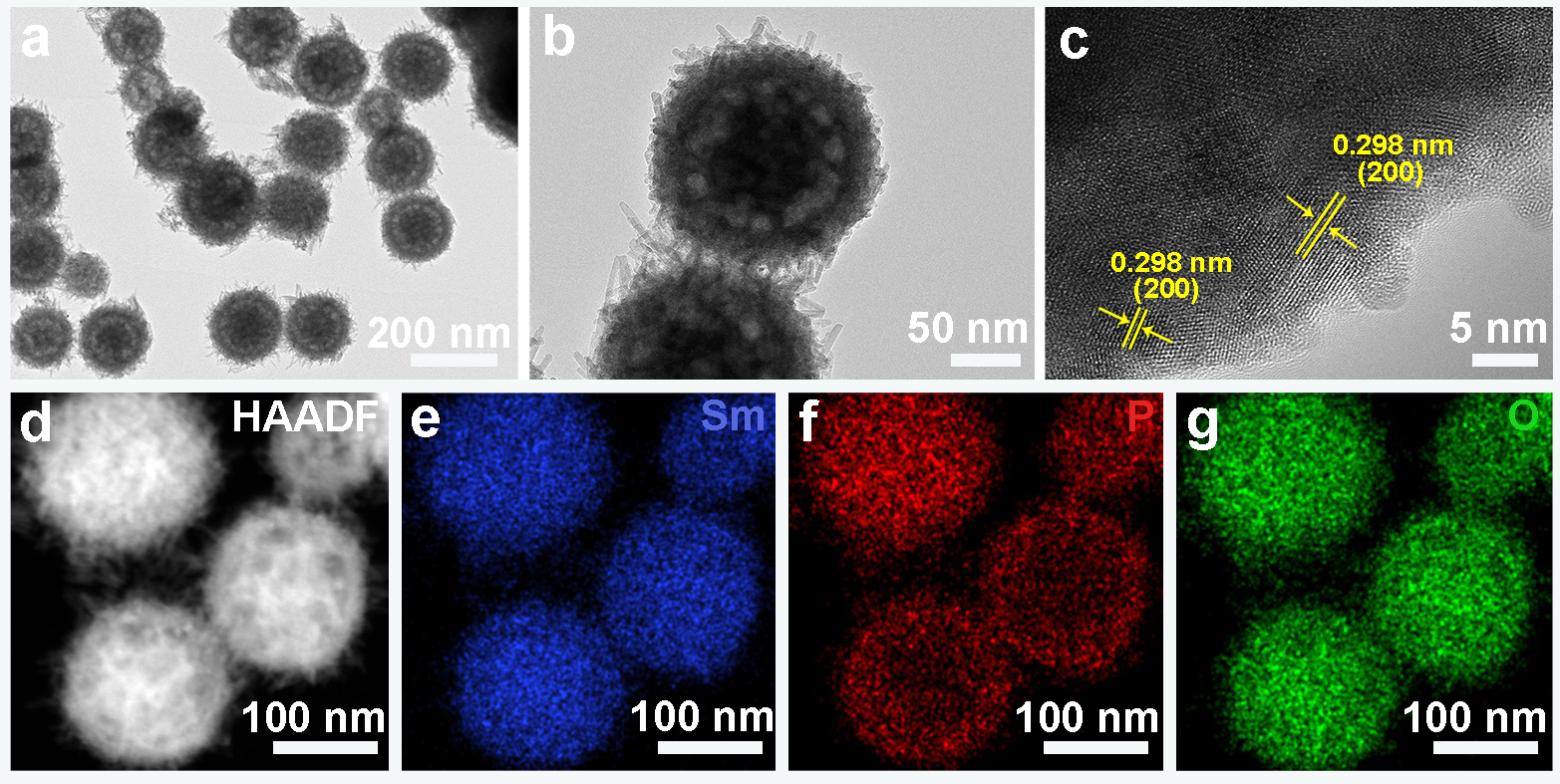


**Figure S11**. (a, b) The FESEM and (c) high-resolution TEM images of SmPO_4_-1. (d) HAADF image of representative SmPO_4_-1 and the corresponding EDX elemental mapping of (e) Sm, (f) P and (g) O species.

**
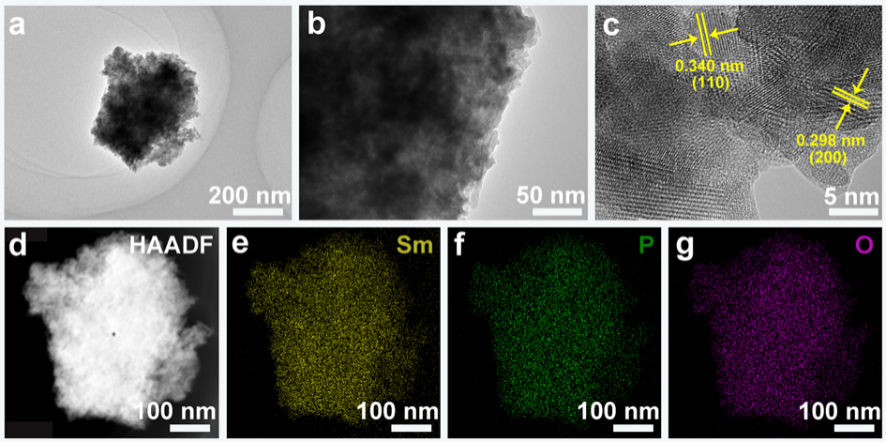
**

**Figure S12**. (a, b) The FESEM and (c) high-resolution TEM images of SmPO_4_-24. (d) HAADF image of representative SmPO_4_-24 and the corresponding EDX elemental mapping of (e) Sm, (f) P and (g) O species.


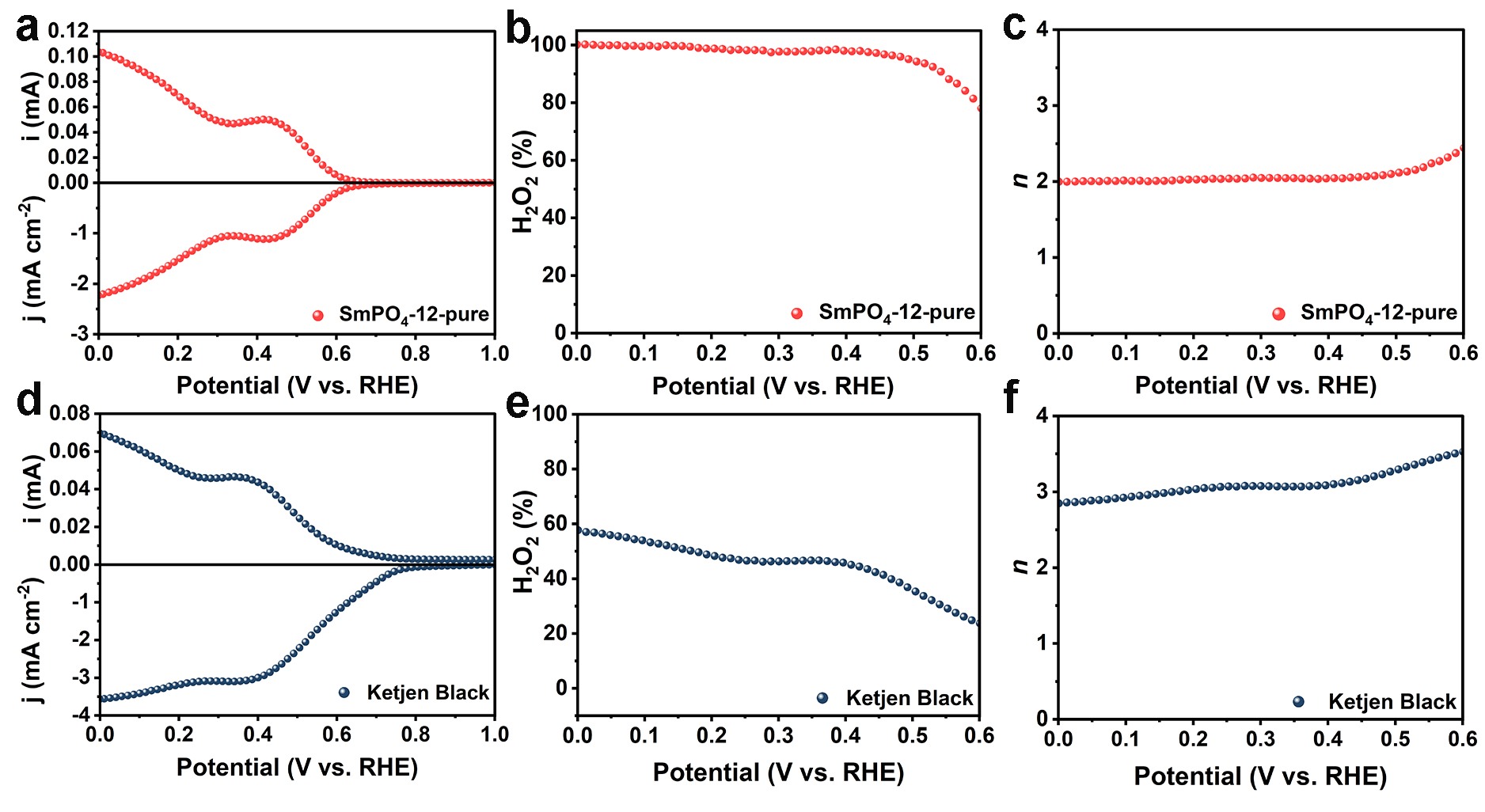


**Figure S13**. (a) LSV curves of pure SmPO_4_-12 (without utilizing Ketjen Black) recorded at 1600 rpm with a scan rate of 10 mV s^−1^ (bottom part), together with the corresponding H_2_O_2_ current on the ring electrode (upper part) in O_2_-saturated 0.1 M KOH. (b) Selectivity of H_2_O_2_ and (c) calculated electron transfer number (n) within the potential sweep.(d) LSV curves of Ketjen Black recorded at 1600 rpm with a scan rate of 10 mV s^−1^ (bottom part), together with the corresponding H_2_O_2_ current on the ring electrode (upper part) in O_2_-saturated 0.1 M KOH. (e) Selectivity of H_2_O_2_ and (f) calculated electron transfer number (n) within the potential sweep.


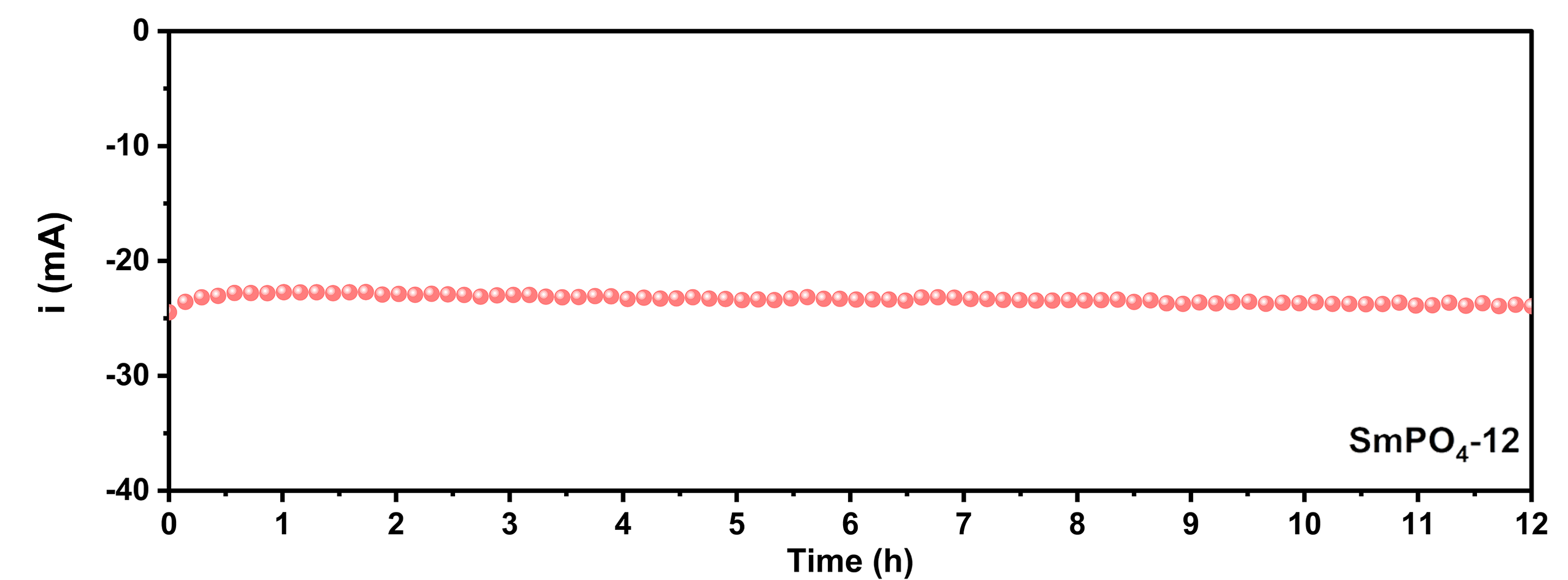


**Figure S14**. Chronoamperometry test of SmPO_4_-12 at high current density in H-cell (0.48 V *vs.* RHE) in O_2_-saturated 1 M KOH.


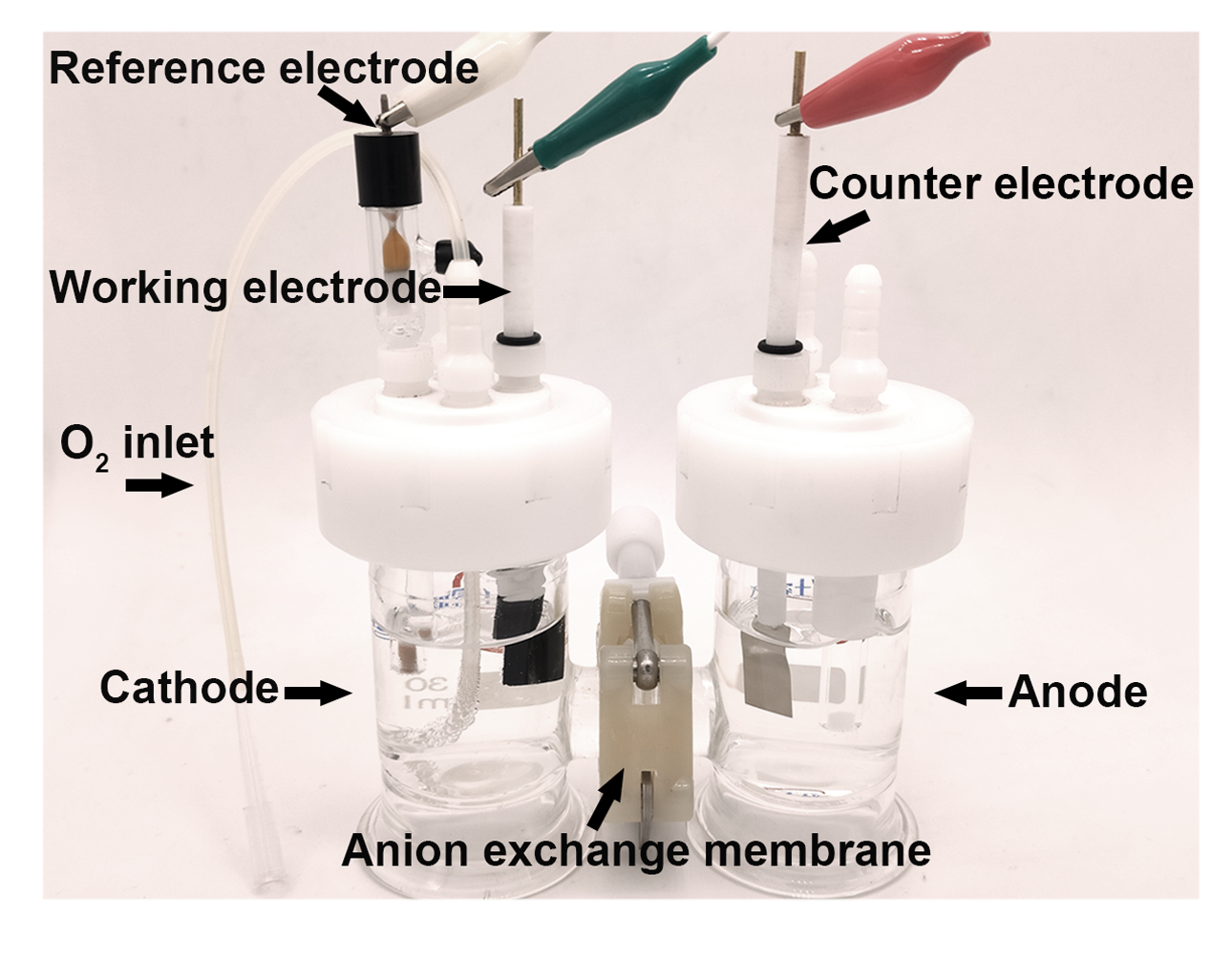


**Figure S15**. Digital image for the H-cell configuration by using SmPO_4_-12 cast on carbon paper as electrode.


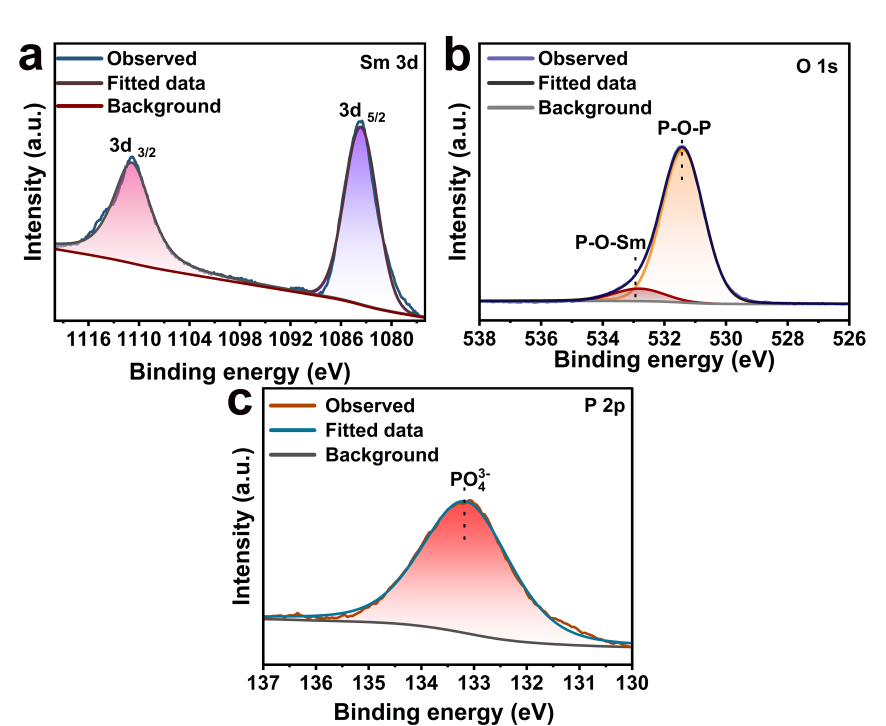


**Figure S16**. High-resolution XPS spectra of (a) Sm 3d, (b) O 1s, (c) P 2p in SmPO_4_-12 after ORR cycling.


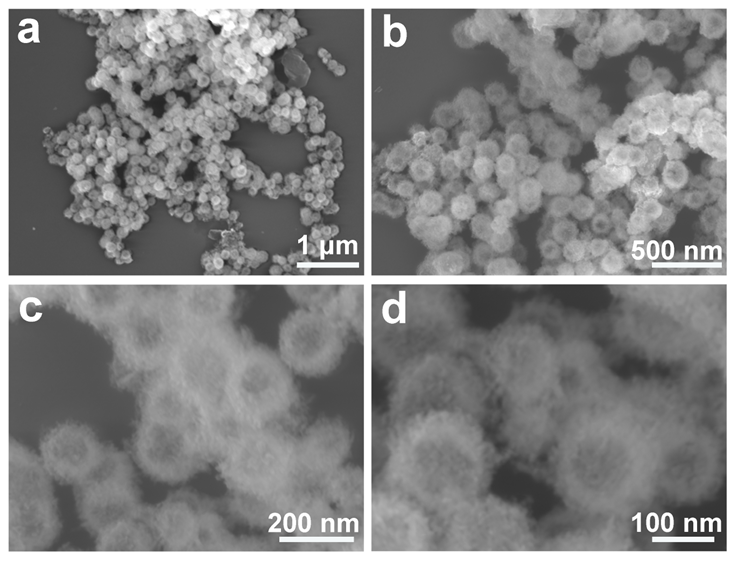


**Figure S17**. (a−d) FESEM images of SmPO_4_-12 after ORR cycling.


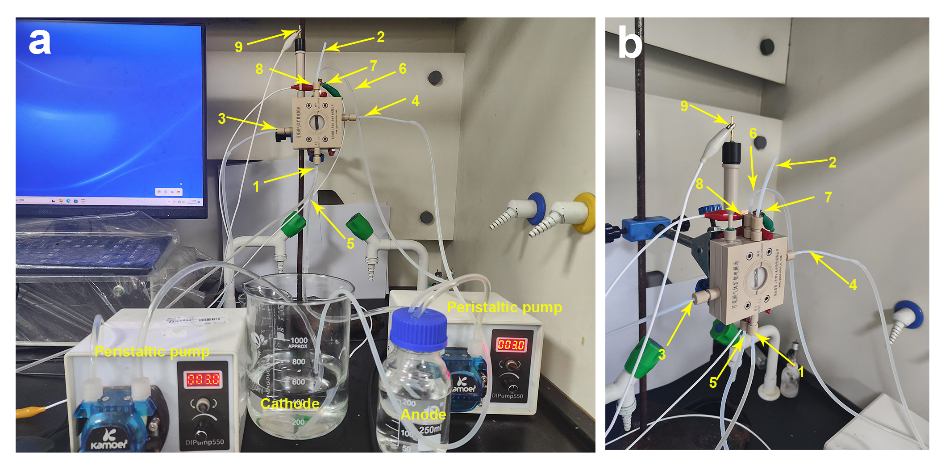


**Figure S18**. The optical image of the typical flow cell: 1. O_2_ flow inlet; 2. O_2_ flow outlet; 3. Cathode electrolyte inlet; 4. Cathode electrolyte outlet; 5. Anode electrolyte inlet; 6. Anode electrolyte outlet; 7. Working electrode (where catalysts were deposited); 8. Counter electrode (Ni foam); 9. Hg/HgO or Hg/HgCl_2_ reference electrode. Note that when conducting the bulk electrolysis in a three-electrode configuration, the three clips of the electrochemical workstation were connected to the working, reference, and counter electrodes, respectively. Ni foam that herein served as the anode of the single full cell, and the working electrode deposited with the studied catalyst acted as the cathode.


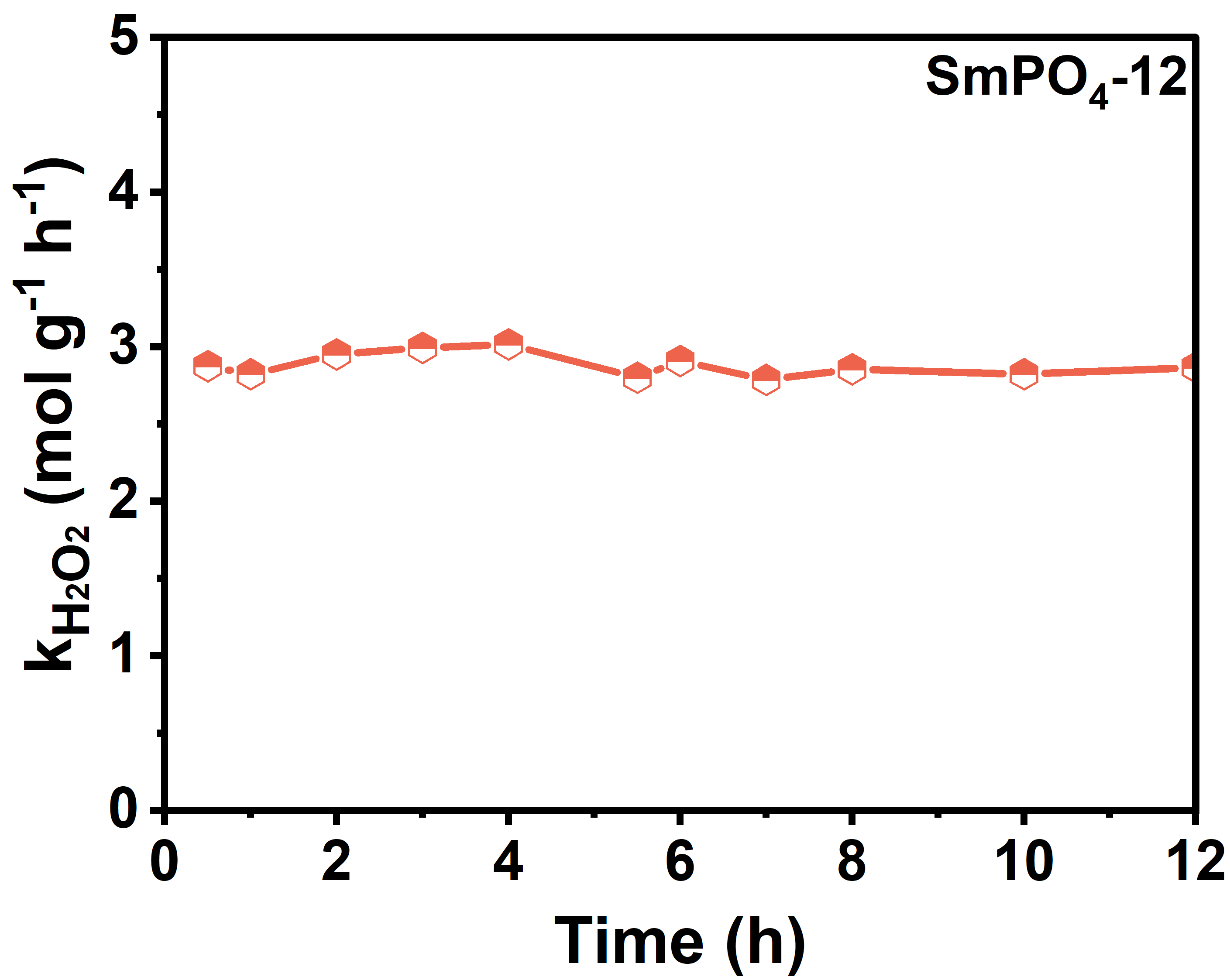


**Figure S19**. The number of moles of H_2_O_2_ produced by SmPO_4_-12 with the electrolytic time and the mass of the catalyst under the O_2_ condition in 0.1 M KOH in the gas diffusion electrode.


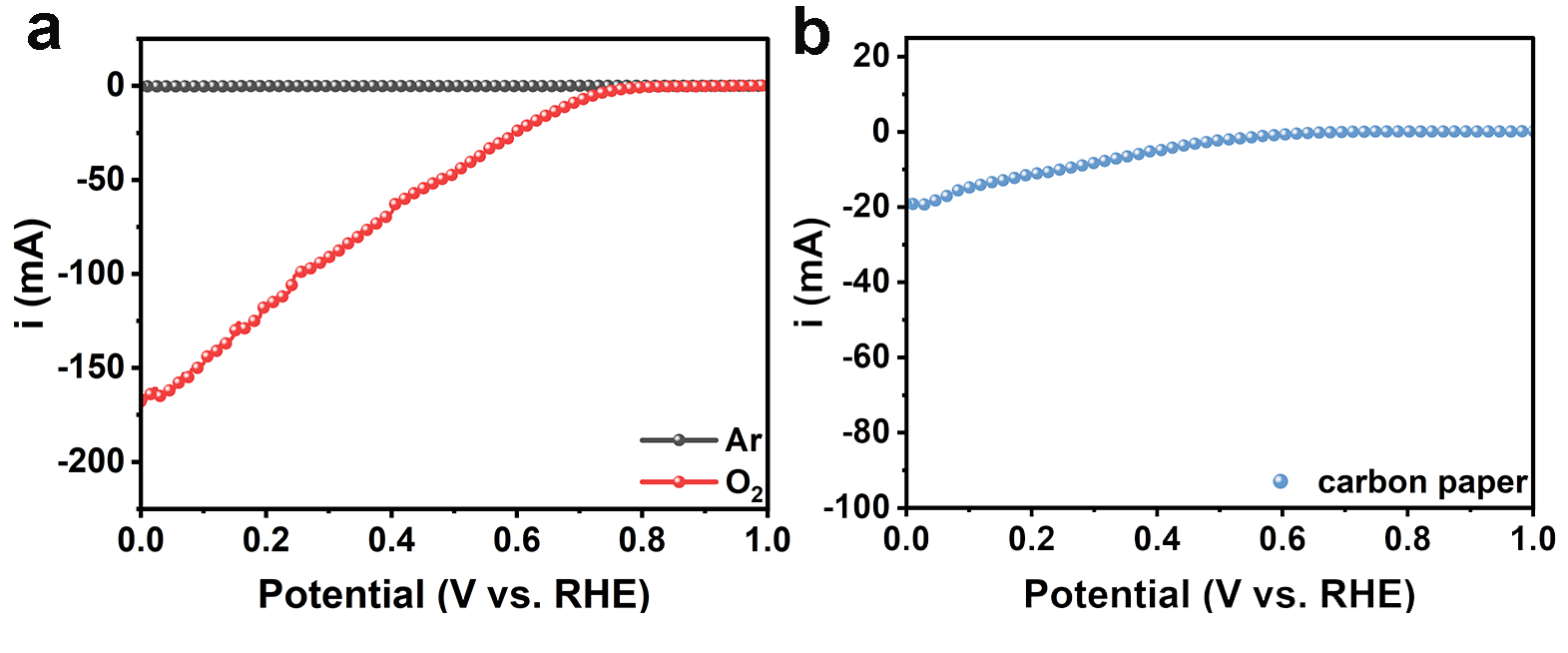


**Figure S20**. (a) LSV curves of 0.5 mg cm^−2^ SmPO_4_-12 catalyst supported on 0.5×2 cm^2^ carbon paper under oxygen and argon in flow cell. (b) LSV curve of no catalyst supported on 0.5×2 cm^2^ carbon paper under oxygen in flow cell.


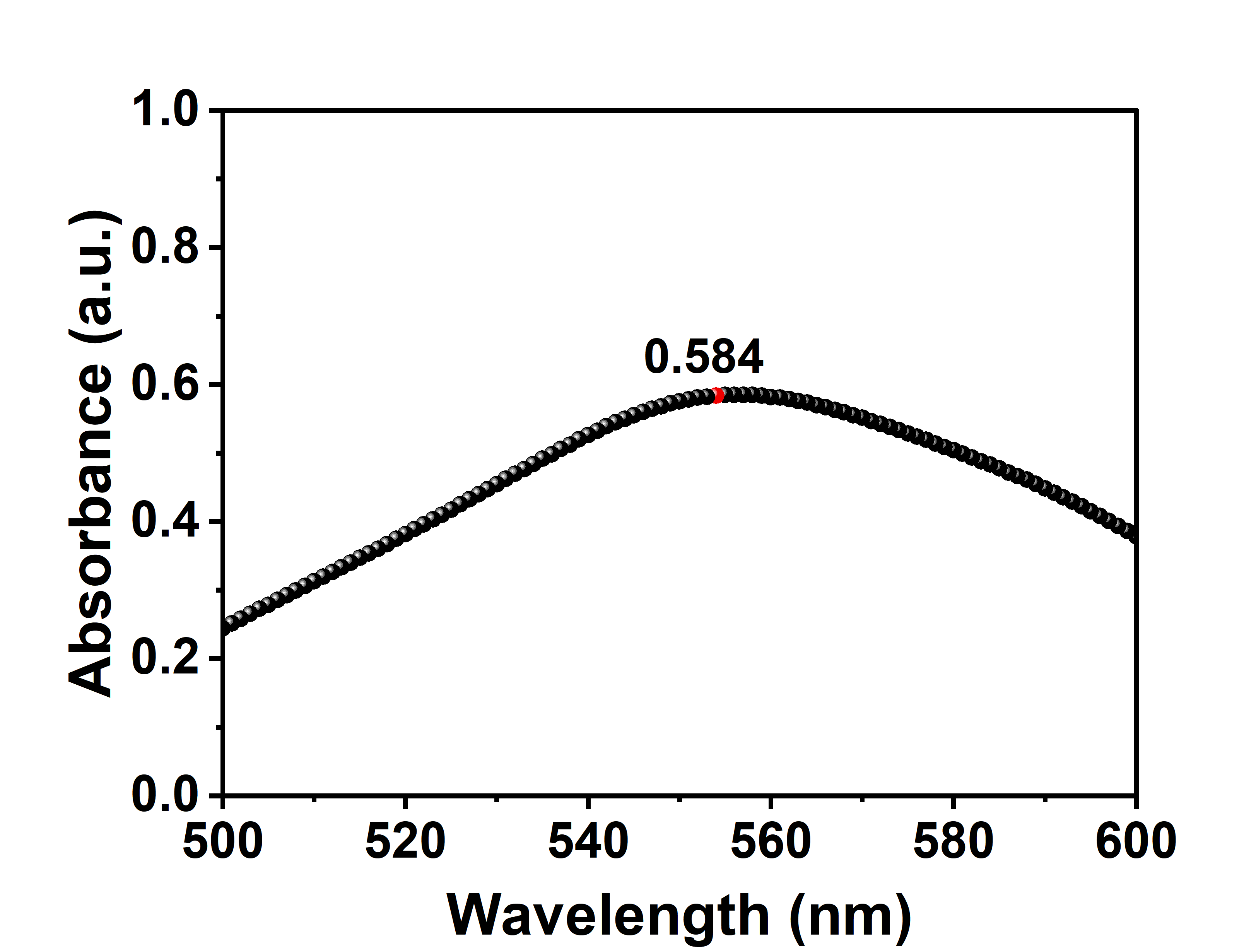


**Figure S21**. The UV absorption spectrum of the catholyte after a 1800s CP test in a flow cell under saturated oxygen conditions with 0.1 M KOH.


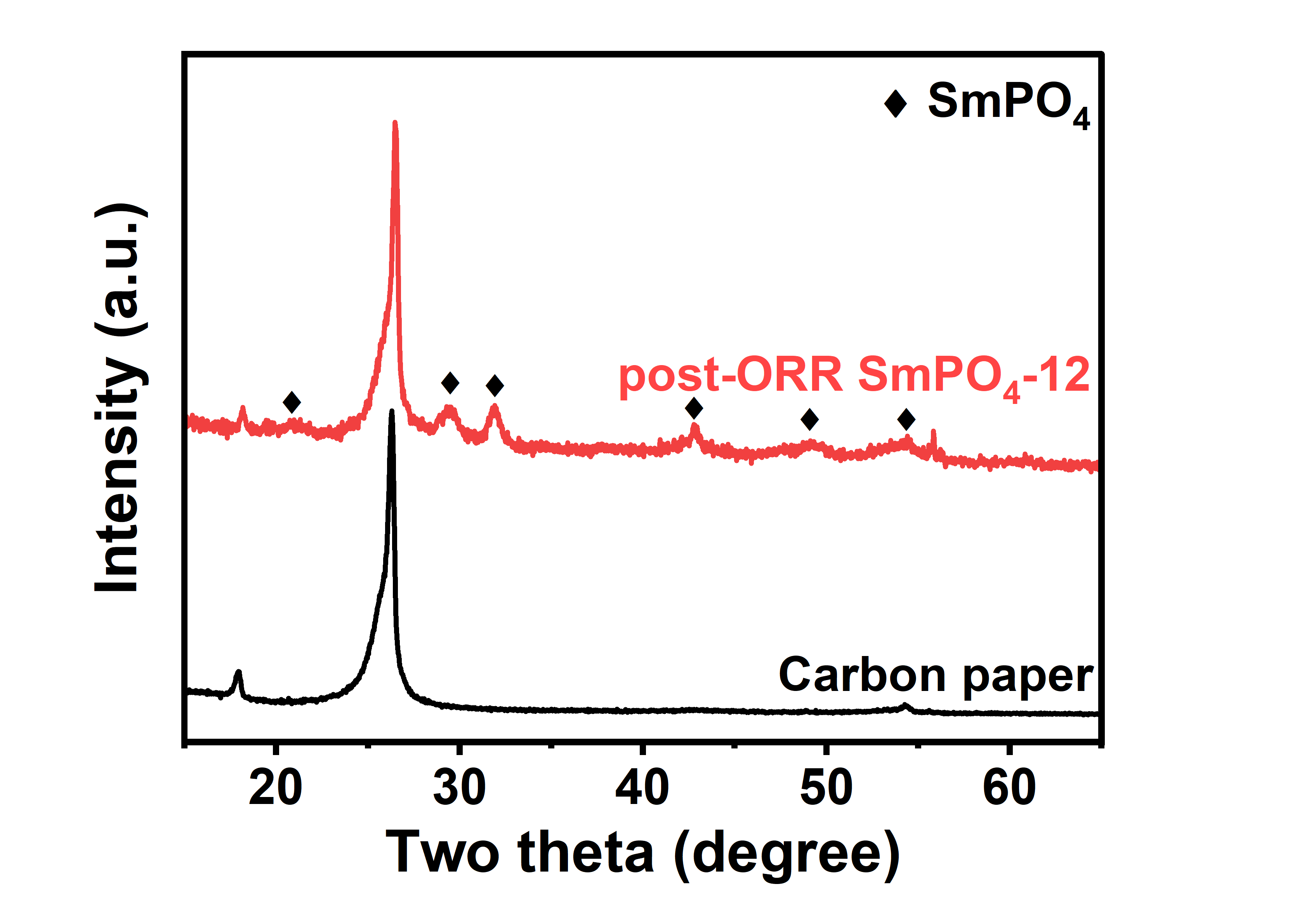


**Figure S22**. The XRD pattern of SmPO_4_-12 after ORR cycling.

**
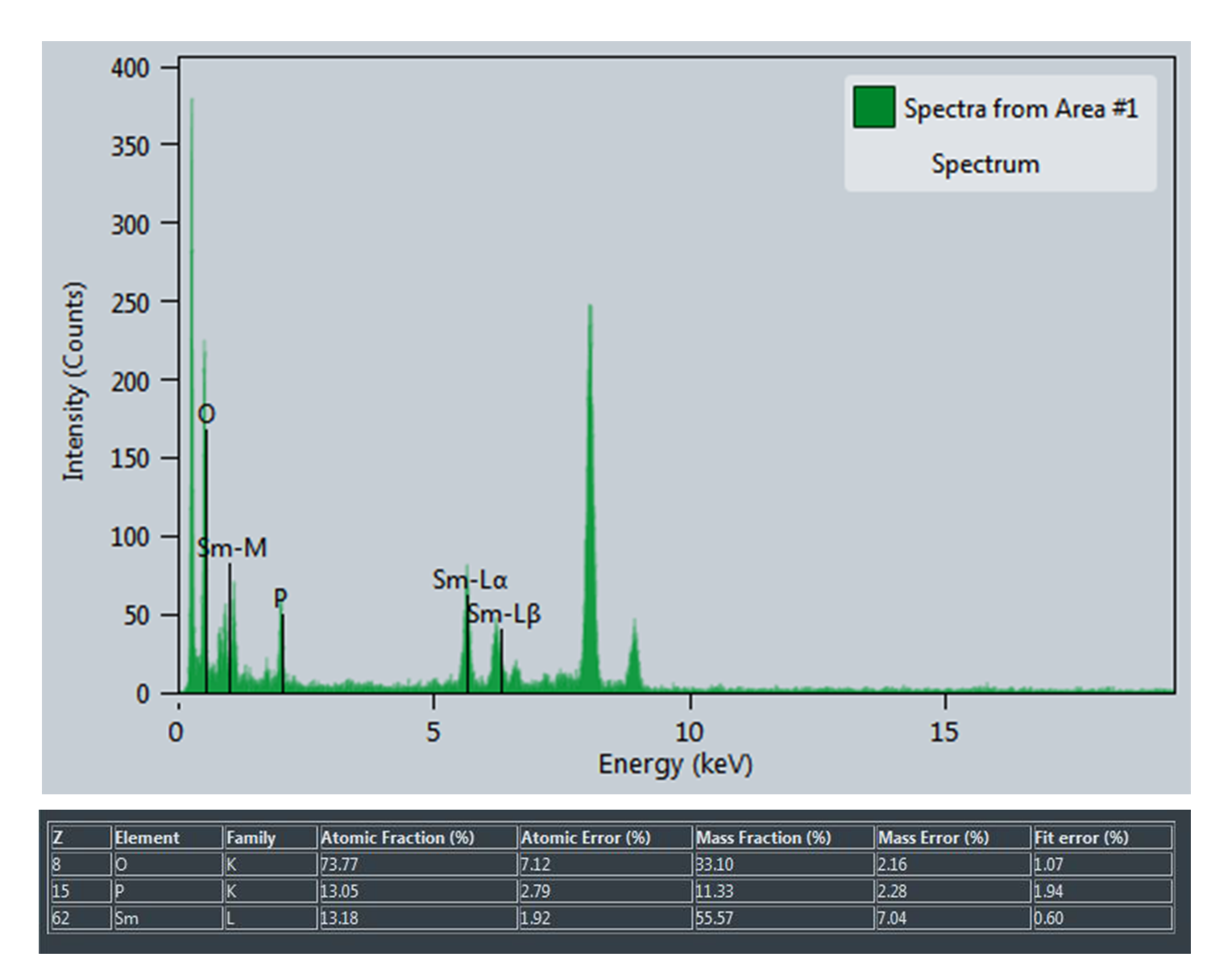
**

**Figure** **S23**. TEM-EDX spectra of SmPO_4_-12 after ORR cycling.


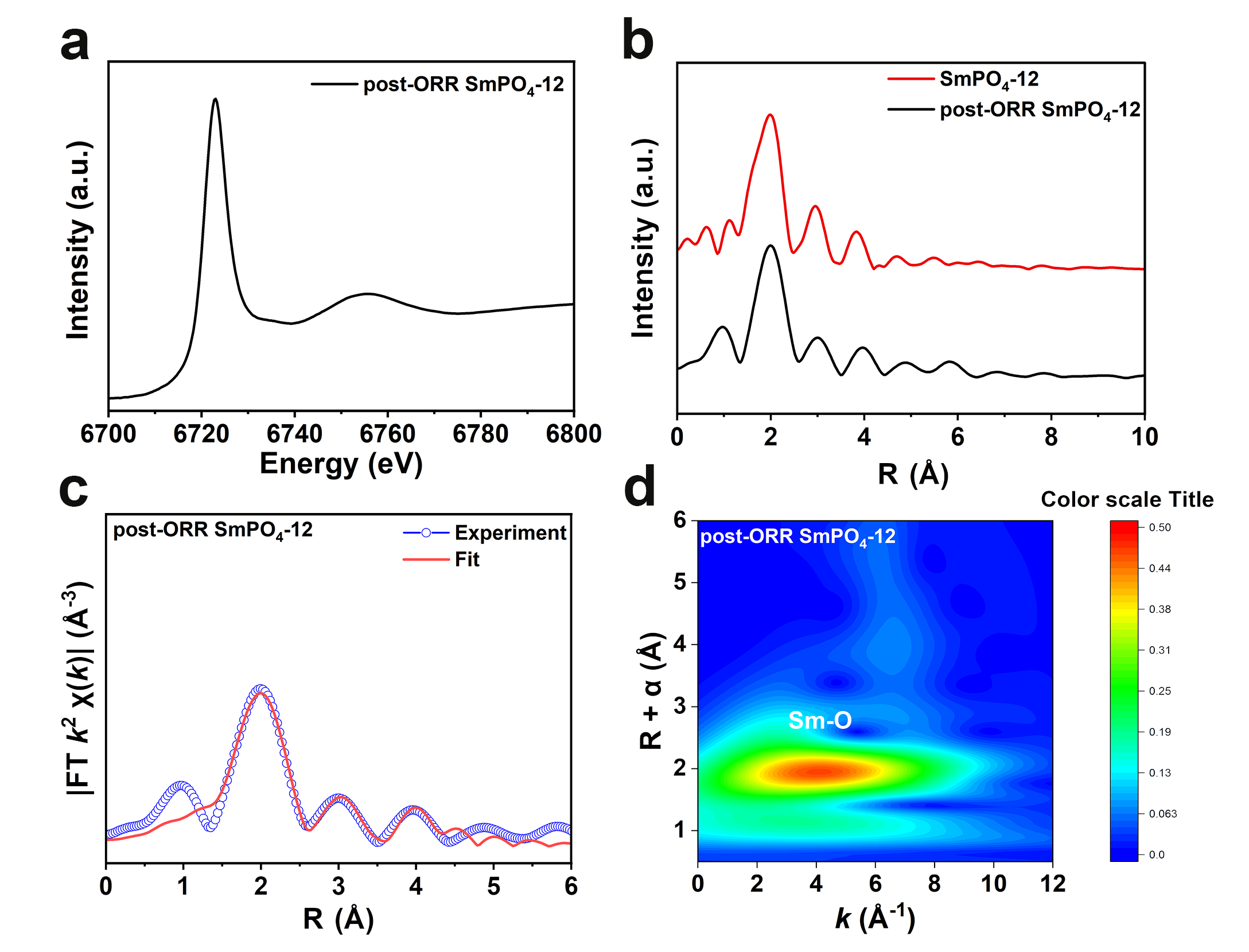


**Figure S24**. Sm *L*_3_-edge (a) XANES for post-ORR SmPO_4_-12 and (b) Fourier transformed (FT)-EXAFS spectra for fresh and post-ORR SmPO_4_-12. (c) Sm *L*_3_-edge FT-EXAFS spectra of post-ORR SmPO_4_-12 fitted in R space. (d) Wavelet transform (WT) of Sm *L*_3_-edge EXAFS for post-ORR SmPO_4_-12.

**
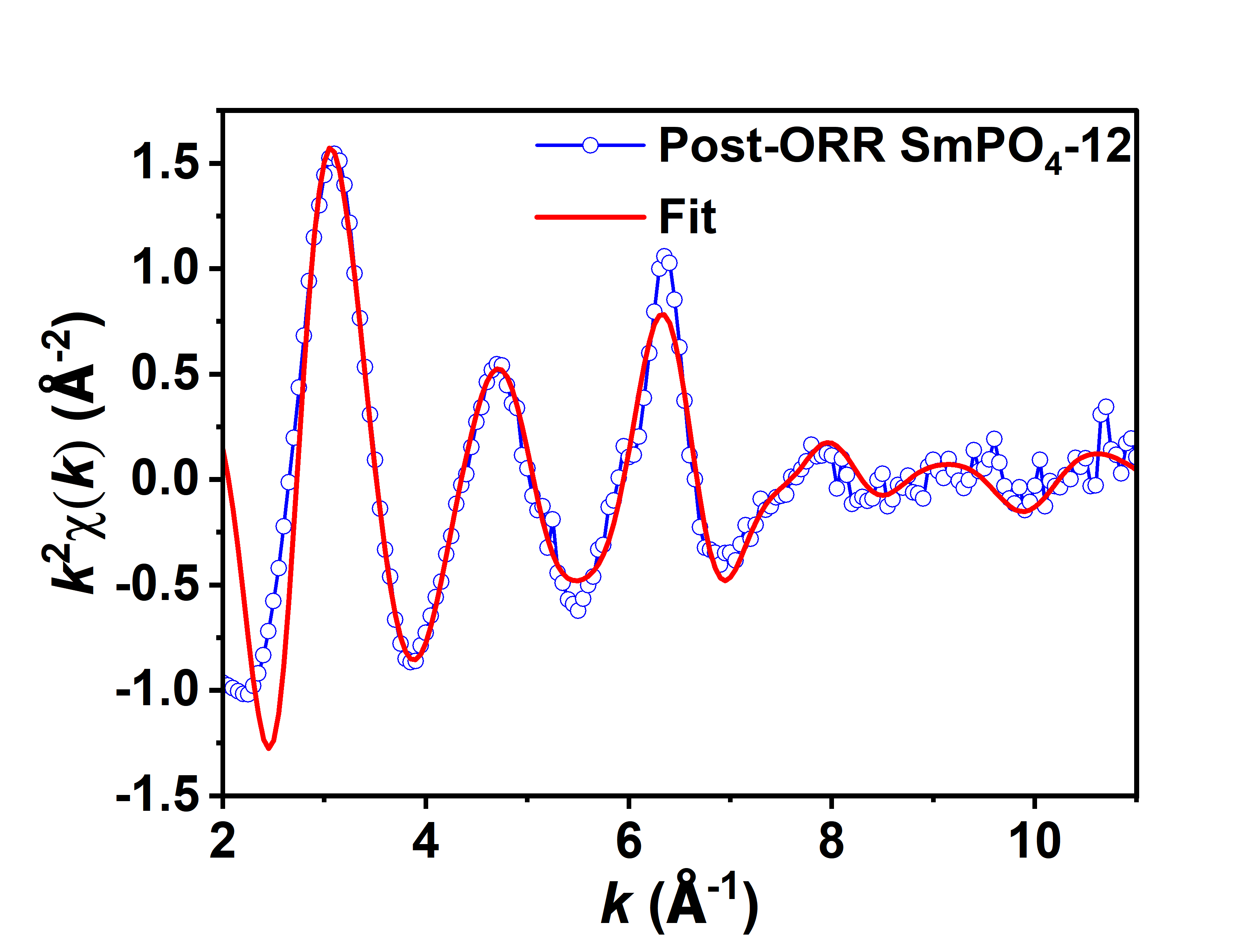
**

**Figure S25.**The EXAFS (including the fitted data) in *k*-space for the SmPO_4_-12 after ORR electrolysis.


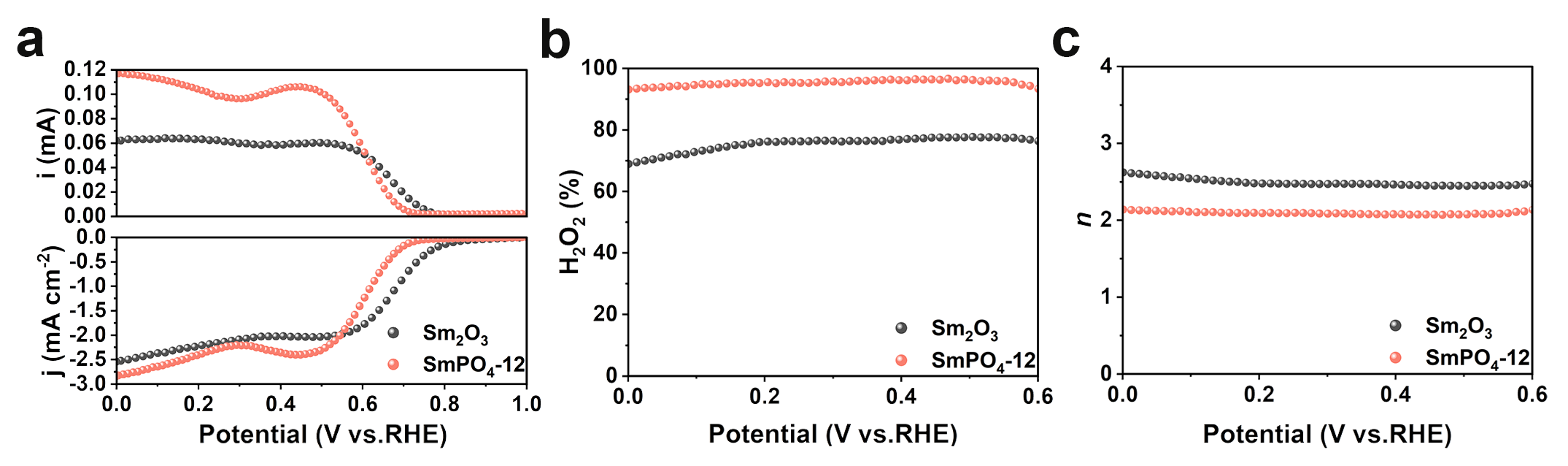


**Figure S26**. (a) LSV curves of Sm_2_O_3_ and SmPO_4_-12 recorded at 1600 rpm with a scan rate of 10 mV s^−1^ (bottom part), together with the corresponding H_2_O_2_ current on the ring electrode (upper part) in O_2_-saturated 0.1 M KOH. (b) Selectivity of H_2_O_2_ and (c) calculated electron transfer number (*n*) within the potential sweep.


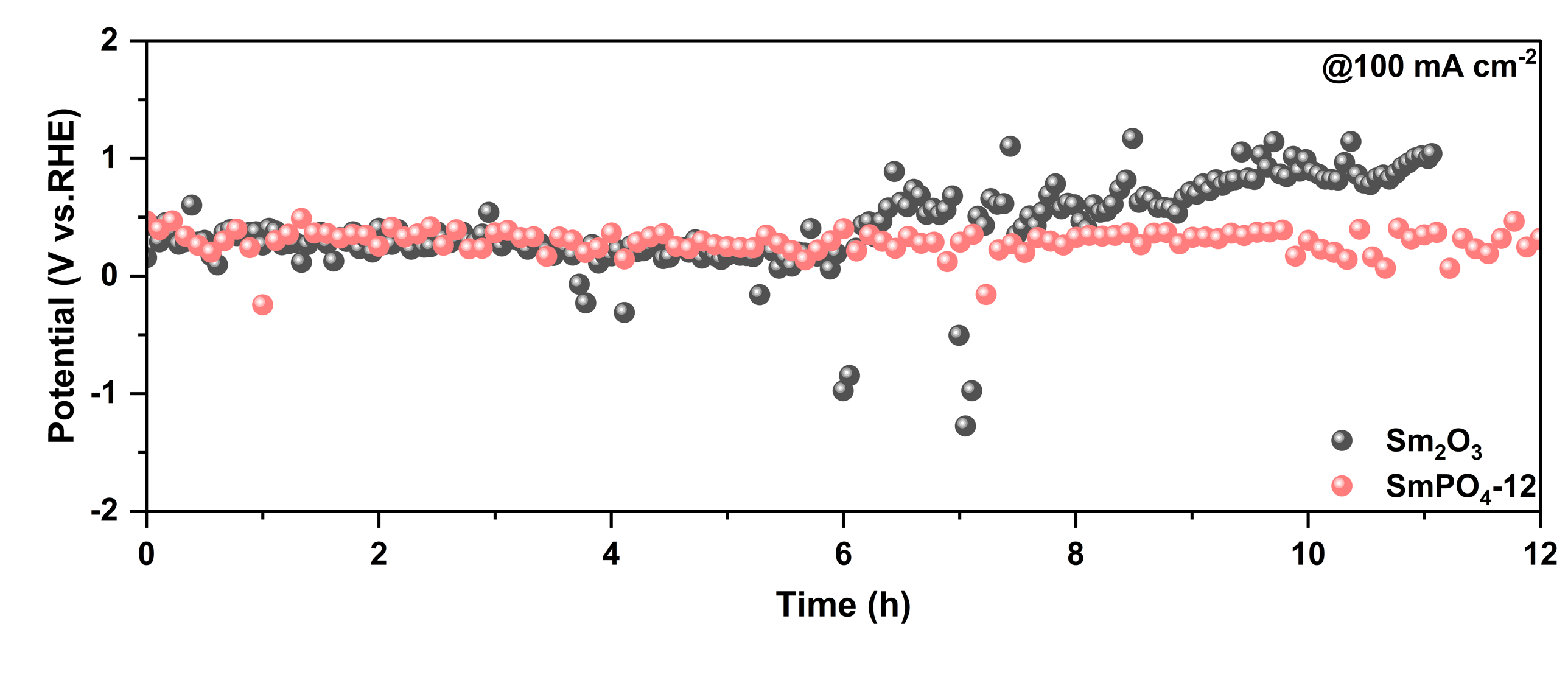


**Figure S27**. Time-voltage curve under O_2_ conditions in 0.1 M KOH for Sm_2_O_3_ and SmPO_4_-12 in a gas diffusion electrode (100 mA cm^−2^).

**
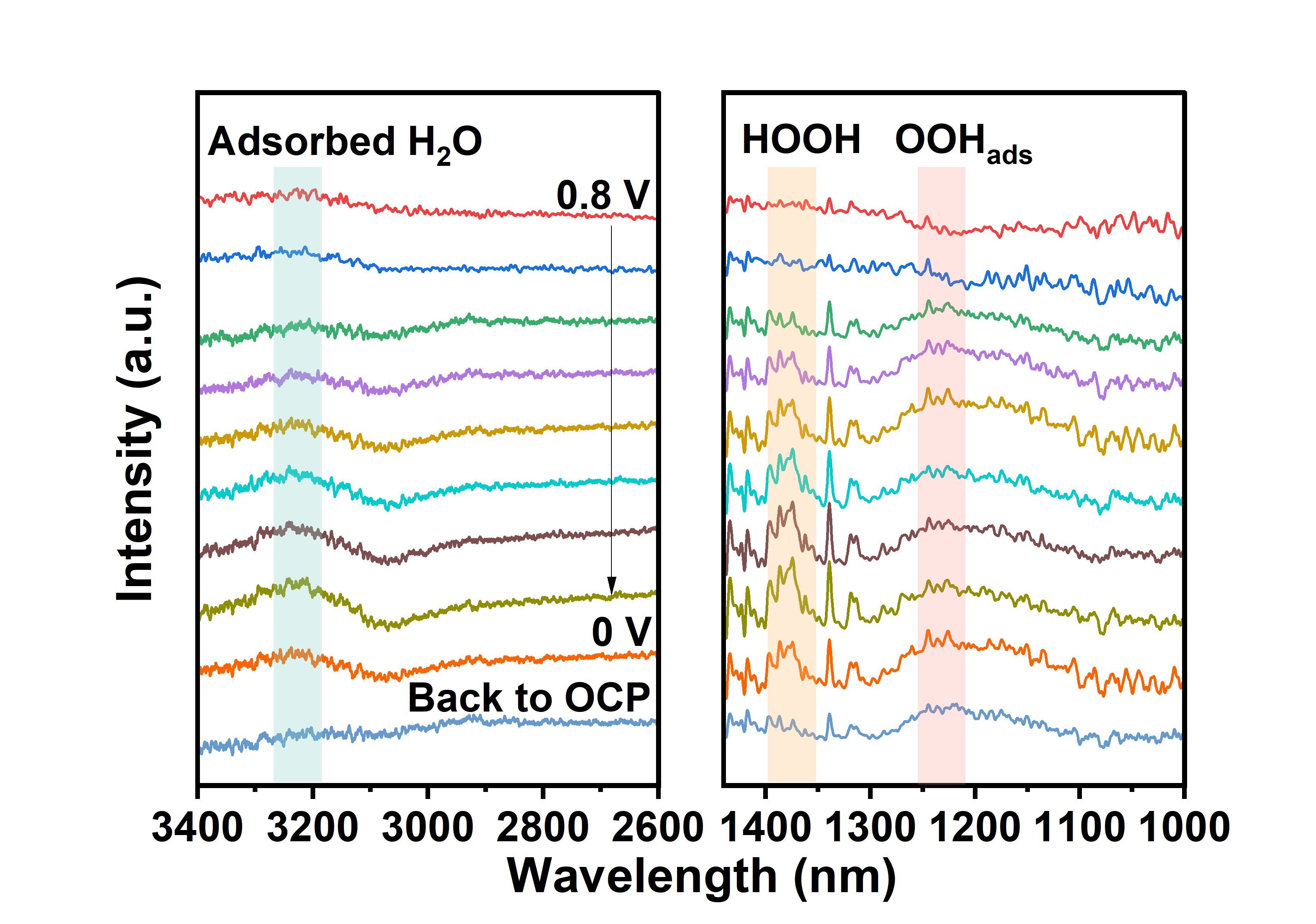
**

**Figure S28**. In situ ATR-IR spectra under applied potentials of SmPO_4_ in O_2_-saturated 0.1 M KOH within a voltage window 0.8~0 V *vs*. RHE as well as the ex situ ones under OCP. The spectral bands in the frequency range of 1300 to 1350 cm^−1^ can be attributed to the vibration of fluorocarbons from Nafion during ORR catalysis^[6]^.

**
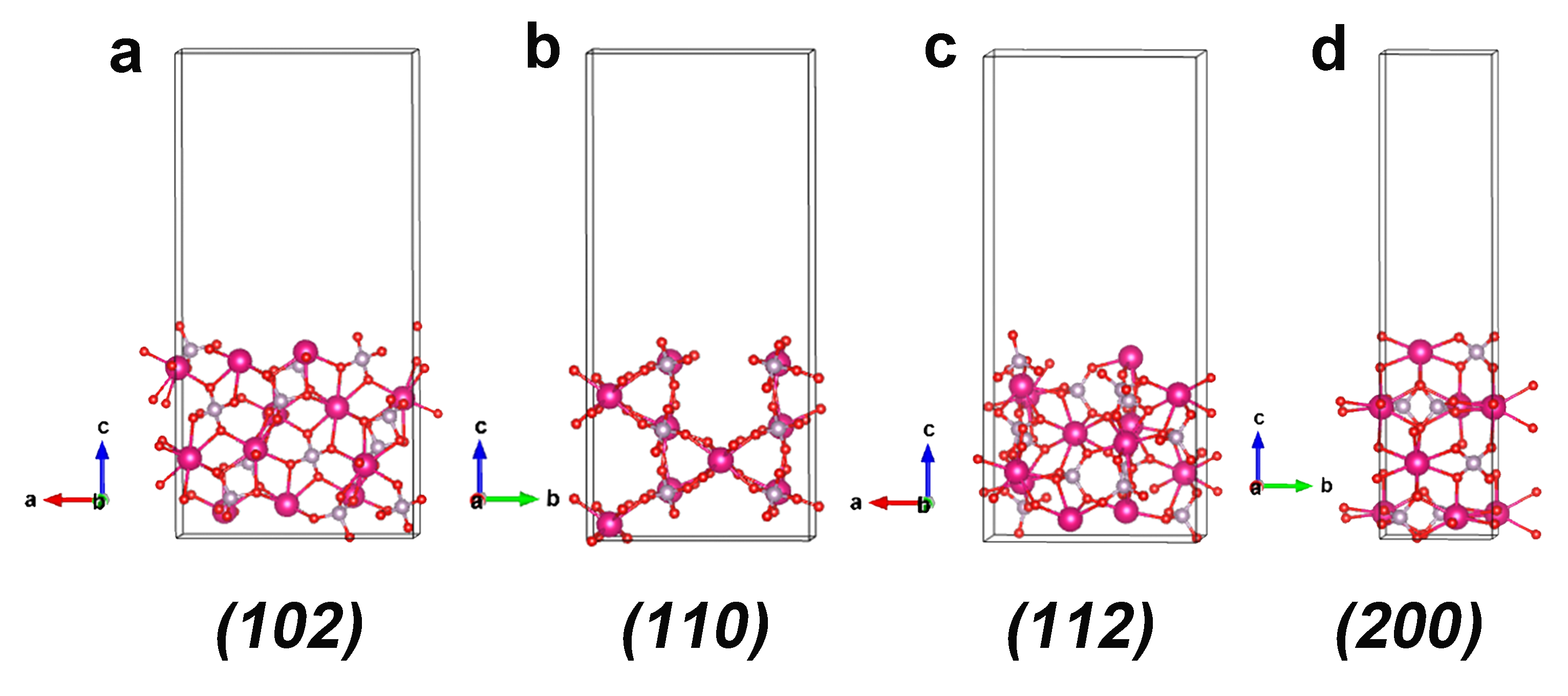
**

**Figure S29**. The DFT calculation models of (a) (102), (b) (110), (c) (112), (d) (200) facets of SmPO_4_, where the grey, red, and rose red sphere represents the P, O, and Sm atom, respectively

.

**
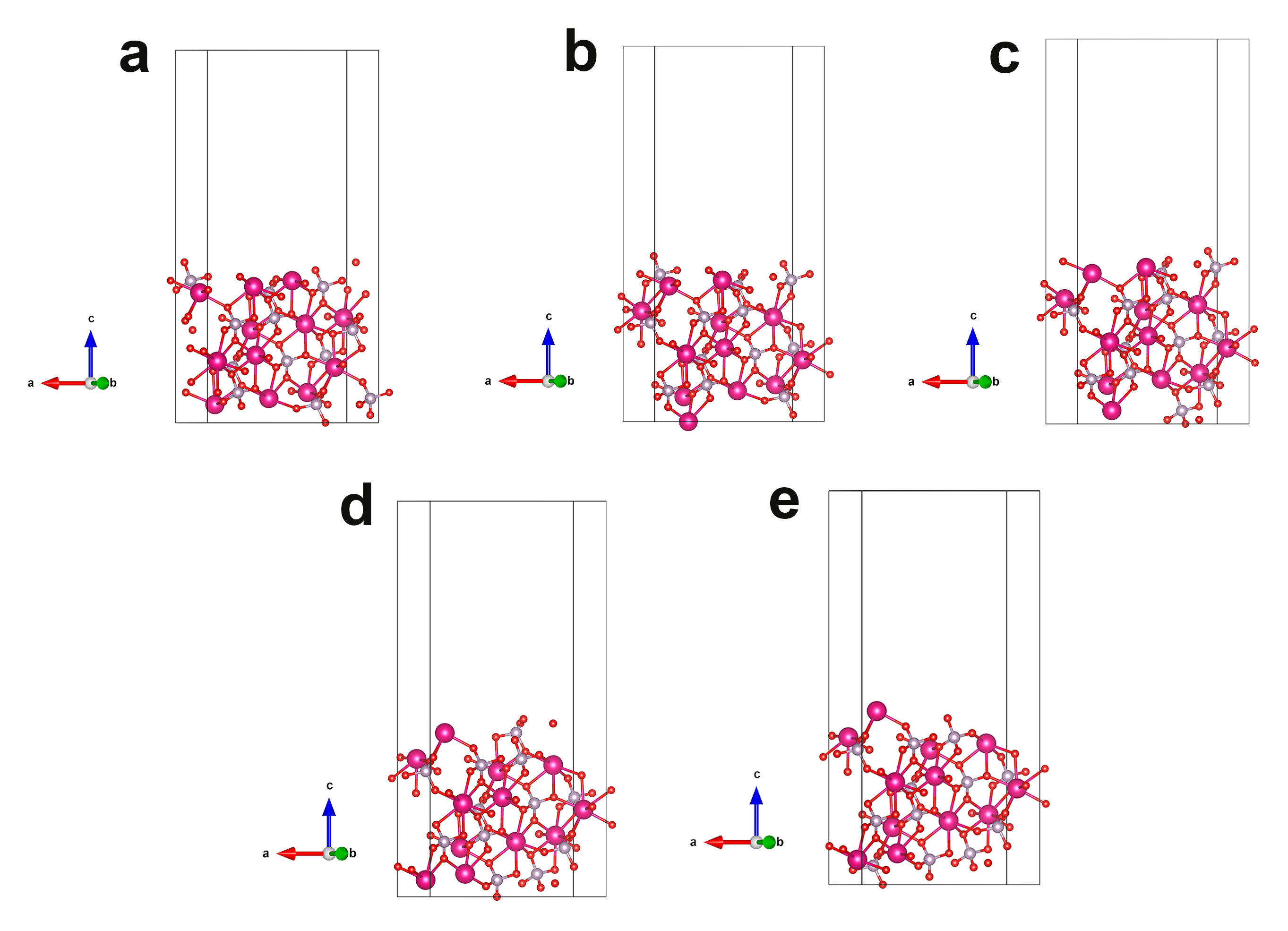
**

**Figure S30**. Five possible structures from SmPO_4_ (102) facets with different possible terminal atoms, where the grey, red, and rose red sphere represents the P, O, and Sm atom, respectively.

**
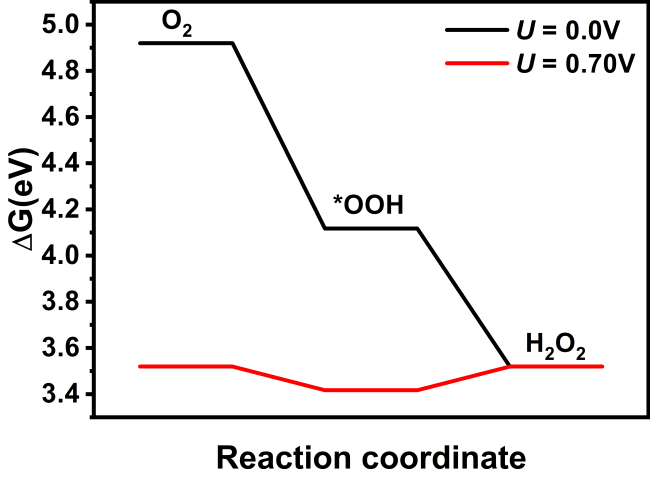
**

**Figure S31.** Free energy diagram of 2e^−^ ORR on SmPO_4_ -OOH system at U = 0 V and U = 0.70 V.


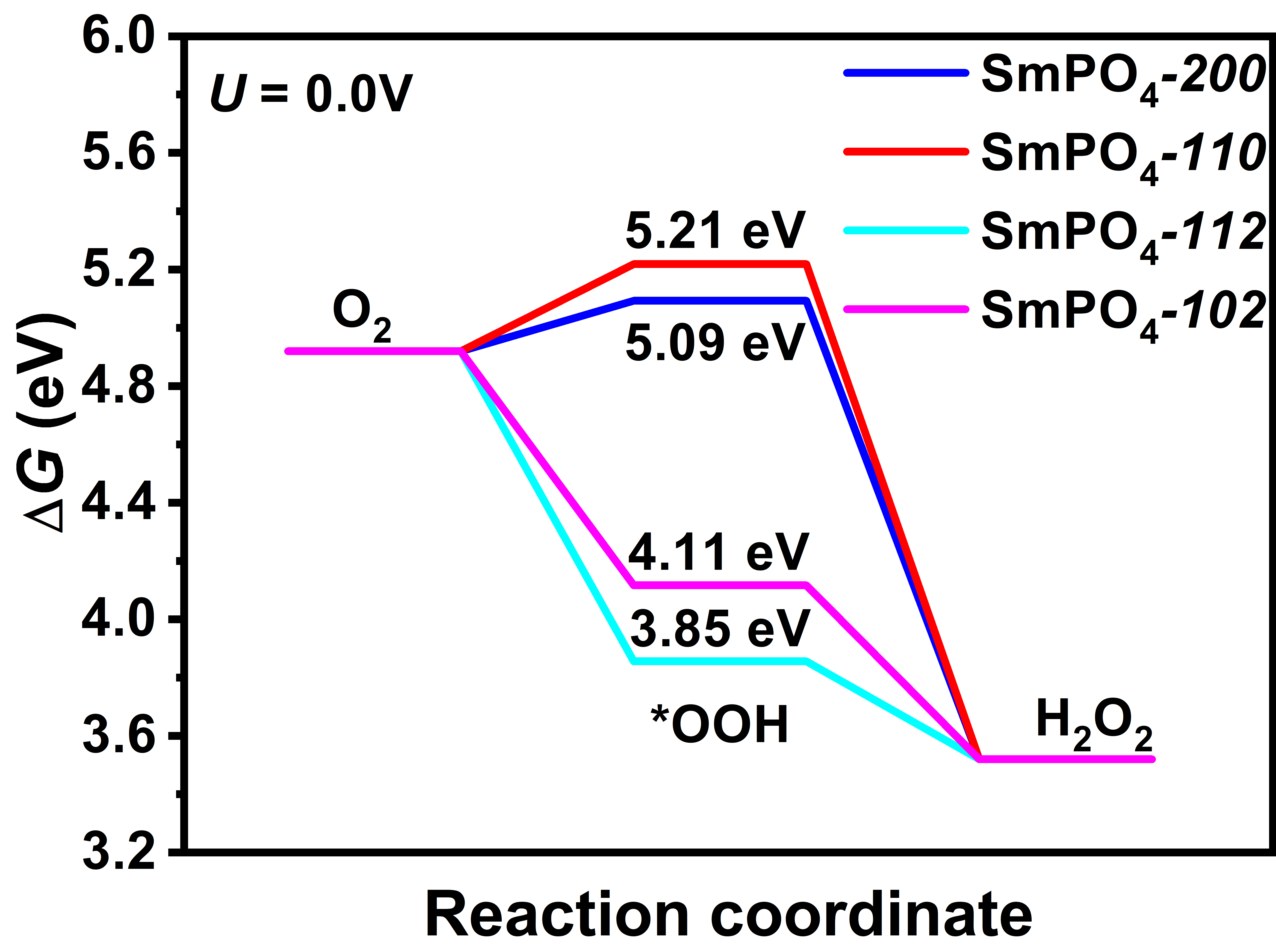


**Figure S32**. Free energy diagram of 2e^−^ ORR on different crystal faces of SmPO_4_ at U = 0.0 V.


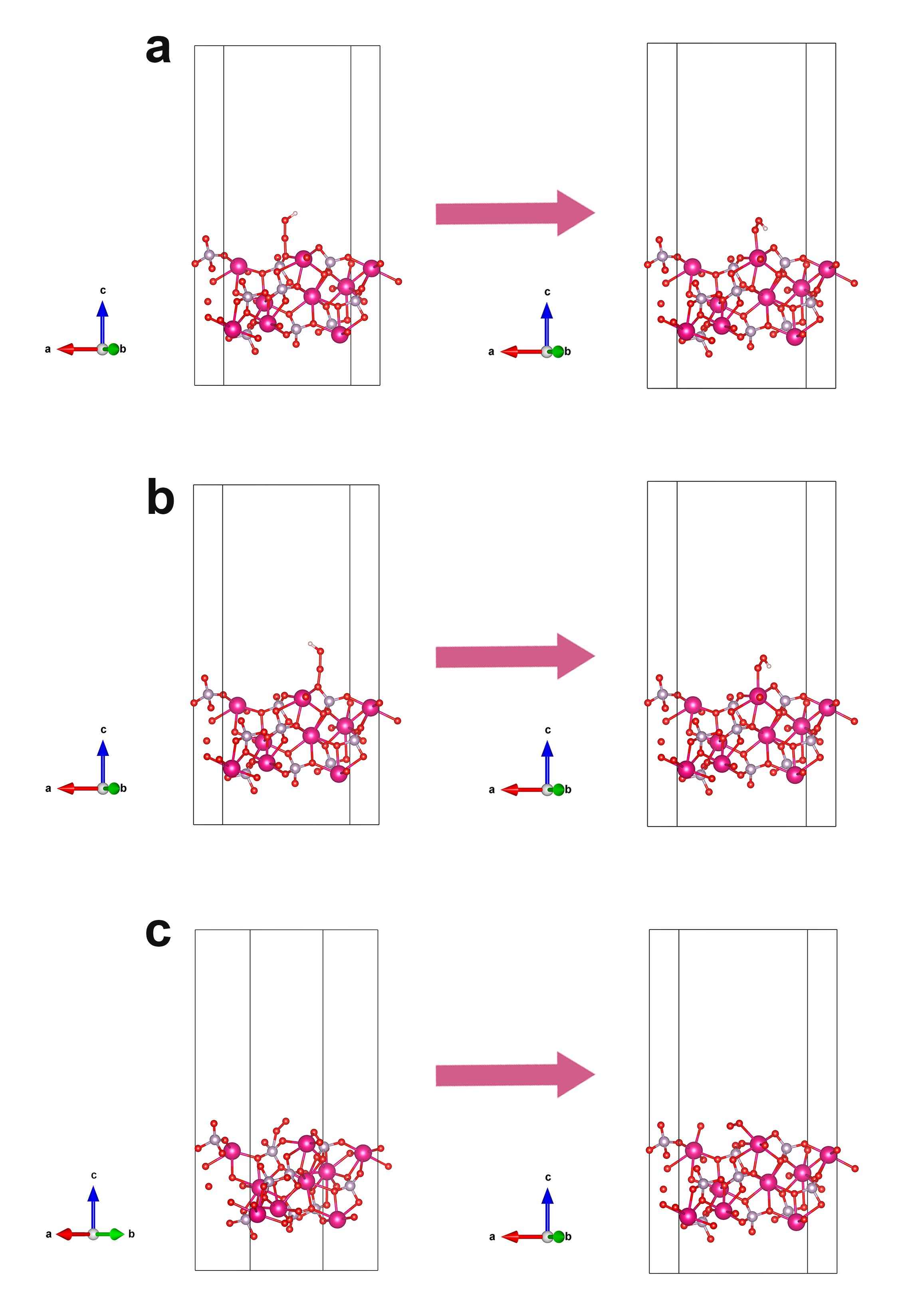


**Figure S33**. (a, b) The structural models of the adsorption of *OOH on the O atoms before and after structural relaxation calculation. (c) The adsorption of *OO on the P atoms before and after structural relaxation calculation. The grey, pink, red, and rose red sphere represents the P, H, O, and Sm atom, respectively.


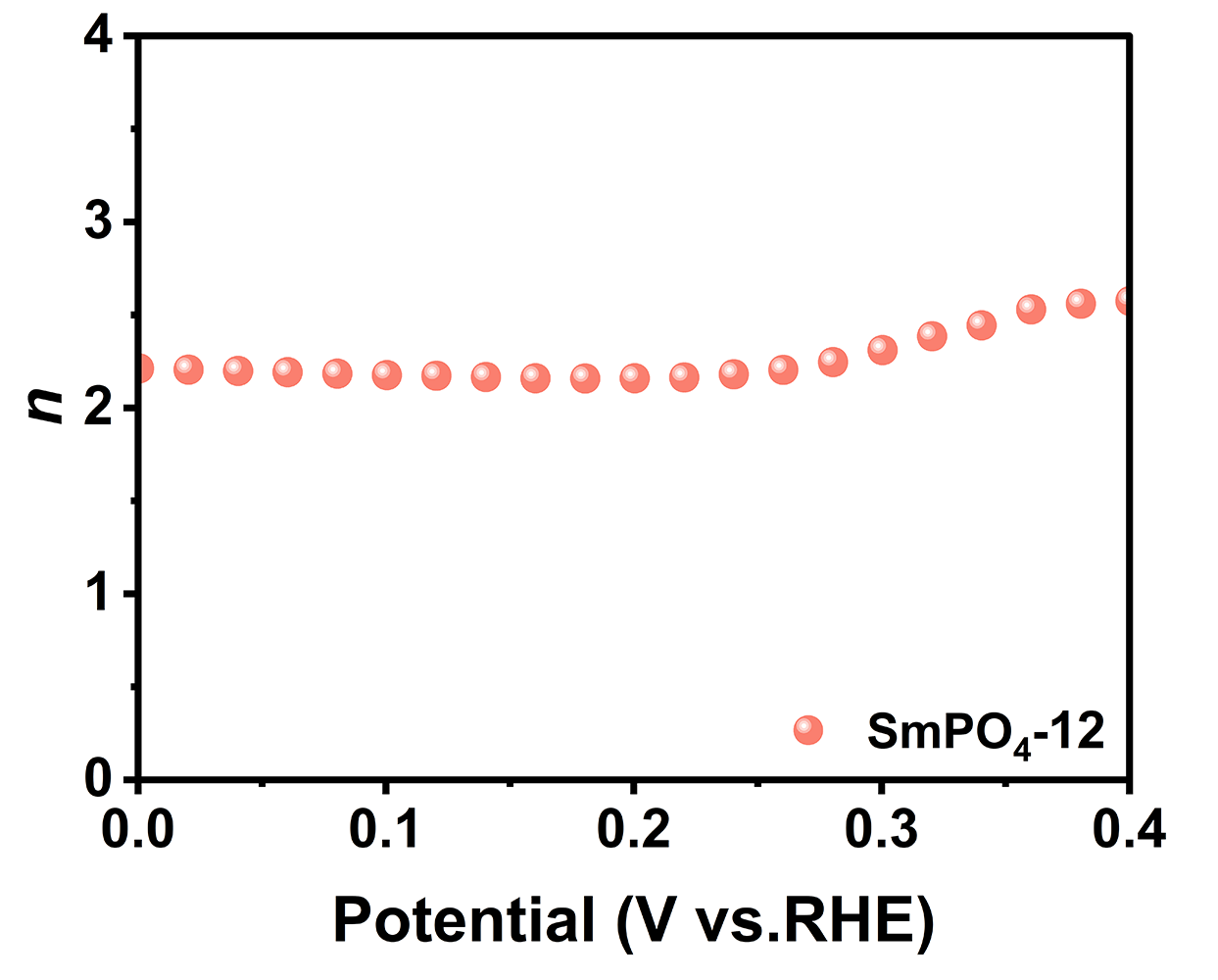


**Figure S34**. Calculated electron transfer number (n) within the potential sweep (neutral).

**
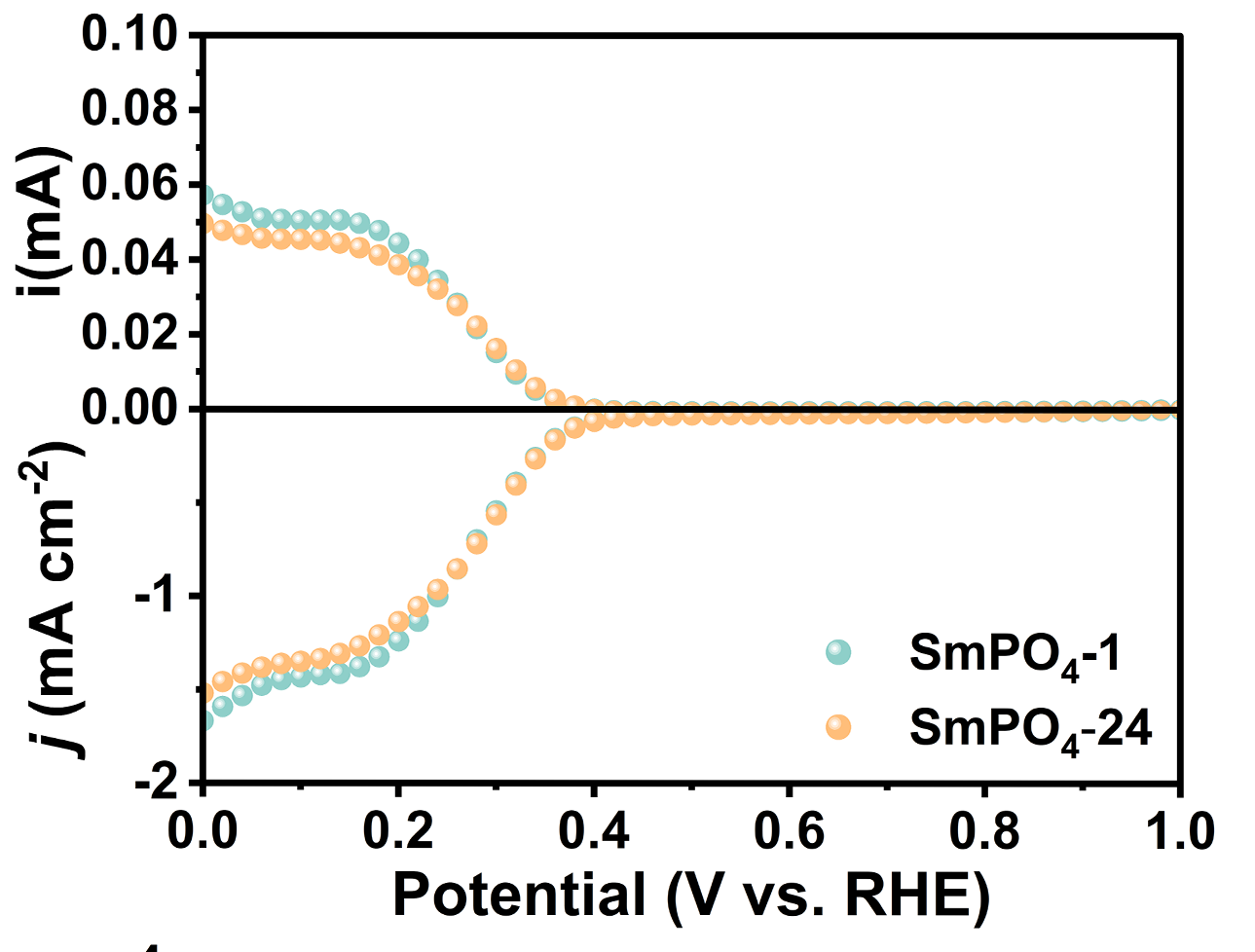
**

**Figure S35.** LSV curves of SmPO_4_ -1 and SmPO_4_ -24 recorded at 1600 rpm with a scan rate of 10 mV s^−1^ (bottom part), together with the corresponding H_2_O_2_ current on the ring electrode (upper part) in O_2_-saturated 0.1 M K_2_SO_4_.

**
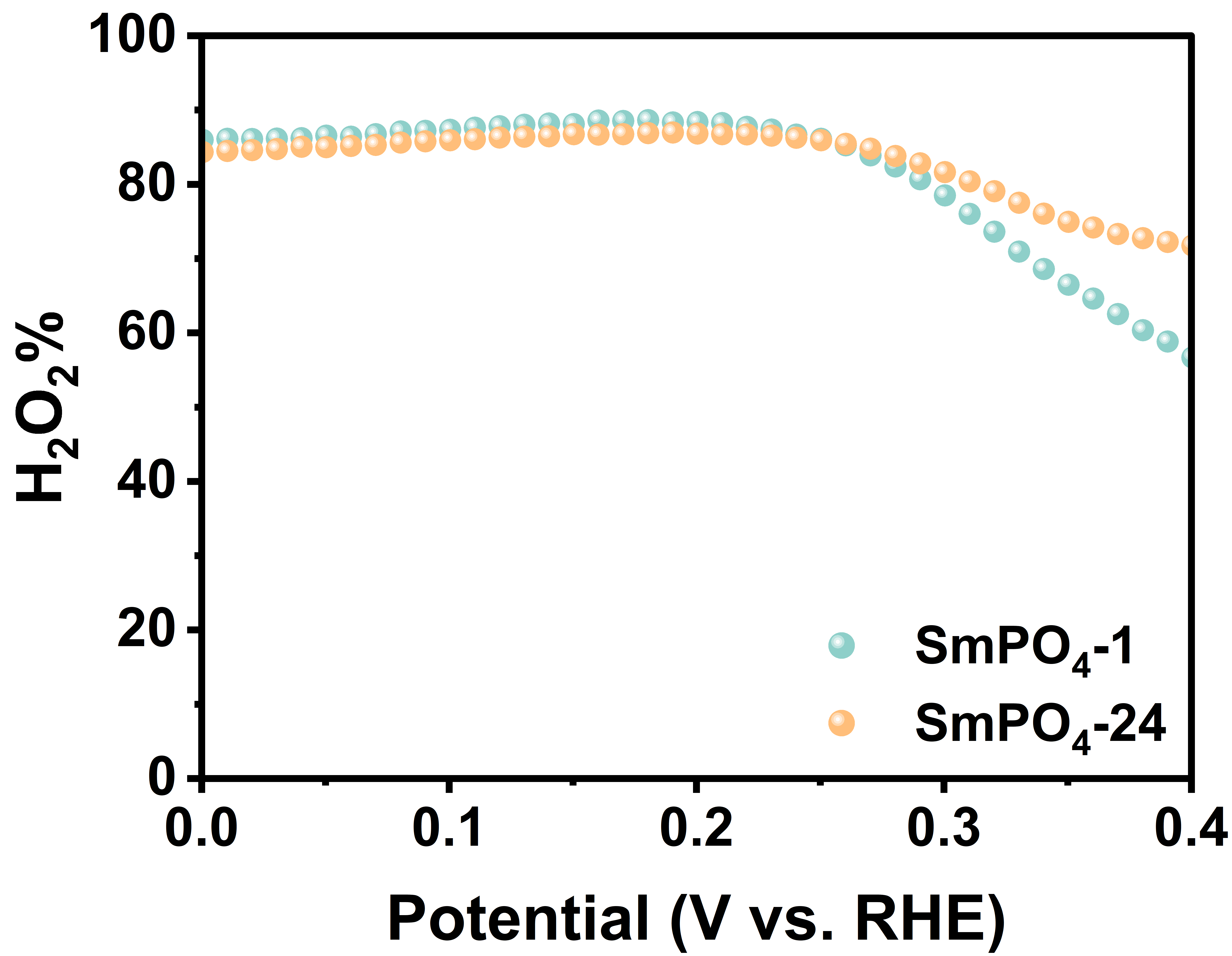
**

**Figure S36.** Selectivity of H_2_O_2_ within the potential sweep (neutral).

**
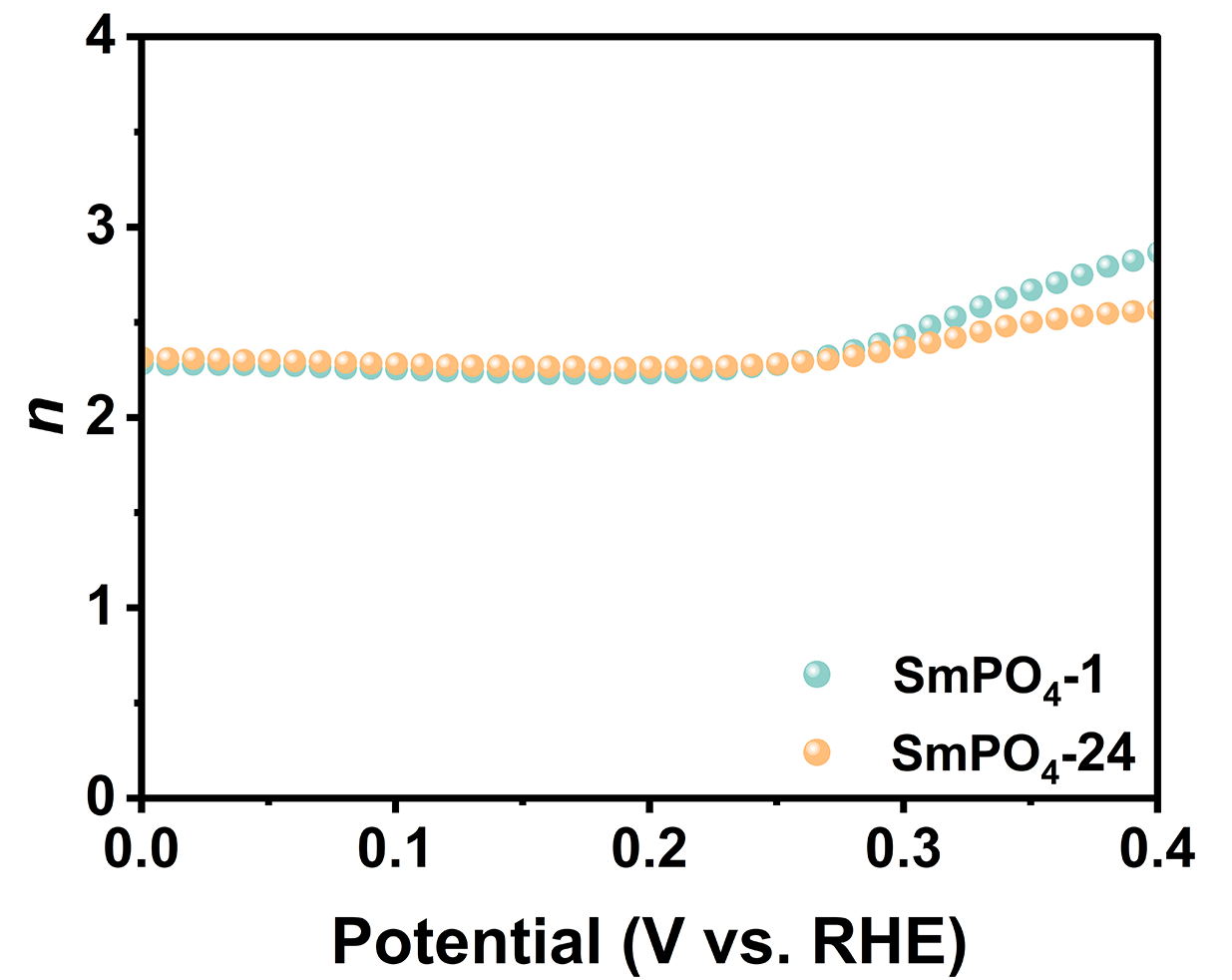
**

**Figure S37.** Calculated electron transfer number (*n*) within the potential sweep (neutral).


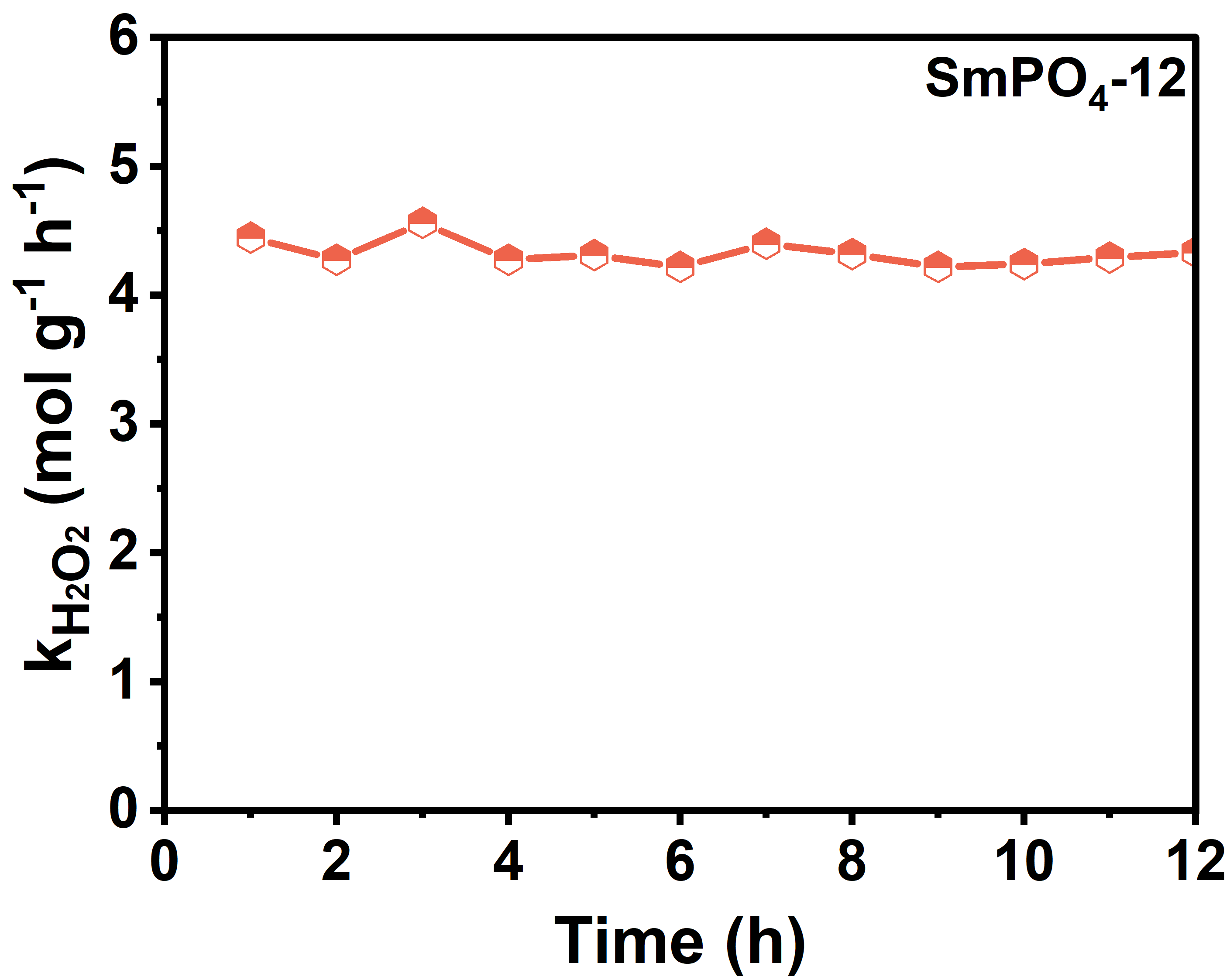


**Figure S38**. The number of moles of H_2_O_2_ produced by SmPO_4_-12 with the electrolytic time and the mass of the catalyst under the O_2_ condition in 1 M Na_2_SO_4_ in the gas diffusion electrode.


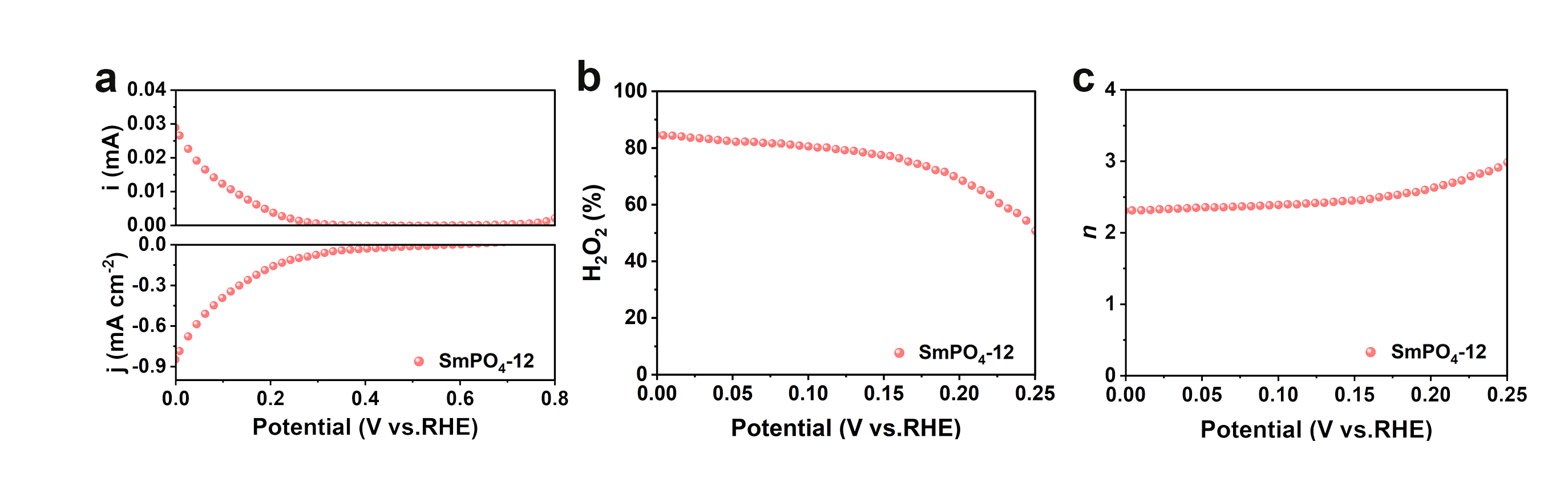


**Figure S39**. (a) LSV curves of SmPO_4_-12 recorded at 1600 rpm with a scan rate of 10 mV s^−1^ (bottom part) in 0.1 M HClO_4_, together with the corresponding H_2_O_2_ current on the ring electrode (upper part). under acidic conditions (b) Selectivity of H_2_O_2_ and (c) calculated electron transfer number (*n*) within the potential sweep


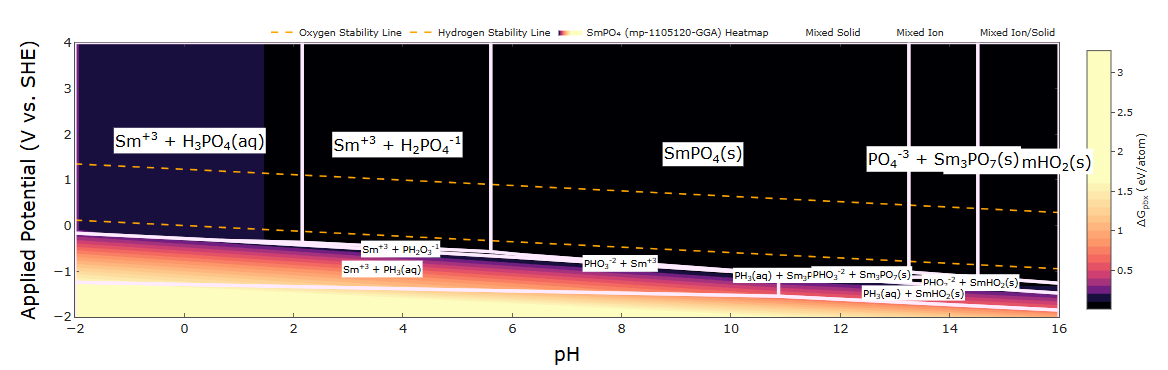


**Figure S40.** The Pourbaix Diagram of SmPO_4_ ^[7]^.


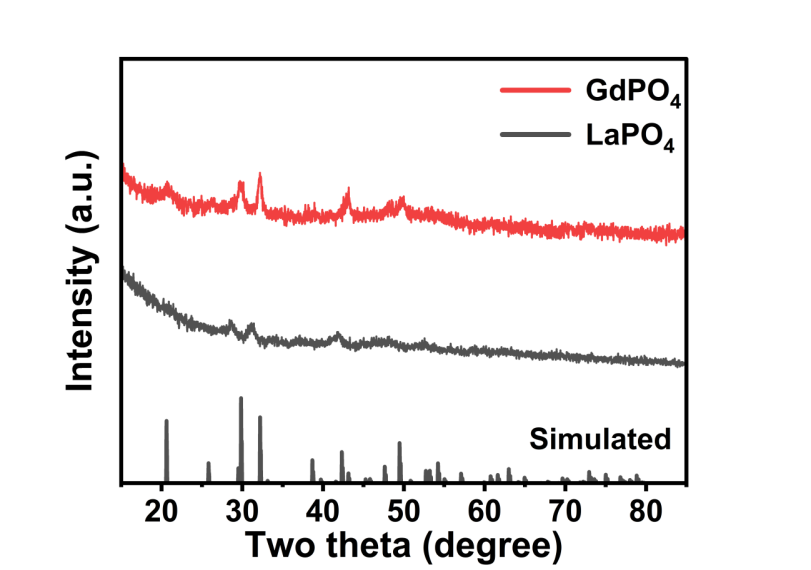


**Figure S41**. The XRD pattern of LaPO_4_ and GdPO_4_.


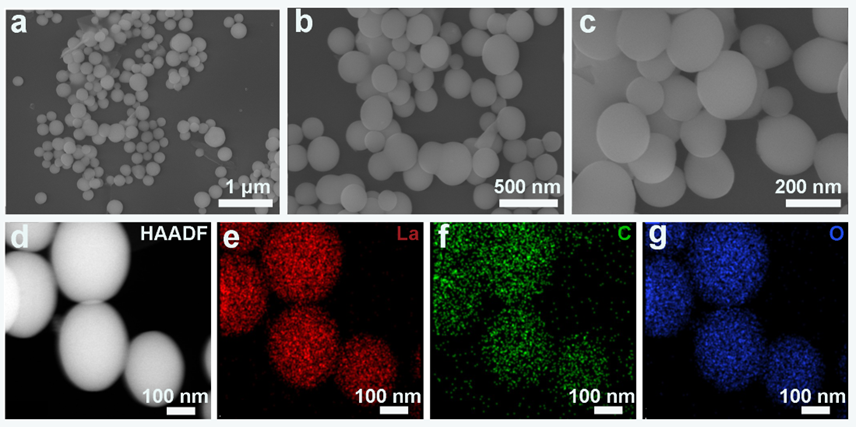


**Figure S42**. (a−c) The FESEM images of La(OH)CO_3_. (d) HAADF image of representative La(OH)CO_3_ nanosphere and the corresponding EDX elemental mapping of (e) La, (f) C and (g) O species.


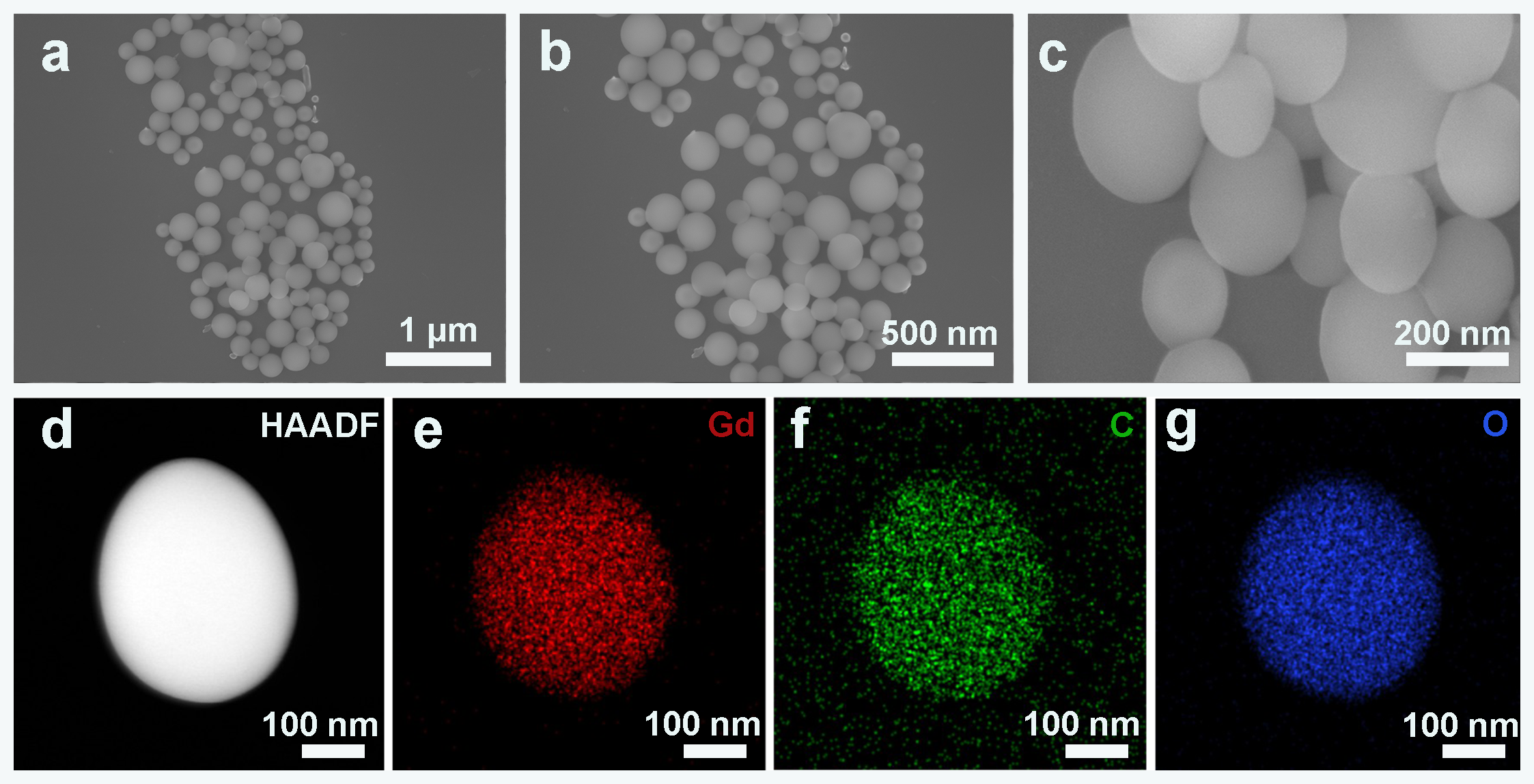


**Figure S43**. (a−c) The FESEM images of Gd(OH)CO_3_ nanosphere. (d) HAADF image of representative Gd(OH)CO_3_ nanosphere and the corresponding EDX elemental mapping of (e) Gd, (f) C and (g) O.


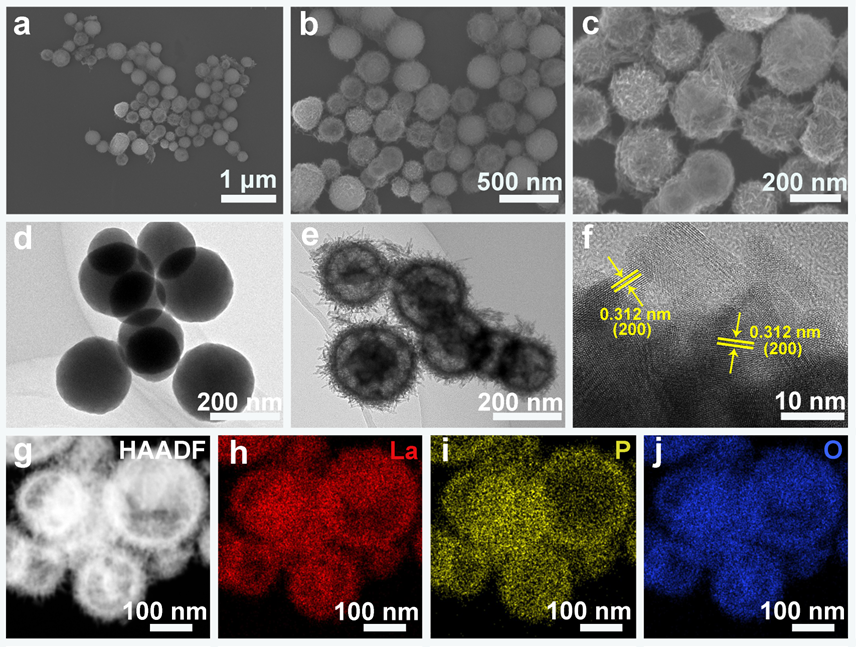


**Figure S44**. (a−c) The FESEM images of LaPO_4_, (d) TEM images of La(OH)CO_3_. (e) TEM and (f) high-resolution TEM images of LaPO_4_. (g) HAADF image of representative LaPO_4_ nanosphere and the corresponding EDX elemental mapping of (h) La, (i) P and (j) O species.


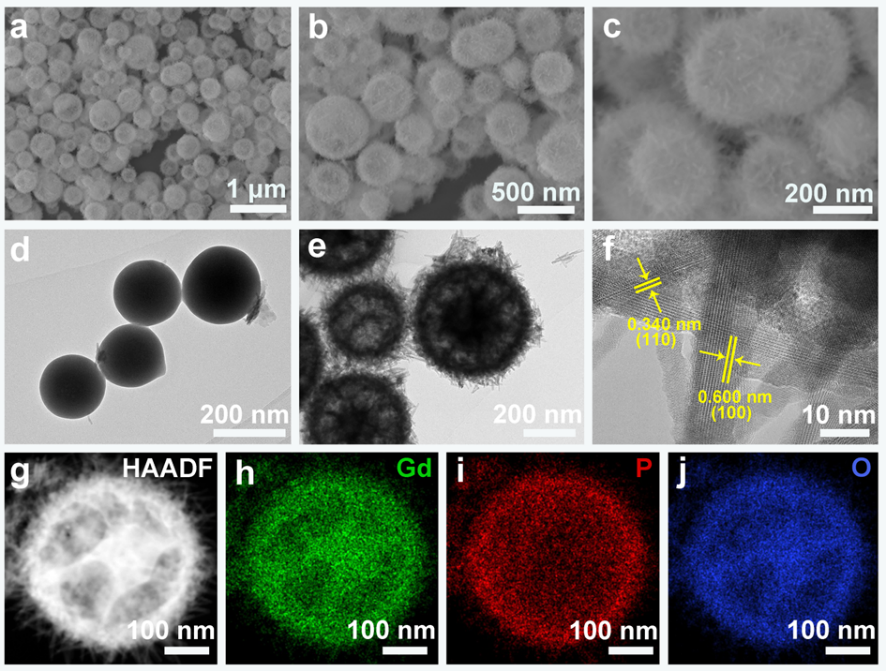


**Figure S45**. (a−c) The FESEM images of GdPO_4_, (d) TEM image of Gd(OH)CO_3_. (e) TEM and (f) high-resolution TEM images of GdPO_4_. (g) HAADF image of representative GdPO_4_ nanosphere and the corresponding EDX elemental mapping of (h) Gd, (i) P and (j) O species.


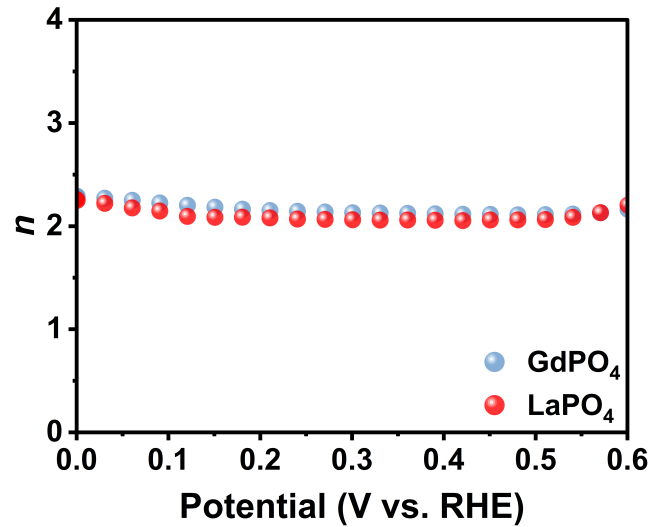


**Figure S46.** Calculated electron transfer number (*n*) within the potential sweep (alkaline).


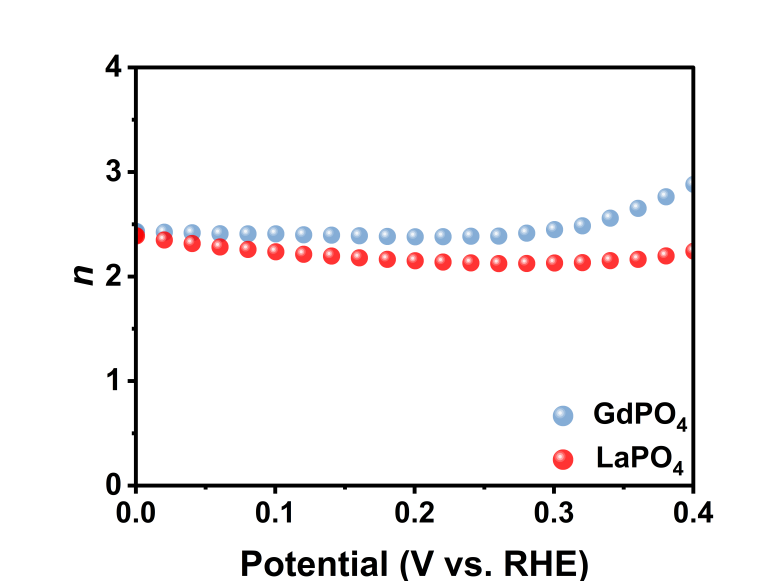


**Figure S47.** Calculated electron transfer number (*n*) within the potential sweep (neutral).


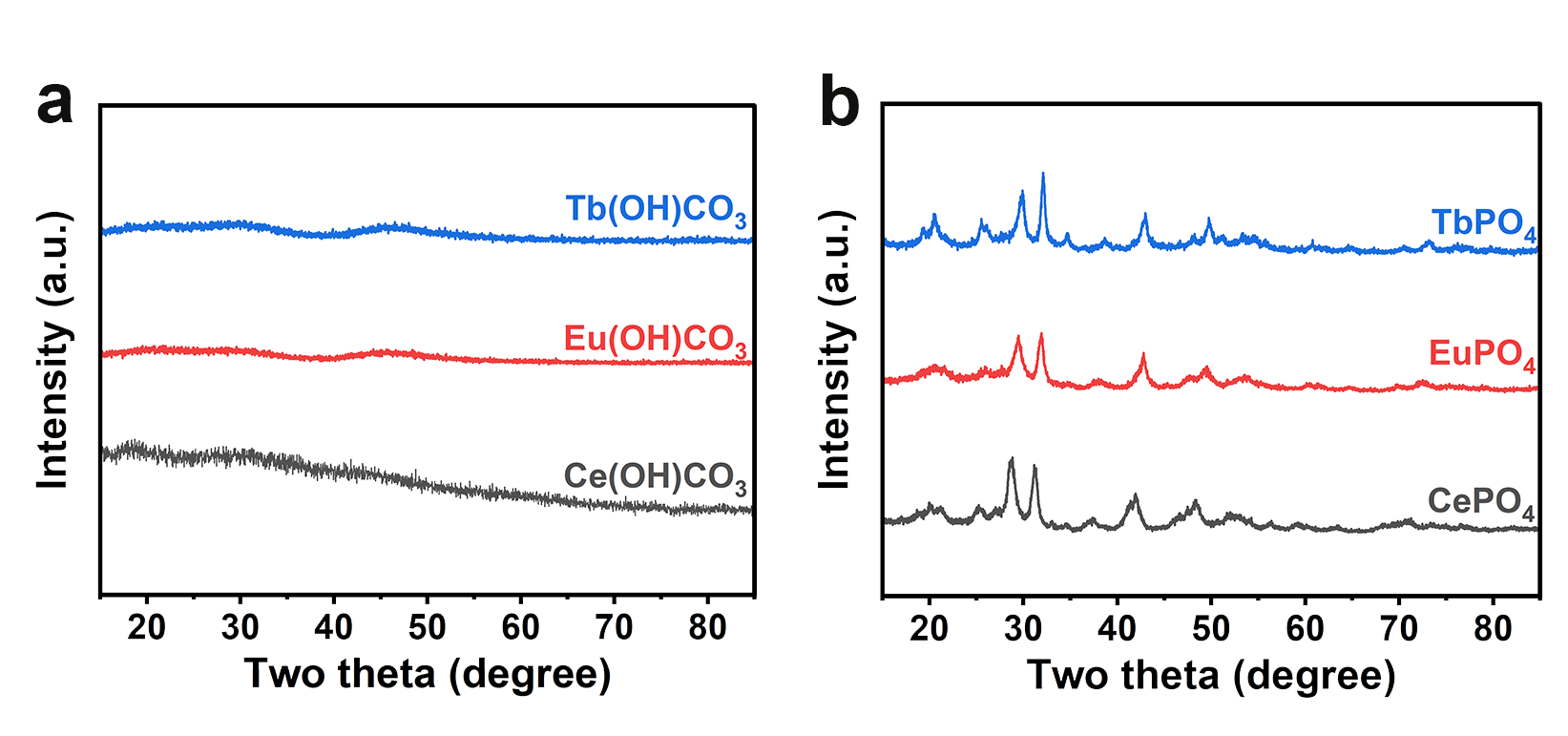


**Figure S48** The of XRD patterns of (a) Re(OH)CO_3_ (Re = Tb, Eu, Ce) precusors and (b) corresponding phosphorized products. It can be seen that the minor diffraction peaks appearing at 19° and 25° in Figure S48b may correspond to the RePO_4_ phase with another crystal structure (PDF#97-01-00624).


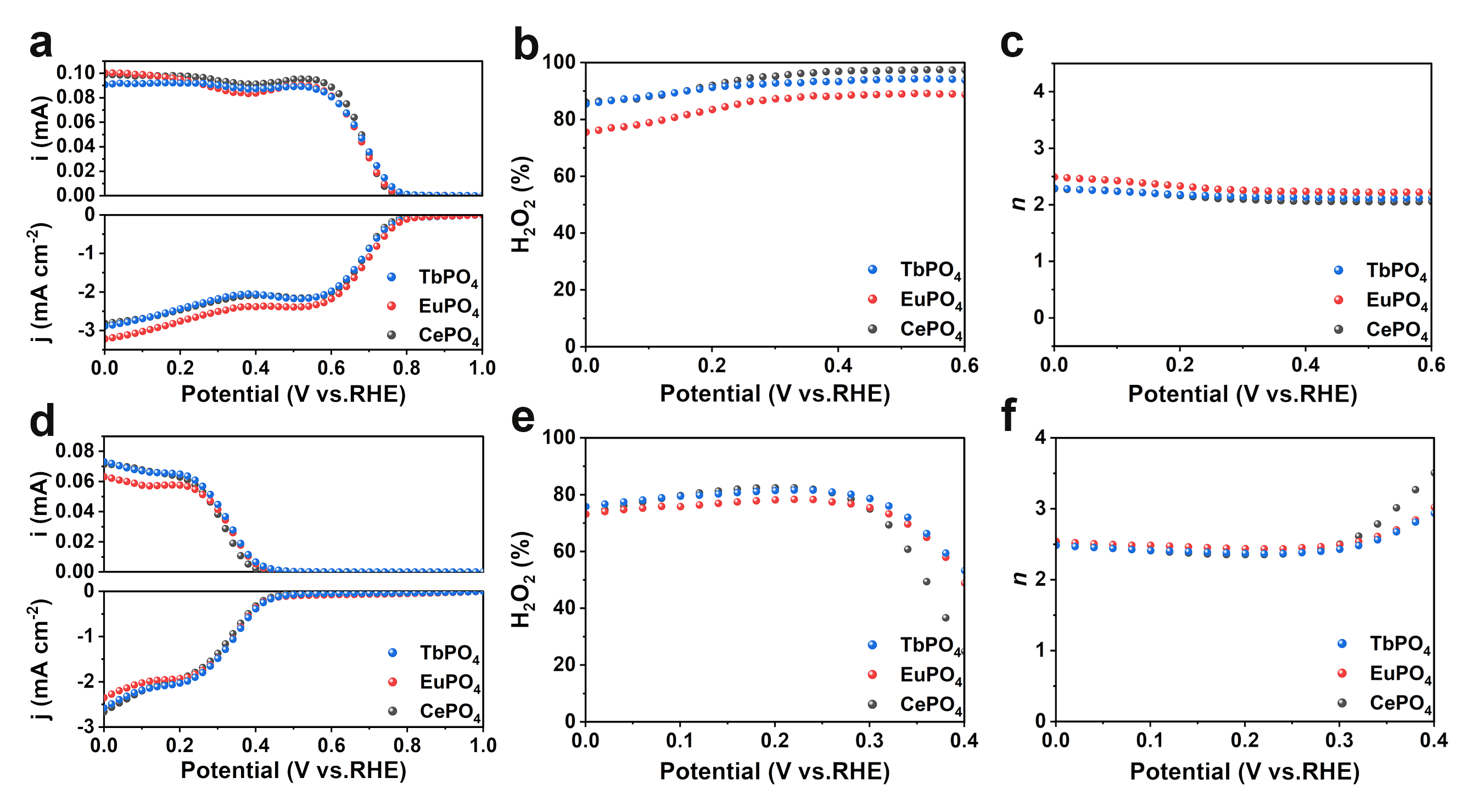


**Figure S49.** (a) LSV curves of RePO_4_ (Re = Tb, Eu, Ce) recorded at 1600 rpm with a scan rate of 10 mV s^−1^ (bottom part), together with the corresponding H_2_O_2_ current on the ring electrode (upper part) in O_2_-saturated 0.1 M KOH. (b) Selectivity of H_2_O_2_ and (c) calculated electron transfer number within the potential sweep.(d) LSV curves of RePO_4_ (Re = Tb, Eu, Ce) recorded at 1600 rpm with a scan rate of 10 mV s^−1^ (bottom part), together with the corresponding H_2_O_2_ current on the ring electrode (upper part) in O_2_-saturated 0.1 M K_2_SO_4_. (e) Selectivity of H_2_O_2_ and (f) calculated electron transfer number within the potential sweep.


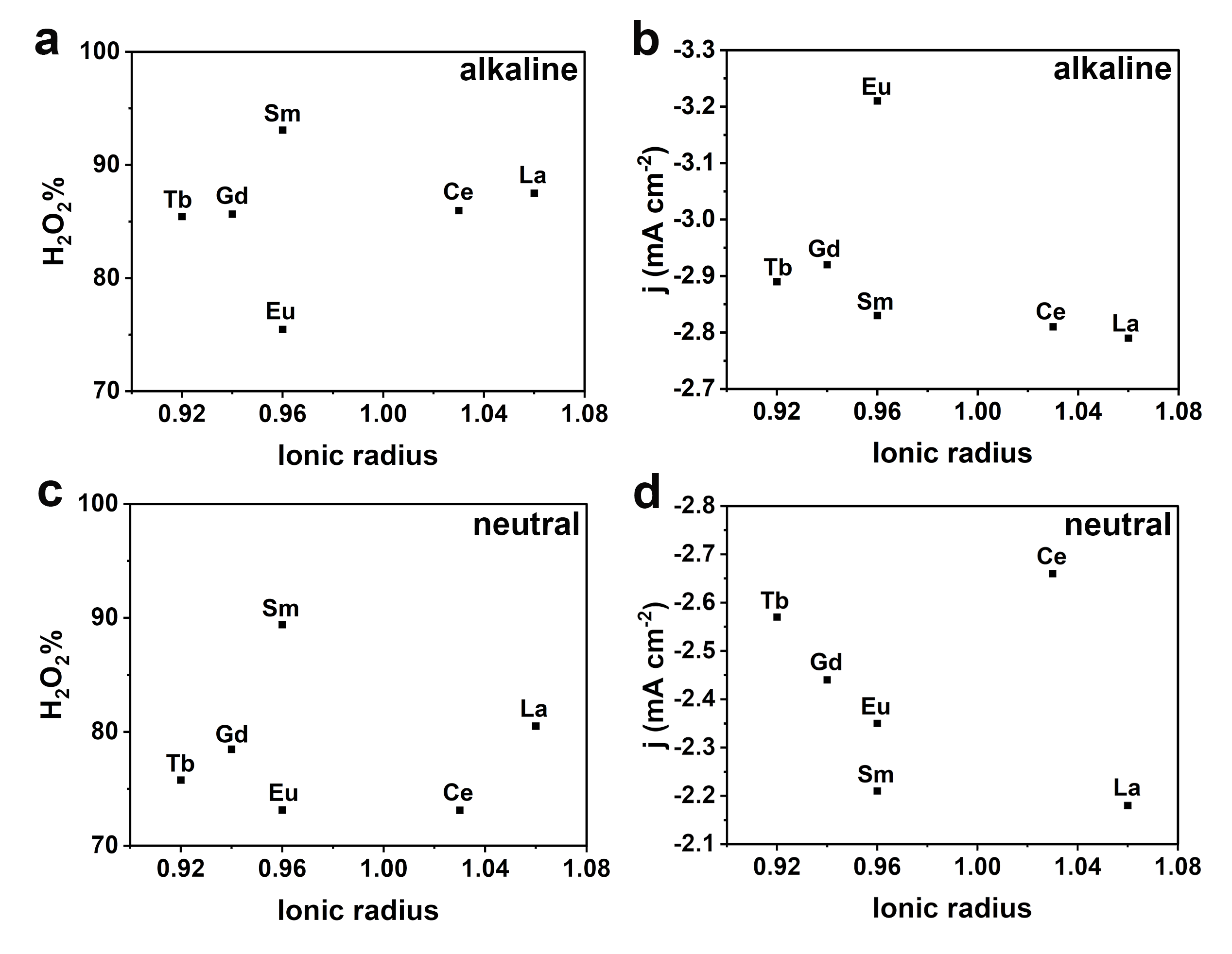


**Figure S50.** (a) H_2_O_2_ selectivity of different rare earth phosphates against the ionic radius of rare earth metal in oxygen-saturated 0.1 M KOH (at 0 V *vs.* RHE) and (b) Current densities of different rare earth phosphates against the ionic radius of rare earth metal in oxygen-saturated 0.1 M KOH (at 0 V *vs*. RHE). (c) H_2_O_2_ selectivity of different rare earth phosphatesagainst the ionic radius of rare earth metal in oxygen-saturated 0.1 M K_2_SO_4_ (at 0 V *vs*. RHE) and (d) Current densities of different rare earth phosphates against the ionic radius of rare earth metal in oxygen-saturated 0.1 M K_2_SO_4_ (at 0 V *vs*. RHE).

**TABLES**

**Table S1.** Structural parameters for SmPO_4_-12 compounds refined from the experimental XRD pattern.

| Sample | Space group | Lattice parameters (Å) | | | Abundance (wt.%) |
| --- | --- | --- | --- | --- | --- |
|  |  | *a* | *b* | *c* |  |
| SmPO_4_ | *P*3_2_21 | 6.963 (9) | 6.963 (9) | 6.361 (7) | ~100 |

**Table S2.** Atomic coordinates, isotropic thermal parameters (*B* values), and occupation numbers (*g* values) for the SmPO_4_-12 structure determined from XRD data.

| atom | *g* | *x* | *y* | *z* | *B* (nm^2^) |
| --- | --- | --- | --- | --- | --- |
| Sm | 1 | 0.460(1) | 0 | 0.66670 | 0.011(4) |
| P | 1 | 0.406(6) | 0 | 0.187(2) | 0.008(9) |
| O | 1 | 0.364(5) | 0.527(6) | 0.361(4) | 0.075(2) |
| O | 1 | 0.776(3) | 0.821(7) | 0.60632(4) | 0.057(1) |

**Table S3.** EXAFS fitting parameters for SmPO_4_-12 samples.

| Sample | Path | *N* | *R* (Å) | Δ*E*_0_ (eV) | *σ*^2^(Å^2^) | *R* factor |
| --- | --- | --- | --- | --- | --- | --- |
| SmPO_4_ | Sm-O1 | 4.0 ± 0.2 | 2.39 ± 0.01 | 6.4 ± 0.7 | 0.0042 ± 0.0007 | 0.015 |
|  | Sm-O2 | 3.9 ± 0.4 | 2.52 ± 0.01 | 4.8 ± 0.8 | 0.0026 ± 0.0013 |  |
|  | Sm-P | 5.8 ± 0.6 | 3.62 ± 0.02 | -3.4 ± 1.4 | 0.0106 ± 0.0013 |  |
|  | Sm-Sm | 2.2 ± 0.5 | 4.12 ± 0.03 | 7.6 ± 2.5 | 0.0028 ± 0.0014 |  |

**Table S4.** The comparison of 2e^−^ ORR performance of SmPO_4_-12 with those of recently reported metal-based electrocatalysts in alkaline solution.

| Material | Electrolyte | Selectivity | Potential | Ref. |
| --- | --- | --- | --- | --- |
| Au@Cu_1.75_S-CNTs | 0.1M KOH | ~93 | 0.6-0.7 V | [8] |
| a-TiO_2−x_/TiC | 0.1M KOH | ~92 | 0.5-0.6 V | [9] |
| Br−Ni MOF | 0.1M KOH | ~90 | 0.4-0.5V | [10] |
| PC-O/IT | 0.1M KOH | ~59 | 0.45-0.55 | [11] |
| Cu-TiO_2_ | 0.1M KOH | ~91 | 0.3-0.5 | [12] |
| Co_1_@GO | 0.1M KOH | ~81 | 0.55-0.6 | [13] |
| Co−N−C | 0.1M KOH | ~64 | 0.3 | [14] |
| V_2_CTX | 0.1M KOH | ~90 | 0.4-0.6 | [15] |
| O-Mo_2_TiC_2_ | 0.1M KOH | ~84 | 0.2-0.5 | [16] |
| Ba_0.5_Sr_0.5_Fe_0.95_Cu_0.05_O_3-δ_ | 0.1M KOH | ~68 | 04-0.5 | [17] |
| ZnSnO_3_ | 0.1M KOH | ~76 | 0.3-0.5 | [18] |
| ZnO | 0.1M KOH | ~71 | 0.3-0.5 | [18] |
| Ni_4_-B_1_@BNC | 0.1M KOH | ~86 | 0.1-0.35 | [19] |
| Mn-TiO_2_ | 0.1M KOH | ~92 | 0.4-0.6 | [20] |
| Ni−N−C | 0.1M KOH | ~75 | 0.4-0.6 | [21] |
| Gd-CBO/CuO | 0.1M KOH | ~82 | 0.2-0.3 | [22] |
| Cu/GOE | 0.1M KOH | ~77 | 0.4-0.5 | [23] |
| V_2_O_5_-300 | 0.1M KOH | ~92 | 0.1-0.3 | [24] |
| Co-POC-O | 0.1M KOH | ~84 | 0.65-0.8 | [25] |
| Ni MOF NSs-8 | 0.1M KOH | ~85 | 0.2-0.45 | [26] |
| Fe_3_O_4_/graphehe | 1M KOH | ~63 | 0.45-0.6 | [27] |
| Ni–SA/G–0 | 0.1M KOH | ~94 | 0.4-0.5 | [28] |
| Cu_7.2_Se_4_ | 0.1M KOH | ~90 | 0.2-0.6 | [29] |
| Mn–O/N@NCs-20 | 0.1M KOH | ~70 | 0.3-0.5 | [30] |
| Au-Pt-Ni | 0.1M KOH | ~91 | 0.4-0.6 | [31] |
| NiCoPhi/CNT | 0.1M KOH | ~85 | \ | [32] |
| B-C | 0.1M KOH | ~90 | 0.6-0.7 | [33] |
| Ni_3_(HITP)_2_ | 0.1M KOH | ~63 | 0.55-0.75 | [34] |
| NiNx/C-AQNH_2_ | 0.1M KOH | ~82 | 0.6-0.8 | [35] |
| S-NiP_4_Mo_6_ | 0.1M KOH | ~84 | 0.5-0.75 | [36] |
| Co-N_2_-C/HO | 0.1M KOH | ~91 | 0.45-0.75 | [37] |
| SmPO_4_-12 | 0.1M KOH | ~96 | 0-0.6 | This work |

**Table S5.** EXAFS fitting parameters for post-ORR SmPO_4_-12 samples.

| Sample | Path | *N* | *R* (Å) | Δ*E*_0_ (eV) | *σ*^2^(Å^2^) | *R* factor |
| --- | --- | --- | --- | --- | --- | --- |
| SmPO_4_ | Sm-O1 | 4.1 ± 0.4 | 2.39 ± 0.01 | 9.3 ± 0.3 | 0.001 ± 0.016 | 0.017 |
|  | Sm-O2 | 3.9 ± 0.3 | 2.55 ± 0.01 | 6.7 ± 0.7 | 0.006 ± 0.009 |  |
|  | Sm-P | 6.1 ± 0.6 | 3.64 ± 0.02 | -3.2 ± 1.0 | 0.013 ± 0.018 |  |
|  | Sm-Sm | 2.3 ± 0.3 | 4.17 ± 0.03 | 8.4 ± 1.1 | 0.008 ± 0.001 |  |

**Table S6.** The variation amount of Sm species in the electrolyte before and after ORR CA determined by Inductively coupled plasma-atomic emission spectrometry (ICP). It should be noted that the observed variation in the data may fall within the margin of measurement error.

| Electrolyte | Measured element | Instrument reading | unit |
| --- | --- | --- | --- |
| pre-reaction | Sm | 0.0000048 | g L^−1^ |
| post-reaction | Sm | 0.0000007 | g L^−1^ |
| pre-reaction | P | 0.0000101 | g L^−1^ |
| post-reaction | P | 0.0000133 | g L^−1^ |

**References**

[1] F. Izumi, T. Ikeda, *Mater. Sci*. *Forum*, **2000**, *321-324*, 198.

[2] G. Kresse, D. Joubert, *Phys. Rev. B* **1999**, *59*, 1758.

[3] G. Kresse, J. Furthmüller, *Phys. Rev. B* **1996**, *54*, 11169.

[4] J. P. Perdew, K. Burke, M. Ernzerhof, *Phys. Rev. Lett.* **1996**, *77*, 3865.

[5] D. Sheppard, P. Xiao, W. Chemelewski, D. D. Johnson, G. Henkelman, *J. Chem. Phys.* **2012**, *136*, 074103.

[6] I. Yagi, K. Inokuma, K. i. Kimijima, H. Notsu, *J. Phys. Chem. C* **2014**, *118*, 26182.

[7] Data retrieved from the Materials Project for SmPO_4_ (mp-1105120) from data-base version v2022.10.28. *https://next-gen.materialsproject.org/materials/mp-1105120?for-mula=SmPO_4_*

[8] A. Zhang, Y. Liu, J. Wu, J. Zhu, S. Cheng, Y. Wang, Y. Hao, S. Zeng, *Chem. Eng. J.* **2023**, *454*, 140317.

[9] Z. Xu, J. Liang, Y. Wang, K. Dong, X. Shi, Q. Liu, Y. Luo, T. Li, Y. Jia, A. M. Asiri, Z. Feng, Y. Wang, D. Ma, X. Sun, *ACS Appl. Mater. Interfaces* **2021**, *13*, 33182.

[10] M. Liu, Y. Li, Z. Qi, H. Su, W. Cheng, W. Zhou, H. Zhang, X. Sun, X. Zhang, Y. Xu, Y. Jiang, Q. Liu, S. Wei, *J. Phys. Chem. Lett.* **2021**, *12*, 8706.

[11] Z. Chen, Y. Li, M. Wu, Y. Cao, *Energy Fuels* **2021**, *35*, 2665.

[12] Z. Deng, L. Li, Y. Ren, C. Ma, J. Liang, K. Dong, Q. Liu, Y. Luo, T. Li, B. Tang, Y. Liu, S. Gao, A. M. Asiri, S. Yan, X. Sun, *Nano Res.* **2022**, *15*, 3880.

[13] B.-W. Zhang, T. Zheng, Y.-X. Wang, Y. Du, S.-Q. Chu, Z. Xia, R. Amal, S.-X. Dou, L. Dai, *Commun. Chem.* **2022**, *5*, 43.

[14] Y. Sun, L. Silvioli, N. R. Sahraie, W. Ju, J. Li, A. Zitolo, S. Li, A. Bagger, L. Arnarson, X. Wang, T. Moeller, D. Bernsmeier, J. Rossmeisl, F. Jaouen, P. Strasser, *J. Am. Chem. Soc.* **2019**, *141*, 12372.

[15] X. Huang, M. Song, J. Zhang, J. Zhang, W. Liu, C. Zhang, W. Zhang, D. Wang, *Nano Res.* **2022**, *15*, 3927.

[16] G. Li, B. Zhou, P. Wang, M. He, Z. Fang, X. Yuan, W. Wang, X. Sun, Z. Li, *Catalysts* **2022**, *12*, 850

[17] S. V. Venkatesan, A. H. B. Mostaghimi, V. Thangadurai, S. Siahrostami, *Electrochem. Sci. Adv.* **2023**, *3*, e2100140.

[18] J. Qian, W. Liu, Y. Jiang, Y. Mu, Y. Cai, L. Shi, L. Zeng, *ACS Sustainable Chem. Eng.* **2022**, *10*, 14351.

[19] H. Fu, N. Zhang, F. Lai, L. Zhang, Z. Wu, H. Li, H. Zhu, T. Liu, *Small* **2022**, *18*, 2203510.

[20] Q. Chen, C. Ma, S. Yan, J. Liang, K. Dong, Y. Luo, Q. Liu, T. Li, Y. Wang, L. Yue, B. Zheng, Y. Liu, S. Gao, Z. Jiang, W. Li, X. Sun, *ACS Appl. Mater. Interfaces* **2021**, *13*, 46659.

[21] L. Shahcheraghi, C. Zhang, H.-J. Lee, M. Cusack-Striepe, F. Ismail, A. Abdellah, D. C. Higgins, *J. Phys. Chem. C* **2021**, *125*, 15830.

[22] Z. Li, Q. Xu, F. Gou, B. He, W. Chen, W. Zheng, X. Jiang, K. Chen, C. Qi, D. Ma, *Nano Res.* **2021**, *14*, 3439.

[23] M. Nunes, D. M. Fernandes, M. V. Morales, I. Rodríguez-Ramos, A. Guerrero-Ruiz, C. Freire, *J. Appl. Electrochem.* **2019**, *49*, 693.

[24] S. Zhang, G. Feng, Z. Bao, X. Peng, C. Jiang, Y. Shao, S. Wang, J. Wang, *Ind. Eng. Chem. Res.* **2023**, *62*, 6113.

[25] B.-Q. Li, C.-X. Zhao, J.-N. Liu, Q. Zhang, *Adv. Mater.* **2019**, *31*, 1808173.

[26] M. Wang, X. Dong, Z. Meng, Z. Hu, Y.-G. Lin, C.-K. Peng, H. Wang, C.-W. Pao, S. Ding, Y. Li, Q. Shao, X. Huang, *Angew. Chem., Int. Ed.* **2021**, *60*, 11190.

[27] W. R. P. Barros, Q. Wei, G. Zhang, S. Sun, M. R. V. Lanza, A. C. Tavares, *Electrochim. Acta* **2015**, *162*, 263.

[28] X. Song, N. Li, H. Zhang, L. Wang, Y. Yan, H. Wang, L. Wang, Z. Bian, *ACS Appl. Mater. Interfaces* **2020**, *12*, 17519.

[29] Q. Yuan, J. Zhao, D. H. Mok, Z. Zheng, Y. Ye, C. Liang, L. Zhou, S. Back, K. Jiang, *Nano Lett.* **2022**, *22*, 1257.

[30] A. Byeon, J. Cho, J. M. Kim, K. H. Chae, H.-Y. Park, S. W. Hong, H. C. Ham, S. W. Lee, K. R. Yoon, J. Y. Kim, *Nanoscale Horiz.* **2020**, *5*, 832.

[31] Z. Zheng, Y. H. Ng, D.-W. Wang, R. Amal, *Adv. Mater.* **2016**, *28*, 9949.

[32] C. Sathiskumar, C. Alex, N. S. John, *ChemElectroChem* **2020**, *7*, 1935.

[33] Y. Xia, X. Zhao, C. Xia, Z. Y. Wu, P. Zhu, J. Y. T. Kim, X. Bai, G. Gao, Y. Hu, J. Zhong, Y. Liu, H. Wang, *Nat. Commun.* **2021**, *12*, 4225.

[34] E. M. Miner, T. Fukushima, D. Sheberla, L. Sun, Y. Surendranath, M. Dincă, *Nat. Commun.* **2016**, *7*, 10942.

[35] X. Li, S. Tang, S. Dou, H. J. Fan, T. S. Choksi, X. Wang, *Adv. Mater.* **2022**, *34*, 2104891.

[36] Y. Zheng, X. Xu, J. Chen, Q. Wang, *Appl. Catal., B* **2021**, *285*, 119788.

[37] H. Gong, Z. Wei, Z. Gong, J. Liu, G. Ye, M. Yan, J. Dong, C. Allen, J. Liu, K. Huang, R. Liu, G. He, S. Zhao, H. Fei, *Adv. Funct. Mater.* **2022**, *32*, 2106886.
